# Mono or double Pd-catalyzed C–H bond functionalization for the annulative π-extension of 1,8-dibromonaphthalene: a one pot access to fluoranthene derivatives

**DOI:** 10.3762/bjoc.20.37

**Published:** 2024-02-23

**Authors:** Nahed Ketata, Linhao Liu, Ridha Ben Salem, Henri Doucet

**Affiliations:** 1 University of Rennes, CNRS, ISCR-UMR 6226, F-35000 Rennes, France; 2 Organic chemistry laboratory, LR17ES08, Department of Chemistry, Faculty of Sciences, University of Sfax, B.P. 1171, 3038, Sfax, Tunisia

**Keywords:** catalysis, C–H bond functionalization, direct arylation, fluoranthenes, palladium

## Abstract

The Pd-catalyzed annulative π-extension of 1,8-dibromonaphthalene for the preparation of fluoranthenes in a single operation has been investigated. With specific arenes such as fluorobenzenes, the Pd-catalyzed double functionalization of C–H bonds yields the desired fluoranthenes. The reaction proceeds via a palladium-catalyzed direct intermolecular arylation, followed by a direct intramolecular arylation step. As the C–H bond activation of several benzene derivatives remains very challenging, the preparation of fluoranthenes from 1,8-dibromonaphthalene via Suzuki coupling followed by intramolecular C–H activation has also been investigated to provide a complementary method. Using the most appropriate synthetic route and substrates, it is possible to introduce the desired functional groups at positions 7–10 on fluoranthenes.

## Introduction

**Figure 1:**
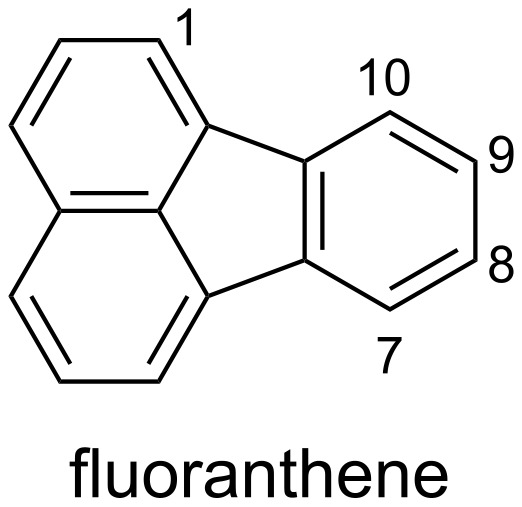
Structure of fluoranthene.

Substituted polyaromatics such as fluoranthenes (Figure 1) are widely used in materials chemistry due to their physical properties, and the introduction of a suitable functional group on the appropriate positions of fluoranthenes either allows their photophysical properties to be tuned, or enables them to be linked to other useful units [1]. There are several synthetic routes to fluoranthene derivatives, but in most cases several steps are required [2–5].

**Scheme 1:**
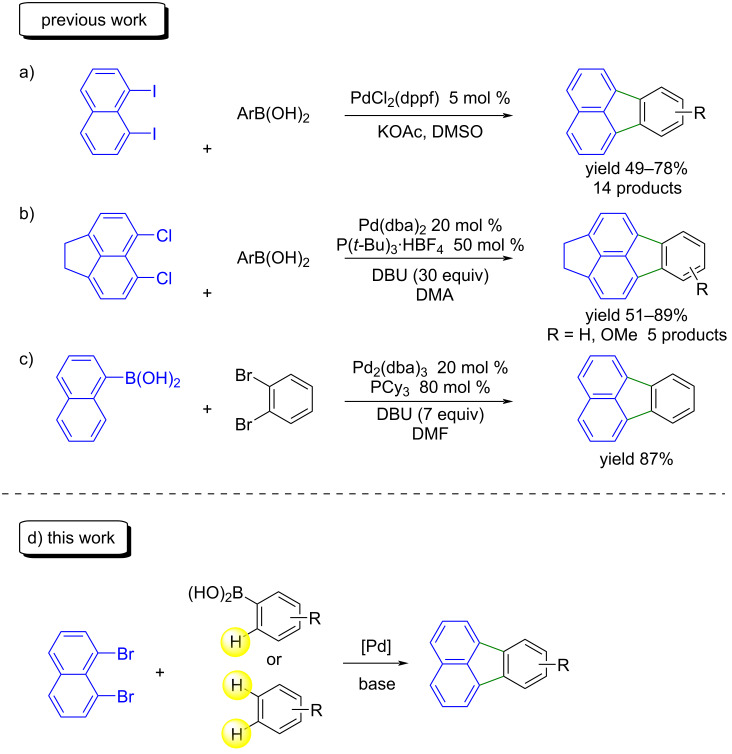
Pd-catalyzed access to fluoranthenes.

Over the past two decades, the Pd-catalyzed C–H arylation of a wide variety of arenes has led to a revolution in the synthesis of polyaromatic compounds [6–12]. Since the seminal results of Fagnou et al. in 2006 on the Pd-catalyzed C–H arylation of polyfluorobenzenes [13–16], several groups have described conditions enabling the direct intermolecular Pd-catalyzed arylation of arenes [17]. In contrast, only a few examples of fluoranthene backbone preparation by Pd-catalyzed C–H arylation have been reported. Some examples of the preparation of this skeleton by Suzuki coupling followed by intramolecular C–H coupling have been described [18–24]. In 2017, Metin, Türkmen and co-worker described the reaction of 1,8-diiodonaphthalene with arylboronic acids using PdCl_2_(dppf) as catalyst for the synthesis of various substituted fluoranthenes (Scheme 1a) [19]. In 2009, Quimby and Scott reported the use of 5,6-dichloro-1,2-dihydroacenaphthylene for the preparation of fluoranthene derivatives (Scheme 1b) [21]. In the course of this reaction 20 mol % of Pd catalyst, 50 mol % of phosphine ligand and 30 equiv of DBU as base were used to afford the desired fluoranthene derivatives. 1-Naphthylboronic acid and 1,2-dibromobenzene in the presence of Pd_2_(dba)_3_ (20 mol %) and PCy_3_ (80 mol %) using again a large excess of DBU base (7 equiv) also allowed to prepare unsubstituted fluoranthene in 87% yield (Scheme 1c) [22]. The reaction of naphthol with aryl bromides followed by nonaflation and intramolecular C–H activation for the access to fluoranthenes has also been reported [23]. Most of these reactions require high catalyst or base loadings, and offer a very limited scope regarding the use of reagents featuring functional groups useful in organic synthesis. Consequently, the discovery of simpler and more efficient synthetic procedures for the preparation of fluoranthene derivatives using commercially available substrates and allowing the regioselective introduction of functional groups at the desired positions is still needed. By using Pd-catalyzed double C–H bond functionalization of activated arenes, such as (poly)fluoroarenes, or by combining a palladium-catalyzed intermolecular Suzuki coupling with an intramolecular C–H arylation, it should be possible to access numerous fluoranthene derivatives from commercially available 1,8-dibromobenzene in a single manipulation (Scheme 1d).

Here, we describe i) conditions enabling the annulative π-extension of 1,8-dibromonaphthalene by successive palladium-catalyzed intermolecular and intramolecular C–H arylations using arenes activated by fluorine or chlorine substituents, or by combining a palladium-catalyzed intermolecular Suzuki coupling followed by an intramolecular C–H bond arylation, ii) on functional group tolerance for both synthetic routes for the introduction of substituents at the desired positions.

## Results and Discussion

For our study, we selected 1,2,3,4-tetrafluorobenzene (1.5 equiv) and 1,8-dibromonaphthalene (1 equiv) as model substrates (Table 1). We first investigated the reaction outcome using a set of bases in the presence of Pd(OAc)_2_ as the catalyst (5 mol %) in DMA at 150 °C. This catalyst precursor is known to efficiently promote the direct coupling of 5-membered ring heteroarenes with aryl halides [25]. Cs_2_CO_3_ and K_2_CO_3_ proved to be totally inefficient bases, while acetate bases gave the desired product **1** but only in trace amounts (Table, entries 1–5). In contrast, the use of KOPiv gave fluoranthene **1** in good yield, with partial conversion of 1,8-dibromonaphthalene (Table 1, entry 6). In order to improve the conversion of 1,8-dibromonaphthalene, the influence of the presence of diphosphine ligands was examined. Slightly better yields of **1** were obtained using the diphosphine ligands dppe, dppb or dppf associated with Pd(OAc)_2_, and the preformed catalyst PdCl(C_3_H_5_)(dppb) gave **1** in 74% yield (Table 1, entries 7–10) [26]. The influence of a variety of solvents was also examined. DMF and NMP gave **1** in slightly lower yields than those obtained using DMA as solvent, while xylene was inefficient (Table 1, entries 11–13). Using a lower reaction temperature or lower catalyst loadings (1% and 2% instead of 5%) led to lower yields of **1** due to the partial conversion of 1,8-dibromonaphthalene (Table 1, entries 14–16). The decisive influence of the base for this reaction is probably due to a concerted metalation–deprotonation mechanism [27–30].

**Table 1:** Influence of the conditions on the Pd-catalyzed reaction of 1,8-dibromonaphthalene with 1,2,3,4-tetrafluorobenzene.^a^

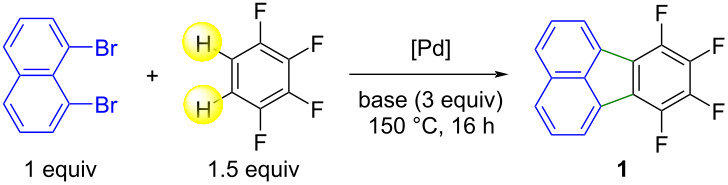				

Entry	Catalyst	Solvent	Base	Yield of **1** (%)

1	Pd(OAc)_2_	DMA	Cs_2_CO_3_	0
2	Pd(OAc)_2_	DMA	K_2_CO_3_	0
3	Pd(OAc)_2_	DMA	NaOAc	<3
4	Pd(OAc)_2_	DMA	CsOAc	<3
5	Pd(OAc)_2_	DMA	KOAc	10
6	Pd(OAc)_2_	DMA	KOPiv	45
7	Pd(OAc)_2_/dppe	DMA	KOPiv	51
8	Pd(OAc)_2_/dppb	DMA	KOPiv	55
9	Pd(OAc)_2_/dppf	DMA	KOPiv	52
10	PdCl(C_3_H_5_)(dppb)	DMA	KOPiv	74
11	PdCl(C_3_H_5_)(dppb)	NMP	KOPiv	62
12	PdCl(C_3_H_5_)(dppb)	DMF	KOPiv	56
13	PdCl(C_3_H_5_)(dppb)	xylene	KOPiv	<5
14	PdCl(C_3_H_5_)(dppb)	DMA	KOPiv	28^b^
15	PdCl(C_3_H_5_)(dppb)	DMA	KOPiv	50^c^
16	PdCl(C_3_H_5_)(dppb)	DMA	KOPiv	36^d^

^a^[Pd] (0.05 equiv), 1,8-dibromonaphthalene (1 equiv), 1,2,3,4-tetrafluorobenzene (1.5 equiv), base (3 equiv), 150 °C, isolated yields. ^b^130 °C. ^c^[Pd] (0.02 equiv). ^d^[Pd] (0.01 equiv).

**Scheme 2:**
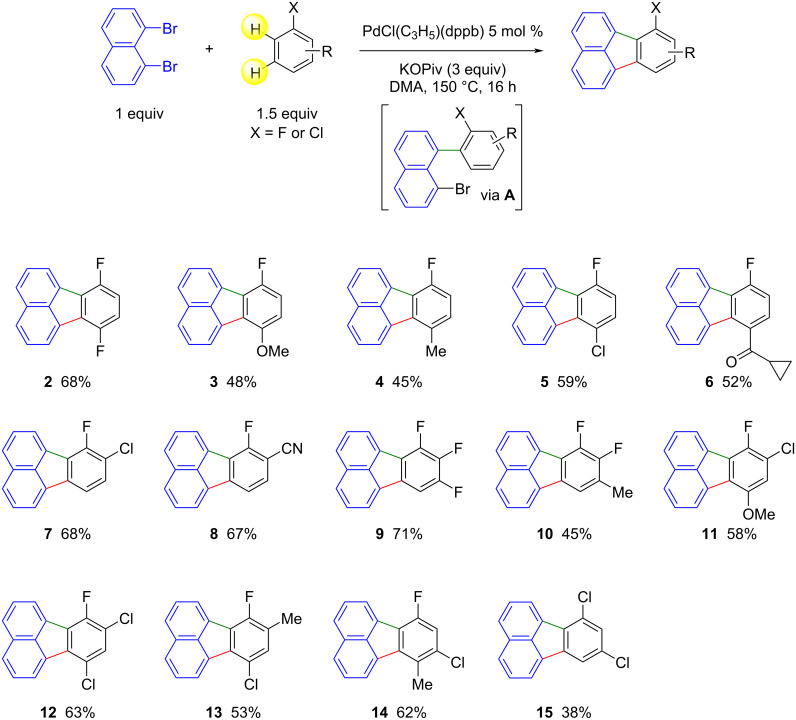
Scope of the Pd-catalyzed direct arylation reaction of arenes with 1,8-dibromonaphthalene.

Then, the scope of the Pd-catalyzed direct arylation for access to fluoranthenes was investigated (Scheme 2). The first step of the catalytic cycle involves the oxidative addition of 1,8-dibromonaphthalene. Then, a concerted metalation–deprotonation of the arene, which usually occurs at the *ortho*-position of an activating group such as a fluorine or a chlorine atom, followed by reductive elimination, gives the corresponding intermediate 1-aryl-8-bromonaphthalene **A** (Scheme 2). This catalytic cycle is followed by a second involving oxidative addition of 1-aryl-8-bromonaphthalene **A**, followed by intramolecular C–H coupling of the two aryl rings to give the fluoranthene derivative. Consequently, the use of arenes bearing substituents at the appropriate positions should enable them to be introduced regioselectively at the desired fluoranthene position, due to the greater reactivity of some of the C–H bonds in Pd-catalyzed direct arylations. First, we used 1,4-difluorobenzene under optimized reaction conditions, – i.e., 5 mol % PdCl(C_3_H_5_)(dppb) catalyst with KOPiv base in DMA at 150 °C – and the expected product **2** was obtained in 68% yield. Fluorobenzenes bearing methoxy, methyl, chloro or cyclopropanecarbonyl substituents in the *para*-position also gave the desired fluoranthenes **3**–**6** in moderate yields. The Pd-catalyzed direct arylation of 1-chloro-2-fluorobenzene is known to take place at the C–H bond *ortho* to the fluorine atom [31]. Consequently, from 1,8-dibromonaphthalene and 1-chloro-2-fluorobenzene, fluoranthene **7** bearing a fluorine substituent at position 7 and a chlorine at position 8 was obtained. 7-Fluorofluoranthene-8-carbonitrile (**8**) has also been successfully prepared from 2-fluorobenzonitrile. The first arylation between 1,2,3-trifluorobenzene and 1,8-dibromonaphthalene also takes place *ortho* to one of the fluorine atoms to give compound **9** in 71% yield. The use of 1,2-difluoro-3-methylbenzene allowed us to obtain 7,8-difluoro-9-methylfluoranthene (**10**) with a yield of 45%. Four fluorobenzenes bearing chloro, methyl or methoxy substituents at two positions were then employed. In all cases, the desired fluoranthenes **11**–**14** bearing substituents at positions 7–10 were obtained in moderate yields. With 1,3-dichlorobenzene, carbon 2 should be more reactive than carbon 4 in the Pd-catalyzed direct arylation, but we previously observed that the main product of the reaction was C4-arylated 1,3-dichlorobenzene [32]. This selectivity could be due to the steric hindrance of the chloro substituents. As a result, in the presence of 1,8-dibromonaphthalene, 1,3-dichlorobenzene led to 7,9-dichlorofluoranthene (**15**) with a yield of 38%.

**Scheme 3:**
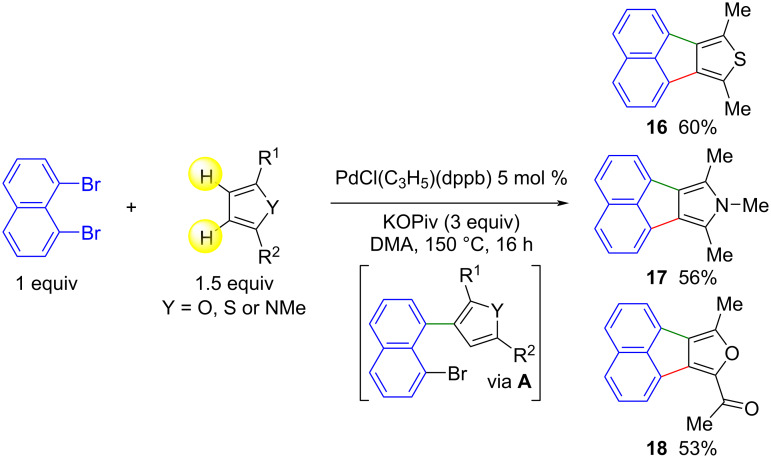
Scope of the Pd-catalyzed direct arylation reaction of 2,5-substituted heteroarenes with 1,8-dibromonaphthalene.

We have previously described the reaction of 1,8-dibromonaphthalene with some heteroarenes such as 2-methylthiophene in the presence of a palladium catalyst for the synthesis of acenaphtho[1,2-*d*]thiophenes [25]. In these reactions, the C–H bonds at the C2 and C3 positions of the thienyl unit were functionalized. In order to obtain acenaphtho[1,2-*c*]thiophenes and related compounds that are not easily accessible [33–38], we investigated the reactivity of three 5-membered heteroarenes substituted at positions 2 and 5 with 1,8-dibromonaphthalene using 5 mol % PdCl(C_3_H_5_)(dppb) catalyst with KOPiv as the base in DMA (Scheme 3). From 2,5-dimethylthiophene or 1,2,5-trimethylpyrrole, the desired acenaphtho[1,2-*c*]thiophene **16** and acenaphtho[1,2-*c*]pyrrole and **17** were obtained in 60% and 56% yield, respectively. The unsymmetrically substituted 1-(5-methylfuran-2-yl)ethan-1-one also reacted satisfactorily with 1,8-dibromonaphthalene to give acenaphtho[1,2-*c*]furan **18** in 53% yield. It is worth mentioning that at the present time, very few methods for the preparation of acenaphtho[1,2-*c*]furans have been reported [39–40].

**Scheme 4:**
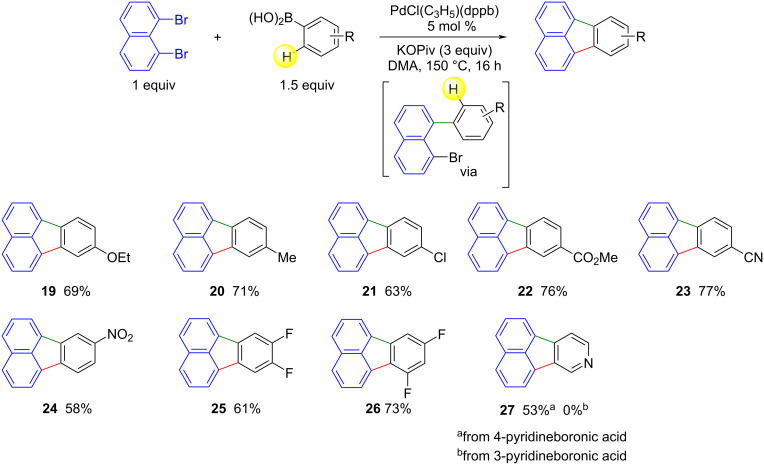
Scope of the Pd-catalyzed Suzuki reaction followed by direct arylation of arylboronic acids with 1,8-dibromonaphthalene.

The synthesis of fluoranthenes from 1,8-dibromonaphthalene via a double C–H bond activation of the arene used as coupling partner remains limited to specific arenes featuring an activated C–H bond. Consequently, we also investigated the access to fluoranthenes from 1,8-dibromonaphthalene via Suzuki coupling with arylboronic acids (Scheme 4). Using the same reaction conditions as for the double activation of C–H bonds described in Scheme 2, the reaction of 1,8-dibromonaphthalene with 4-ethoxybenzeneboronic acid gave the desired 8-ethoxyfluoranthene (**19**) in 69% yield. The influence of a range of *para*-substituents on the aryl unit of the arylboronic acids was then evaluated (Scheme 4). Methyl, ester and nitrile substituents were well tolerated, giving rise to products **20**, **22**, and **23** in yields ranging from 71% to 77%. A 4-chloro substituent was also well tolerated, providing product **21** in 63% yield without cleavage of the C–Cl bond. The use of 3-substituted arylboronic acids is also possible. The reaction of 3-nitrophenylboronic acid gave product **24** in 58% yield. Access to products **25** and **26** by activating two C–H bonds is not possible due to the higher reactivity of the C–H bond adjacent or between the two fluorine atoms in these arenes. The synthesis of these two fluoranthene derivatives by Suzuki coupling followed by intramolecular C–H activation was therefore investigated. From (3,4-difluorophenyl)boronic acid and (3,5-difluorophenyl)boronic acid, target products **25** and **26** were obtained in 61% and 73% yield, respectively. Finally, the reactivity of pyridinylboronic acids for the preparation of acenaphtho[1,2-*c*]pyridine (**27**) was evaluated. The target product **27** was obtained in 53% yield from pyridin-4-ylboronic acid, while no product **27** was obtained from pyridin-3-ylboronic acid.

**Scheme 5:**
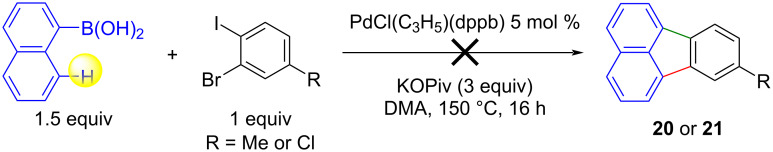
Attempted reaction of 1-naphthylboronic acid with 1,2-dihalobenzenes.

In contrast, under the same reaction conditions, the use of 1-naphthylboronic acid with 3-bromo-4-iodotoluene or 2-bromo-4-chloroiodobenzene failed to yield the desired products **20** and **21** (Scheme 5). In both cases, the formation of several unidentified side products was observed and a very large quantity of 1,2-dihalobenzenes was recovered unreacted.

**Scheme 6:**
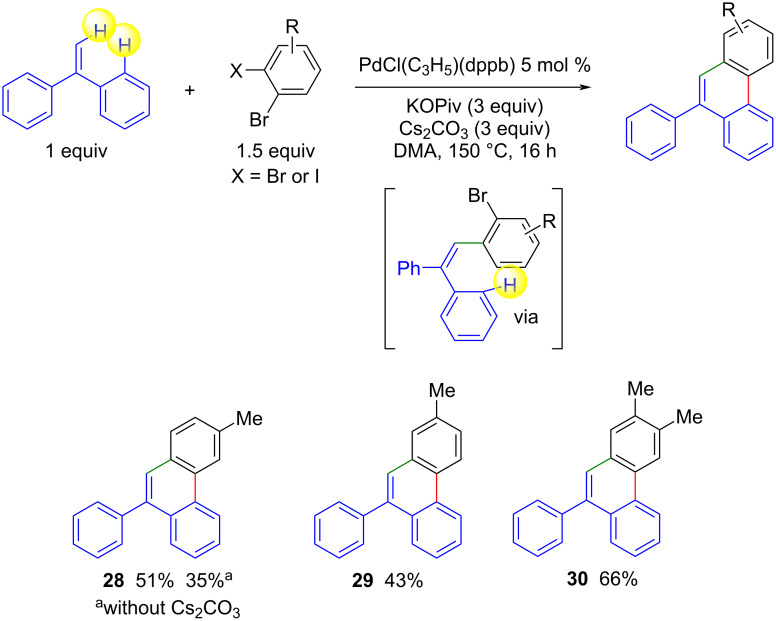
Pd-catalyzed Heck reaction followed by direct arylation of 1,1-diphenylethylene with 1,2-dihalobenzenes.

As our reaction conditions are effective in promoting the Pd-catalyzed intermolecular reaction followed by direct intramolecular C–H arylation, we have also applied them to the synthesis of 9-arylphenanthrenes by reaction of ethene-1,1-diyldibenzene with 1,2-dihalobenzenes. To our knowledge, this reaction has not yet been described, but 9-arylphenanthrenes can be synthesized by reacting 1-bromo-2-(1-phenylthenyl)benzene with bromobenzenes as coupling partners in the presence of a palladium catalyst [41]. We have observed that the reaction of 3-bromo-4-iodotoluene with 1,1-diphenylethylene yields the desired 9-arylphenanthrene **28** via a Heck reaction followed by intramolecular C–H arylation (Scheme 6). However, partial conversion of the starting material was observed. The use of 3 equiv KOPiv and 3 equiv Cs_2_CO_3_ as a base mixture increased the yield of product **28** to 51%. Under these conditions, 4-bromo-3-iodotoluene and 1,2-dibromo-4,5-dimethylbenzene also gave the desired products **29** and **30** in 43% and 66% yield, respectively.

## Conclusion

In summary, we report here complementary methods for the preparation of substituted fluoranthenes. The simplest involves 1,8-dibromonaphthalene with a double C–H bond functionalization of benzene derivatives. This method, which employs Pd-catalyzed intermolecular and then intramolecular activation of arene C–H bonds, tolerates several useful substituents on the arene, such as fluoro, chloro, methoxy, carbonyl or nitrile. Using the appropriate arenes, this synthetic route enables the desired functional groups to be introduced at positions 7 to 10 of fluoranthenes. The second method involves a Suzuki coupling followed by an intramolecular C–H bond activation step, and provides access to specific fluoranthenes that cannot be obtained by the double C–H bond functionalization method. Both procedures employ commercially available 1,8-dibromonaphthalene, arenes and arylboronic acids with an air-stable catalyst combined with KOPiv as an inexpensive base. These two complementary synthetic schemes, which employ very simple reaction conditions, should be of interest to chemists looking for quick pathways to functionalized fluoranthenes.

## Experimental

### General

Allylpalladium chloride dimer (98%) and 1,4-bis(diphenylphosphino)butane (dppb) (98%) were purchased from Aldrich. DMA (99+%) extra pure was purchased from Acros. KOPiv (95%) was purchased from Doug Discovery. These compounds were not purified before use. All reagents were weighed and handled in air. All reactions were carried out under an inert atmosphere with standard Schlenk techniques. ^1^H, ^19^F and ^13^C NMR spectra were recorded on Bruker Avance III 400 MHz and Bruker Avance neo 500 MHz spectrometers. High-resolution mass spectra were measured on a Bruker MaXis 4G spectrometer. Melting points were determined with a Kofler hot bench system. Chromatography purifications were performed using a CombiFlash NextGen 300 with Buchi FlashPure cartridges containing 40 μm irregular silica.

### General procedures

#### General procedure for the palladium-catalyzed synthesis of fluoranthenes **1**–**18** via double C–H bond functionalization

The reaction of 1,8-dibromonaphthalene (0.286 g, 1 mmol), (hetero)arene (1.5 mmol) and KOPiv (0.420 g, 3 mmol) in the presence of PdCl(C_3_H_5_)(dppb) (30.5 mg, 0.05 mmol) at 150 °C during 16 h in DMA (4 mL) under argon affords the coupling products **1**–**18** after evaporation of the solvent and purification on silica gel. Eluent EtOAc/heptane 1:9 for compounds **3**–**8**, **10**–**18**; heptane for compounds **1**, **2**, and **9**.

#### General procedure for the palladium-catalyzed synthesis of fluoranthenes **19**–**27** via Suzuki coupling followed by C–H bond functionalization

The reaction of 1,8-dibromonaphthalene (0.286 g, 1 mmol), arylboronic acid (1.5 mmol) and KOPiv (0.420 g, 3 mmol) in the presence of PdCl(C_3_H_5_)(dppb) (30.5 mg, 0.05 mmol) at 150 °C during 16 h in DMA (4 mL) under argon affords the coupling products **19**–**27** after evaporation of the solvent and purification on silica gel. Eluent EtOAc/heptane 1:9 for compounds **19**–**24**; heptane for compounds **25** and **26**; EtOAc/heptane 2:8 for compound **27**.

#### General procedure for the palladium-catalyzed synthesis of 9-arylphenanthrenes **28**–**30** via Heck reaction followed by intramolecular C–H bond arylation

The reaction of the 1,1-diphenylethylene (0.180 g, 1 mmol), 1,2-dihalobenzene (1.5 mmol), KOPiv (0.420 g, 3 mmol) and Cs_2_CO_3_ (0.978 g, 3 mmol) in the presence of PdCl(C_3_H_5_)(dppb) (30.5 mg, 0.05 mmol) at 150 °C during 16 h in DMA (4 mL) under argon affords the coupling products **28**–**30** after evaporation of the solvent and purification on silica gel. Eluent EtOAc/heptane 1:9 for all compounds.

## Supporting Information

File 1 Characterization data of synthesized compounds.

File 2 Copies of ^1^H, ^19^F, and ^13^C NMR spectra.

## Data Availability

All data that supports the findings of this study is available in the published article and/or the supporting information to this article.
