# Supplementary material for: Mono or double Pd-catalyzed C–H bond functionalization for the annulative π-extension of 1,8-dibromonaphthalene: a one pot access to fluoranthene derivatives
Source: Beilstein J Org Chem. 2024 Feb 23;20:427–35. doi: 10.3762/bjoc.20.37 (PMC10896227; doi:10.3762/bjoc.20.37)
Supplement: File 1 — Characterization data of synthesized compounds. [file Beilstein_J_Org_Chem-20-427-s001.pdf]

BEILSTEIN JOURNAL OF ORGANIC CHEMISTRY

Supporting Information

for

Mono or double Pd-catalyzed C–H bond functionalization for the annulative π-extension of 1,8-dibromonaphthalene: a one pot access to fluoranthene derivatives

Nahed Ketata, Linhao Liu, Ridha Ben Salem and Henri Doucet

Beilstein J. Org. Chem. **2024,** *20,* 427–435. doi:10.3762/bjoc.20.37

Characterization data of synthesized compounds

License and Terms: This is a supporting information file under the terms of the Creative Commons Attribution License (https://creativecommons.org/licenses/by/4.0). Please note that the reuse, redistribution and reproduction in particular requires that the author(s) and source are credited and that individual graphics may be subject to special legal provisions.
The license is subject to the *Beilstein Journal of Organic Chemistry* terms and conditions: (https://www.beilstein-journals.org/bjoc/terms)

7,8,9,10-Tetrafluorofluoranthene (1)

From 1,8-dibromonaphthalene (0.286 g, 1 mmol) and 1,2,3,4-tetrafluorobenzene (0.225 g, 1.5 mmol), **1** was isolated in 74% (0.203 g) yield as a yellow solid: mp 207-209 °C.

^{1}H NMR (500 MHz, $CDCl_3$): δ 8.12 (dd, *J* = 7.0, 0.6 Hz, 1H), 7.96 (dd, *J* = 8.3, 0.7 Hz, 1H), 7.72 (dd, *J* = 8.2, 7.0 Hz, 1H).

^{19}F NMR (376 MHz, $CDCl_3$): δ -143.7, -157.1.

^{13}C NMR (125 MHz, $CDCl_3$): δ 143.7 (d, *J* = 249.5 Hz), 132.4, 131.7, 129.8, 128.4, 127.8, 124.6, 21.8 (m).

HRMS calcd for $[M]^+$ $C_{16}H_6F_4$ 274.0400, found: 274.0399.

7,10-Difluorofluoranthene (2)

From 1,8-dibromonaphthalene (0.286 g, 1 mmol) and 1,4-difluorobenzene (0.171 g, 1.5 mmol), **2** was isolated in 68% (0.162 g) yield as a white solid: mp 139-141 °C.

^{1}H NMR (400 MHz, $CDCl_3$): δ 8.13 (d, *J* = 7.0 Hz, 2H), 7.93 (d, *J* = 8.3 Hz, 2H), 7.70 (dd, *J* = 8.3, 7.0 Hz, 2H), 7.05 (t, *J* = 6.0 Hz, 2H).

^{19}F NMR (376 MHz, $CDCl_3$): δ -124.1.

^{13}C NMR (100 MHz, $CDCl_3$): δ 155.1 (dd, *J* = 248.0, 3.8 Hz), 133.4, 131.5, 129.8, 128.2, 127.4, 127.3 (dd, *J* = 14.2, 11.3 Hz), 124.2 (t, *J* = 2.5 Hz), 115.7 (dd, *J* = 17.5, 14.0 Hz).

HRMS calcd for $[M+H]^+$ $C_{16}H_9F_2$ 239.0669, found: 239.0665.

7-Fluoro-10-methoxyfluoranthene (3)

From 1,8-dibromonaphthalene (0.286 g, 1 mmol) and 4-fluoroanisole (0.189 g, 1.5 mmol), **3** was isolated in 48% (0.120 g) yield as a yellow solid: mp 149-151 °C.

^{1}H NMR (400 MHz, $CDCl_3$): δ 8.21 (d, *J* = 6.9 Hz, 1H), 8.12 (d, *J* = 7.0 Hz, 1H), 7.90 (d, *J* = 8.2 Hz, 1H), 7.87 (d, *J* = 8.2 Hz, 1H), 7.70-7.64 (m, 2H), 7.06 (t, *J* = 8.9 Hz, 1H), 6.89 (dd, *J* = 8.9, 3.5 Hz, 1H), 4.08 (s, 3H).

^{19}F NMR (376 MHz, $CDCl_3$): δ -128.4.

^{13}C NMR (100 MHz, $CDCl_3$): δ 153.9 (d, *J* = 237.6 Hz), 135.5 (d, *J* = 2.1 Hz), 133.9 (d, *J* = 2.2 Hz), 131.4, 129.6, 128.2, 127.9, 127.1, 126.5, 124.3, 123.8, 123.7, 115.1 (d, *J* = 22.3 Hz), 111.2 (d, *J* = 7.5 Hz), 56.0.

HRMS calcd for $[M+H]^+$ $C_{17}H_{12}FO$ 251.0867, found: 251.0868.

7-Fluoro-10-methylfluoranthene (4)

From 1,8-dibromonaphthalene (0.286 g, 1 mmol) and 4-fluorotoluene (0.165 g, 1.5 mmol), **4** was isolated in 45% (0.105 g) yield as a yellow solid: mp 79-81 °C.

^{1}H NMR (400 MHz, $CDCl_3$): δ 8.13 (d, *J* = 6.9 Hz, 1H), 8.06 (d, *J* = 7.0 Hz, 1H), 7.93-7.88 (m, 2H), 7.72-7.67 (m, 2H), 7.15 (dd, *J* = 8.3, 4.9 Hz, 1H), 7.02 (t, *J* = 9.1 Hz, 1H), 2.77 (s, 3H).

^{19}F NMR (376 MHz, $CDCl_3$): δ -122.7.

^{13}C NMR (100 MHz, $CDCl_3$): δ 157.7 (d, *J* = 237.3 Hz), 137.3 (d, *J* = 2.1 Hz), 133.9 (d, *J* = 2.0 Hz), 132.0, 131.0, 130.9, 130.1, 129.8, 128.2, 127.9, 126.8, 126.7, 123.6, 123.5 (d, *J* = 4.3 Hz), 114.4 (d, *J* = 20.1 Hz), 19.8.

HRMS calcd for $[M+H]^+$ $C_{17}H_{12}F$ 235.0918, found: 235.0919.

7-Chloro-10-fluorofluoranthene (5)

From 1,8-dibromonaphthalene (0.286 g, 1 mmol) and 1-chloro-4-fluorobenzene (0.195 g, 1.5 mmol), **5** was isolated in 59% (0.150 g) yield as a yellow solid: mp 180-182 °C.

^{1}H NMR (400 MHz, $CDCl_3$): δ 8.53 (d, *J* = 7.1 Hz, 1H), 8.16 (d, *J* = 7.0 Hz, 1H), 7.98 (d, *J* = 7.6 Hz, 1H), 7.96 (d, *J* = 7.6 Hz, 1H), 7.75 (t, *J* = 7.0 Hz, 1H), 7.72 (t, *J* = 7.0 Hz, 1H), 7.33 (dd, *J* = 8.7, 4.3 Hz, 1H), 7.08 (t, *J* = 8.8 Hz, 1H).

^{19}F NMR (376 MHz, $CDCl_3$): δ -121.1.

^{13}C NMR (100 MHz, $CDCl_3$): δ 157.6 (d, *J* = 249.8 Hz), 138.0 (d, *J* = 6.5 Hz), 135.1, 132.7, 131.7, 129.7, 129.5 (d, *J* = 17.7 Hz), 128.2, 128.1, 127.9, 127.5, 127.3 (d, *J* = 17.7 Hz), 125.2 (d, *J* = 2.7 Hz), 124.8, 124.1 (d, *J* = 4.5 Hz), 115.7 (d, *J* = 22.2 Hz).

HRMS calcd for $[M+H]^+$ $C_{16}H_9ClF$ 255.0371, found: 255.0370.

Cyclopropyl(10-fluorofluoranthen-7-yl)methanone (6)

From 1,8-dibromonaphthalene (0.286 g, 1 mmol) and cyclopropyl(4-fluorophenyl)methanone (0.246 g, 1.5 mmol), **6** was isolated in 52% (0.150 g) yield as a yellow solid: mp 125-127 °C.

^{1}H NMR (400 MHz, $CDCl_3$): δ 8.50 (d, *J* = 7.2 Hz, 1H), 8.19 (d, *J* = 7.0 Hz, 1H), 7.96 (d, *J* = 7.1 Hz, 1H), 7.95 (d, *J* = 8.2 Hz, 1H), 7.84 (dd, *J* = 8.4, 5.0 Hz, 1H), 7.73-7.65 (m, 2H), 7.20 (t, *J* = 8.8 Hz, 1H), 2.70-2.61 (m, 1H), 1.50-1.42 (m, 2H), 1.23-1.13 (m, 2H).

^{19}F NMR (376 MHz, $CDCl_3$): δ -114.3.

^{13}C NMR (100 MHz, $CDCl_3$): δ 203.2, 160.5 (d, *J* = 254.7 Hz), 139.8 (d, *J* = 7.3 Hz), 135.0 (d, *J* = 2.2 Hz), 133.0 (d, *J* = 3.0 Hz), 132.5 (d, *J* = 1.9 Hz), 132.2, 129.7, 129.6, 129.5, 128.4, 128.2, 127.9, 127.6, 126.7, 123.9 (d, *J* = 4.8 Hz), 114.2 (d, *J* = 20.8 Hz), 20.7, 12.6.

HRMS calcd for $[M+H]^+$ $C_{20}H_{14}FO$ 289.1023, found: 289.1026.

8-Chloro-7-fluorofluoranthene (7)

From 1,8-dibromonaphthalene (0.286 g, 1 mmol) and 1-chloro-2-fluorobenzene (0.195 g, 1.5 mmol), **7** was isolated in 68% (0.173 g) yield as a yellow solid: mp 107-109 °C.

^1H NMR (400 MHz, $CDCl_3$): δ 8.14 (d, *J* = 6.9 Hz, 1H), 7.98 (d, *J* = 7.0 Hz, 1H), 7.93 (dd, *J* = 8.2, 2.0 Hz, 2H), 7.73-7.64 (m, 3H), 7.41 (t, *J* = 7.9 Hz, 1H).

^{19}F NMR (376 MHz, $CDCl_3$): δ -121.1.

^{13}C NMR (100 MHz, $CDCl_3$): δ 154.4 (d, *J* = 253.0 Hz), 141.4 (d, *J* = 6.0 Hz), 135.6 (d, *J* = 2.0 Hz), 133.1 (d, *J* = 2.1 Hz), 132.1, 129.9, 129.2, 128.4, 128.0, 127.5, 126.9 (d, *J* = 15.8 Hz), 124.1 (d, *J* = 4.2 Hz), 121.2, 120.0 (d, *J* = 16.9 Hz), 117.8 (d, *J* = 3.6 Hz).

HRMS calcd for $[M+H]^+$ $C_{16}H_9ClF$ 255.0371, found: 255.0370.

7-Fluorofluoranthene-8-carbonitrile (8)

From 1,8-dibromonaphthalene (0.286 g, 1 mmol) and 2-fluorobenzonitrile (0.182 g, 1.5 mmol), **8** was isolated in 67% (0.164 g) yield as a yellow solid: mp 148-150 °C.

^1H NMR (400 MHz, $CDCl_3$): δ 8.18 (d, *J* = 6.9 Hz, 1H), 8.10 (d, *J* = 7.0 Hz, 1H), 8.03 (d, *J* = 8.2 Hz, 1H), 8.00 (d, *J* = 8.2 Hz, 1H), 7.80 (d, *J* = 7.7 Hz, 1H), 7.78-7.73 (m, 2H), 7.64 (dd, *J* = 7.7, 5.8 Hz, 1H).

^{19}F NMR (376 MHz, $CDCl_3$): δ -113.2.

^{13}C NMR (100 MHz, $CDCl_3$): δ 160.0, 159.0 (d, *J* = 252.5 Hz), 146.3, 134.7, 132.5, 132.1, 131.9 (d, *J* = 2.0 Hz), 129.9, 129.2, 128.7, 128.2, 128.1, 124.8 (d, *J* = 3.7 Hz), 122.8, 117.9 (d, *J* = 3.3 Hz), 114.7, 99.7 (d, *J* = 15.1 Hz).

HRMS calcd for $[M+H]^+$ $C_{17}H_9FN$ 246.0714, found: 246.0714.

7,8,9-Trifluorofluoranthene (9)

From 1,8-dibromonaphthalene (0.286 g, 1 mmol) and 1,2,3-trifluorobenzene (0.132 g, 1.5 mmol), **9** was isolated in 71% (0.182 g) yield as a yellow solid: mp 145-147 °C.

^{1}H NMR (500 MHz, $CDCl_3$): δ 8.10 (d, *J* = 6.9 Hz, 1H), 7.98-7.90 (m, 3H), 7.73-7.67 (m, 2H), 7.54 (ddd, *J* = 9.2, 6.0, 1.5 Hz, 1H).

^{19}F NMR (376 MHz, $CDCl_3$): δ -135.2, -139.6, -162.0.

^{13}C NMR (125 MHz, $CDCl_3$): δ 151.1 (dm, *J* = 248.8 Hz), 147.5 (dm, *J* = 258.7 Hz), 139.6 (dm, *J* = 269.8 Hz), 143.4, 135.1, 135.0, 132.6, 132.3, 129.9, 128.5, 128.1, 127.7, 127.3, 123.7 (d, *J* = 4.0 Hz), 122.8 (dd, *J* = 10.0, 2.8 Hz), 121.2, 105.9 (dd, *J* = 19.7, 3.2 Hz).

HRMS calcd for $[M]^+$ $C_{16}H_7F_3$ 256.0494, found: 256.0495.

7,8-Difluoro-9-methylfluoranthene (10)

From 1,8-dibromonaphthalene (0.286 g, 1 mmol) and 1,2-difluoro-3-methylbenzene (0.192 g, 1.5 mmol), **10** was isolated in 45% (0.113 g) yield as a yellow solid: mp 111-113 °C.

^{1}H NMR (400 MHz, $CDCl_3$): δ 8.07 (d, *J* = 6.9 Hz, 1H), 7.93-7.84 (m, 3H), 7.68 (t, *J* = 87.8 Hz, 1H), 7.65 (t, *J* = 87.8 Hz, 1H), 7.50 (d, *J* = 3.4 Hz, 1H), 2.46 (s, 3H).

^{19}F NMR (376 MHz, $CDCl_3$): δ -143.9 (bs).

^{13}C NMR (100 MHz, $CDCl_3$): δ 136.0, 135.7 (m), 133.6 (d, *J* = 1.5 Hz), 132.4, 129.9, 128.3, 128.0, 127.0, 126.7, 120.4, 118.8 (m), 15.0.

HRMS calcd for $[M+H]^+$ $C_{17}H_{11}F_2$ 253.0823, found: 253.0819.

8-Chloro-7-fluoro-10-methoxyfluoranthene (11)

From 1,8-dibromonaphthalene (0.286 g, 1 mmol) and 2-chloro-1-fluoro-4-methoxybenzene (0.240 g, 1.5 mmol), **11** was isolated in 58% (0.165 g) yield as a yellow solid: mp 148-150 °C.

^{1}H NMR (400 MHz, $CDCl_3$): δ 8.17 (d, *J* = 7.0 Hz, 1H), 8.12 (d, *J* = 7.0 Hz, 1H), 7.92 (d, *J* = 8.2 Hz, 1H), 7.87 (d, *J* = 8.2 Hz, 1H), 7.68 (t, *J* = 7.0 Hz, 1H), 7.66 (t, *J* = 7.0 Hz, 1H), 6.94 (t, *J* = 5.3 Hz, 1H), 4.06 (s, 3H).

^{19}F NMR (376 MHz, $CDCl_3$): δ -131.0.

^{13}C NMR (100 MHz, $CDCl_3$): δ 152.2, 149.4 (d, *J* = 245.8 Hz), 134.8 (m), 133.7 (d, *J* = 2.3 Hz), 131.4, 129.6, 128.3, 128.0, 127.6, 126.7, 124.5, 124.3 (d, *J* = 4.3 Hz), 119.9 (d, *J* = 18.5 Hz), 111.9, 56.2.

HRMS calcd for $[M+H]^+$ $C_{17}H_{11}ClFO$ 285.0477, found: 285.0477.

8,10-Dichloro-7-fluorofluoranthene (12)

From 1,8-dibromonaphthalene (0.286 g, 1 mmol) and 2,4-dichloro-1-fluorobenzene (0.248 g, 1.5 mmol), **12** was isolated in 63% (0.182 g) yield as a yellow solid: mp 169-171 °C.

.^{1}H NMR (400 MHz, $CDCl_3$): δ 8.47 (d, *J* = 7.1 Hz, 1H), 8.16 (d, *J* = 7.0 Hz, 1H), 7.98 (d, *J* = 8.2 Hz, 2H), 7.73 (t, *J* = 8.2 Hz, 2H), 7.41 (d, *J* = 5.9 Hz, 1H).

^{19}F NMR (376 MHz, $CDCl_3$): δ -123.6.

^{13}C NMR (100 MHz, $CDCl_3$): δ 153.2 (d, *J* =252.3 Hz), 136.5, 134.4, 132.2, 131.9, 129.7, 129.4, 127.3, 128.3 (m), 128.1 (d, *J* = 4.0 Hz), 125.0, 124.6 (d, *J* = 6.6 Hz), 120.4 (d, *J* = 18.5 Hz).

HRMS calcd for $[M+H]^+$ $C_{16}H_8Cl_2F$ 288.9982, found: 288.9980.

10-Chloro-7-fluoro-8-methylfluoranthene (13)

From 1,8-dibromonaphthalene (0.286 g, 1 mmol) and 4-chloro-1-fluoro-2-methylbenzene (0.218 g, 1.5 mmol), **13** was isolated in 53% (0.143 g) yield as a yellow solid: mp 115-117 °C.

^{1}H NMR (400 MHz, $CDCl_3$): δ 8.43 (d, *J* = 7.1 Hz, 1H), 8.13 (d, *J* = 6.9 Hz, 1H), 7.93 (d, *J* = 8.2 Hz, 2H), 7.73-7.68 (m, 2H), 7.18 (d, *J* = 6.0 Hz, 1H), 2.43 (s, 3H).

^{19}F NMR (376 MHz, $CDCl_3$): δ -125.4.

^{13}C NMR (100 MHz, $CDCl_3$): δ 156.2 (d, *J* = 248.6 Hz), 135.6, 135.2, 133.0, 131.9, 130.8 (d, *J* = 4.7 Hz), 129.7, 128.1 (d, *J* = 2.3 Hz), 127.3 (d, *J* = 2.4 Hz), 125.9 (d, *J* = 18.3 Hz), 124.5, 124.2, 123.9 (d, *J* = 4.8 Hz), 14.5.

HRMS calcd for $[M+H]^+$ $C_{17}H_{11}ClF$ 269.0528, found: 269.0528.

8-Chloro-10-fluoro-7-methylfluoranthene (14)

From 1,8-dibromonaphthalene (0.286 g, 1 mmol) and 2-chloro-4-fluoro-1-methylbenzene (0.216 g, 1.5 mmol), **14** was isolated in 62% (0.167 g) yield as a yellow solid: mp 144-146 °C.

^{1}H NMR (400 MHz, $CDCl_3$): δ 8.05 (d, *J* = 6.9 Hz, 1H), 8.02 (d, *J* = 6.9 Hz, 1H), 7.90 (d, *J* = 8.0 Hz, 1H), 7.87 (d, *J* = 8.0 Hz, 1H), 7.66 (t, *J* = 7.5 Hz, 2H), 7.15 (d, *J* = 9.1 Hz, 1H), 2.75 (s, 3H).

^{19}F NMR (376 MHz, $CDCl_3$): δ -120.8.

^{13}C NMR (100 MHz, $CDCl_3$): δ 156.4 (d, *J* = 250.5 Hz), 140.2 (d, *J* = 6.7 Hz), 136.6 (d, *J* = 2.6 Hz), 133.9 (d, *J* = 9.1 Hz), 132.9 (d, *J* = 2.2 Hz), 132.0, 129.8, 128.2 (m), 127.9, 127.4, 126.9, 124.4 (d, *J* = 16.2 Hz), 124.2, 115.4 (d, *J* = 31.1 Hz), 16.1.

HRMS calcd for $[M+H]^+$ $C_{17}H_{11}ClF$ 269.0528, found: 269.0526.

7,9-Dichlorofluoranthene (15)

From 1,8-dibromonaphthalene (0.286 g, 1 mmol) and 1,3-dichlorobenzene (0.221 g, 1.5 mmol), **15** was isolated in 38% (0.103 g) yield as a yellow solid: mp 139-141 °C.

^{1}H NMR (400 MHz, $CDCl_3$): δ 8.44 (d, *J* = 7.0 Hz, 1H), 7.99-7.93 (m, 3H), 7.82 (d, *J* = 1.8 Hz, 1H), 7.72 (t, *J* = 8.0 Hz, 1H), 7.69 (t, *J* = 8.0 Hz, 1H), 7.39 (d, *J* = 1.7 Hz, 1H).

^{13}C NMR (100 MHz, $CDCl_3$): δ 142.4, 134.8, 134.7, 134.6, 133.3, 132.4, 130.3, 129.8, 128.3, 128.1, 128.0, 127.9, 127.5, 124.4, 121.1, 120.3.

HRMS calcd for $[M+H]^+$ $C_{16}H_9Cl_2$ 271.0076, found: 271.0075.

7,9-Dimethylacenaphtho[1,2-*c*]thiophene (16)

From 1,8-dibromonaphthalene (0.286 g, 1 mmol) and 2,5-dimethylthiophene (0.168 g, 1.5 mmol), **16** was isolated in 60% (0.142 g) yield as a yellow solid: mp 111-113 °C.

^{1}H NMR (400 MHz, $CDCl_3$): δ 7.70 (d, *J* = 8.2 Hz, 2H), 7.64 (d, *J* = 6.9 Hz, 2H), 7.56 (dd, *J* = 8.2, 6.9 Hz, 2H), 2.68 (s, 6H).

^{13}C NMR (100 MHz, $CDCl_3$): δ 140.5, 140.0, 133.9, 131.3, 128.2, 127.7, 124.4, 118.4, 13.8.

HRMS calcd for $[M+H]^+$ $C_{16}H_{13}S$ 237.0732, found: 237.0729.

7,8,9-Trimethyl-8H-acenaphtho[1,2-*c*]pyrrole (17)

From 1,8-dibromonaphthalene (0.286 g, 1 mmol) and 1,2,5-trimethylpyrrole (0.164 g, 1.5 mmol), **18** was isolated in 56% (0.130 g) yield as a yellow solid: mp 193-195 °C.

^{1}H NMR (400 MHz, $CDCl_3$): δ 7.55-7.50 (m, 2H), 7.48-7.43 (m, 4H), 3.46 (s, 3H), 2.49 (s, 6H).

^{13}C NMR (100 MHz, $CDCl_3$): δ 137.6, 134.9, 131.0, 127.5, 123.9, 122.7, 122.1, 116.8, 27.0, 12.1.

HRMS calcd for $[M+H]^+$ $C_{17}H_{16}N$ 234.1272, found: 234.1275.

1-(9-Methylacenaphtho[1,2-*c*]furan-7-yl)ethan-1-one (18)

From 1,8-dibromonaphthalene (0.286 g, 1 mmol) and 1-(5-methylfuran-2-yl)ethan-1-one (0.186 g, 1.5 mmol), **17** was isolated in 53% (0.131 g) yield as a yellow solid: mp 147-149 °C.

^{1}H NMR (400 MHz, $CDCl_3$): δ 8.36 (d, *J* = 7.0 Hz, 1H), 7.90 (d, *J* = 8.2 Hz, 1H), 7.81 (d, *J* = 8.2 Hz, 1H), 7.71-7.67 (m, 2H), 7.61 (t, *J* = 7.1 Hz, 1H), 2.71 (s, 3H), 2.65 (s, 3H).

^{13}C NMR (100 MHz, $CDCl_3$): δ 187.1, 146.9, 143.2, 139.2, 136.9, 131.0, 130.4, 129.9, 128.3, 127.6, 125.5, 124.8, 120.0, 26.5, 14.1.

HRMS calcd for $[M+H]^+$ $C_{17}H_{13}O_2$ 249.0910, found: 249.0910.

8-Ethoxyfluoranthene (19)

From 1,8-dibromonaphthalene (0.286 g, 1 mmol) and (4-ethoxyphenyl)boronic acid (0.249 g, 1.5 mmol), **19** was isolated in 69% (0.170 g) yield as a yellow solid: mp 110-112 °C.

^{1}H NMR (400 MHz, $CDCl_3$): δ 7.92 (d, *J* = 6.8 Hz, 1H), 7.88-7.76 (m, 4H), 7.68-7.58 (m, 2H), 7.49 (d, *J* = 2.3 Hz, 1H), 6.93 (dd, *J* = 8.3, 2.3 Hz, 1H), 4.19 (q, *J* = 7.4 Hz, 2H), 1.53 (t, *J* = 7.4 Hz, 3H).

^{13}C NMR (100 MHz, $CDCl_3$): δ 159.3, 141.2, 137.1, 136.9, 132.9, 132.3, 130.0, 128.1, 127.8, 126.9, 125.4, 122.2, 119.9, 118.9, 113.4, 108.3, 63.8, 15.0.

HRMS calcd for $[M+H]^+$ $C_{18}H_{15}O$ 247.1117, found: 247.1118.

8-Methylfluoranthene (20) [1]

From 1,8-dibromonaphthalene (0.286 g, 1 mmol) and *p*-tolylboronic acid (0.204 g, 1.5 mmol), **20** was isolated in 71% (0.153 g) yield as a yellow solid: mp 77-79 °C.

^{1}H NMR (400 MHz, $CDCl_3$): δ 7.96-7.88 (m, 2H), 7.87-7.79 (m, 3H), 7.76 (s, 1H), 7.68-7.60 (m, 2H), 7.22 (d, *J* = 7.7 Hz, 1H), 2.52 (s, 3H).

^{13}C NMR (100 MHz, $CDCl_3$): δ 139.8, 137.5, 137.1, 137.0, 136.9, 132.6, 130.0, 128.3, 127.9, 127.8, 126.5, 126.1, 122.3, 121.2, 119.8, 119.5, 21.8.

HRMS calcd for $[M+H]^+$ $C_{17}H_{13}$ 217.1012, found: 217.1010.

8-Chlorofluoranthene (21) [2]

From 1,8-dibromonaphthalene (0.286 g, 1 mmol) and (4-chlorophenyl)boronic acid (0.234 g, 1.5 mmol), **21** was isolated in 63% (0.148 g) yield as a yellow solid: mp 108-110 °C.

^{1}H NMR (400 MHz, $CDCl_3$): δ 8.01-7.82 (m, 6H), 7.72-7.64 (m, 2H), 7.38 (dd, *J* = 8.0, 1.9 Hz, 1H).

^{13}C NMR (100 MHz, $CDCl_3$): δ 141.0, 137.7, 135.9, 135.8, 133.3, 132.5, 130.0, 128.1, 128.0, 127.4, 127.3, 126.9, 122.3, 121.9, 120.6, 120.3.

HRMS calcd for $[M+H]^+$ $C_{16}H_{10}Cl$ 237.0466, found: 237.0466.

Methyl fluoranthene-8-carboxylate (22) [3]

From 1,8-dibromonaphthalene (0.286 g, 1 mmol) and (4-(methoxycarbonyl)phenyl)boronic acid (0.270 g, 1.5 mmol), **22** was isolated in 76% (0.198 g) yield as a yellow solid: mp 89-91 °C.

^{1}H NMR (400 MHz, $CDCl_3$): δ 8.55 (d, *J* = 1.3 Hz, 1H), 8.09 (dd, *J* = 8.0, 1.5 Hz, 1H), 7.99 (d, *J* = 6.9 Hz, 1H), 7.97 (d, *J* = 6.8 Hz, 1H), 7.93-7.85 (m, 3H), 7.69-7.62 (m, 2H), 4.01 (s, 3H).

^{13}C NMR (100 MHz, $CDCl_3$): δ 167.4, 143.5, 139.4, 136.0, 135.8, 132.9, 130.0, 129.2, 129.0, 128.2, 128.0, 127.8, 127.1, 122.6, 121.3, 121.1, 120.7, 52.2.

HRMS calcd for $[M+H]^+$ $C_{18}H_{13}O_2$ 261.0910, found: 261.0907.

Fluoranthene-8-carbonitrile (23) [3]

From 1,8-dibromonaphthalene (0.286 g, 1 mmol) and (4-cyanophenyl)boronic acid (0.221 g, 1.5 mmol), **23** was isolated in 77% (0.175 g) yield as a yellow solid: mp 137-139 °C.

^{1}H NMR (400 MHz, $CDCl_3$): δ 7.97 (s, 1H), 7.95-7.88 (m, 3H), 7.85 (d, *J* = 7.8 Hz, 1H), 7.81 (dd, *J* = 7.8, 0.5 Hz, 1H), 7.70-7.61 (m, 2H), 7.56 (dd, *J* = 7.8, 1.4 Hz, 1H).

^{13}C NMR (100 MHz, $CDCl_3$): δ 142.9, 139.5, 134.9, 134.7, 132.5, 131.2, 129.9, 128.4, 128.2, 128.1, 127.8, 124.6, 121.8, 121.6, 121.2, 119.6, 110.3.

HRMS calcd for $[M+H]^+$ $C_{17}H_{10}N$ 228.0808, found: 228.0807.

8-Nitrofluoranthene (24) [3]

From 1,8-dibromonaphthalene (0.286 g, 1 mmol) and (3-nitrophenyl)boronic acid (0.251 g, 1.5 mmol), **24** was isolated in 58% (0.143 g) yield as a yellow solid: mp 164-166 °C.

^{1}H NMR (400 MHz, $CDCl_3$): δ 8.77 (d, *J* = 1.8 Hz, 1H), 8.33 (dd, *J* = 8.3, 2.1 Hz, 1H), 8.13 (d, *J* = 6.9 Hz, 1H), 8.11 (d, *J* = 6.9 Hz, 1H), 8.07-7.97 (m, 3H), 7.78 (d, *J* = 7.0 Hz, 1H), 7.75 (d, *J* = 7.0 Hz, 1H).

^{13}C NMR (100 MHz, $CDCl_3$): δ 144.9, 140.1, 135.7, 134.8, 134.6, 133.4, 130.1, 128.7, 128.4, 128.3, 128.1, 123.1, 122.4, 121.6, 121.3, 116.8.

HRMS calcd for $[M+H]^+$ $C_{16}H_{10}NO_2$ 248.0706, found: 248.0704.

8,9-Difluorofluoranthene (25)

From 1,8-dibromonaphthalene (0.286 g, 1 mmol) and (3,4-difluorophenyl)boronic acid (0.237 g, 1.5 mmol), **25** was isolated in 61% (0.145 g) yield as a yellow solid: mp 115-117 °C.

^{1}H NMR (400 MHz, $CDCl_3$): δ 7.94-7.85 (m, 4H), 7.74-7.62 (m, 4H).

^{19}F NMR (376 MHz, $CDCl_3$): δ -138.5.

^{13}C NMR (100 MHz, $CDCl_3$): δ 150.3 (dd, *J* = 249.0, 15.7, Hz), 135.6 (m), 135.4, 129.8, 128.1, 127.0, 120.4, 110.4 (dd, *J* = 13.2, 7.1 Hz).

HRMS calcd for $[M+H]^+$ $C_{16}H_9F_2$ 239.0667, found: 239.0667.

7,9-Difluorofluoranthene (26)

From 1,8-dibromonaphthalene (0.286 g, 1 mmol) and (3,5-difluorophenyl)boronic acid (0.237 g, 1.5 mmol), **26** was isolated in 73% (0.174 g) yield as a yellow solid: mp 83-85 °C.

^1H NMR (400 MHz, $CDCl_3$): δ 8.06 (d, *J* = 6.9 Hz, 1H), 7.97 (d, *J* = 7.0 Hz, 1H), 7.94 (d, *J* = 8.2 Hz, 1H), 7.88 (d, *J* = 8.3 Hz, 1H), 7.71-7.65 (m, 2H), 7.45 (dd, *J* = 8.1, 2.0 Hz, 1H), 6.87 (td, *J* = 9.5, 2.0 Hz, 1H).

^{19}F NMR (376 MHz, $CDCl_3$): δ -111.0, -114.9.

^{13}C NMR (100 MHz, $CDCl_3$): δ 135.7, 133.2, 132.3, 129.9, 128.4, 128.0, 127.8, 126.5, 123.2 (dd, *J* = 3.9, 1.5 Hz), 121.3, 105.3 (dd, *J* = 23.7, 3.4 Hz), 103.0 (d, *J* = 24.4 Hz), 102.7 (d, *J* = 24.4 Hz).

HRMS calcd for $[M]^+$ $C_{16}H_8F_2$ 238.0587, found: 238.0589.

Acenaphtho[1,2-*c*]pyridine (27) [3]

From 1,8-dibromonaphthalene (0.286 g, 1 mmol) and pyridin-4-ylboronic acid (0.185 g, 1.5 mmol), **27** was isolated in 53% (0.108 g) yield as a brown solid: mp 64-66 °C.

^1H NMR (400 MHz, $CDCl_3$): δ 9.16 (d, *J* = 0.9 Hz, 1H), 8.65 (d, *J* = 5.0 Hz, 1H), 8.06-7.95 (m, 3H), 7.90 (d, *J* = 8.0 Hz, 1H), 7.78 (dd, *J* = 5.0, 1.0 Hz, 1H), 7.72-7.63 (m, 2H).

^{13}C NMR (100 MHz, $CDCl_3$): δ 148.6, 146.1, 142.9, 134.6, 134.5, 134.1, 132.3, 130.1, 129.1, 128.3, 128.0, 127.4, 122.4, 121.3, 116.4.

HRMS calcd for $[M+H]^+$ $C_{15}H_{10}N$ 204.0808, found: 204.0807.

3-Methyl-9-phenylphenanthrene (28) [4]

From 1,1-diphenylethylene (0.180 g, 1 mmol) and 3-bromo-4-iodotoluene (0.445 g, 1.5 mmol), **28** was isolated in 51% (0.137 g) yield as a white solid: mp 88-90 °C.

^1H NMR (400 MHz, $CDCl_3$): δ 8.80 (d, *J* = 8.1 Hz, 1H), 8.55 (s, 1H), 7.94 (d, *J* = 8.1 Hz, 1H), 7.82 (d, *J* = 8.1 Hz, 1H), 7.70-7.65 (m, 2H), 7.62-7.45 (m, 7H), 2.68 (s, 3H).

^{13}C NMR (100 MHz, $CDCl_3$): δ 140.9, 137.8, 136.3, 131.2, 130.4, 130.1, 130.0, 129.5, 128.6, 128.5, 128.3, 127.4, 127.2, 126.9, 126.3, 126.2, 122.9, 122.3, 22.2.

HRMS calcd for $[M+H]^+$ $C_{21}H_{17}$ 269.1325, found: 269.1325.

2-Methyl-9-phenylphenanthrene (29) [4]

From 1,1-diphenylethylene (0.180 g, 1 mmol) and 4-bromo-3-iodotoluene (0.445 g, 1.5 mmol), **29** was isolated in 43% (0.115 g) yield as a white solid: mp 69-71 °C.

^{1}H NMR (400 MHz, $CDCl_3$): δ 8.77 (d, *J* = 8.1 Hz, 1H), 8.65 (d, *J* = 8.1 Hz, 1H), 7.93 (d, *J* = 8.1 Hz, 1H), 7.72-7.65 (m, 3H), 7.60-7.45 (m, 7H), 2.61 (s, 3H).

^{13}C NMR (100 MHz, $CDCl_3$): δ 140.9, 138.8, 136.6, 131.7, 130.8, 130.7, 130.1, 128.4, 128.3, 128.2, 127.9, 127.3, 126.9, 126.4, 126.1, 122.7, 122.5, 21.5.

HRMS calcd for $[M+H]^+$ $C_{21}H_{17}$ 269.1325, found: 269.1322.

2,3-Dimethyl-9-phenylphenanthrene (30)

From 1,1-diphenylethylene (0.180 g, 1 mmol) and 1,2-dibromo-4,5-dimethylbenzene (0.400 g, 1.5 mmol), **30** was isolated in 66% (0.186 g) yield as a white solid: mp 126-128 °C.

^{1}H NMR (400 MHz, $CDCl_3$): δ 8.78 (d, *J* = 8.1 Hz, 1H), 8.51 (s, 1H), 7.94 (d, *J* = 8.1 Hz, 1H), 7.70-7.46 (m, 9H), 2.60 (s, 3H), 2.52 (s, 3H).

^{13}C NMR (100 MHz, $CDCl_3$): δ 141.1, 137.9, 136.2, 135.9, 130.9, 130.4, 130.1, 128.7, 128.3, 128.2, 127.2, 127.1, 126.8, 126.2, 125.9, 122.9, 122.7, 20.7, 20.0.

HRMS calcd for $[M+H]^+$ $C_{22}H_{19}$ 283.1481, found: 283.1483.

References

1) Geary, L. M.; Chen, T.-Y.; Montgomery, T. P.; Krische, M. J. *J. Am. Chem. Soc.* **2014,** *136,* 5920–5922.
2) van Haeringen, C. J.; Lugtenburg, J.; Cornelisse, J. *Polycyclic Aromat. Compd.* **1996,** *8,* 9–22.
3) Pal, S.; Metin, O.; Turkmen, Y. E. *ACS Omega* **2017,** *2,* 8689–8696.
4) Wei, D.; Li, M.-Y.; Zhu, B.-B.; Yang, X.-D.; Zhang, F.; Feng, C.-G.; Lin, G.-Q. *Angew. Chem., Int. Ed.* **2019,** *58,* 16543–16547.
