# Supplementary material for: Mono or double Pd-catalyzed C–H bond functionalization for the annulative π-extension of 1,8-dibromonaphthalene: a one pot access to fluoranthene derivatives
Source: Beilstein J Org Chem. 2024 Feb 23;20:427–35. doi: 10.3762/bjoc.20.37 (PMC10896227; doi:10.3762/bjoc.20.37)

BEILSTEIN JOURNAL OF ORGANIC CHEMISTRY

Supporting Information

for

Mono or double Pd-catalyzed C–H bond functionalization for the annulative π-extension of 1,8-dibromonaphthalene: a one pot access to fluoranthene derivatives

Nahed Ketata, Linhao Liu, Ridha Ben Salem and Henri Doucet

Beilstein J. Org. Chem. **2024,** *20,* 427–435. doi:10.3762/bjoc.20.37

Copies of ^{1}H, ^{19}F, and ^{13}C NMR spectra

License and Terms: This is a supporting information file under the terms of the Creative Commons Attribution License (https://creativecommons.org/licenses/by/4.0). Please note that the reuse, redistribution and reproduction in particular requires that the author(s) and source are credited and that individual graphics may be subject to special legal provisions.
The license is subject to the *Beilstein Journal of Organic Chemistry* terms and conditions: (https://www.beilstein-journals.org/bjoc/terms)

7,8,9,10-Tetrafluorofluoranthene (1)

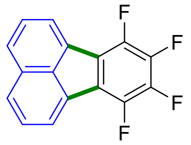

^{1}H NMR (500 MHz, $CDCl_3$)

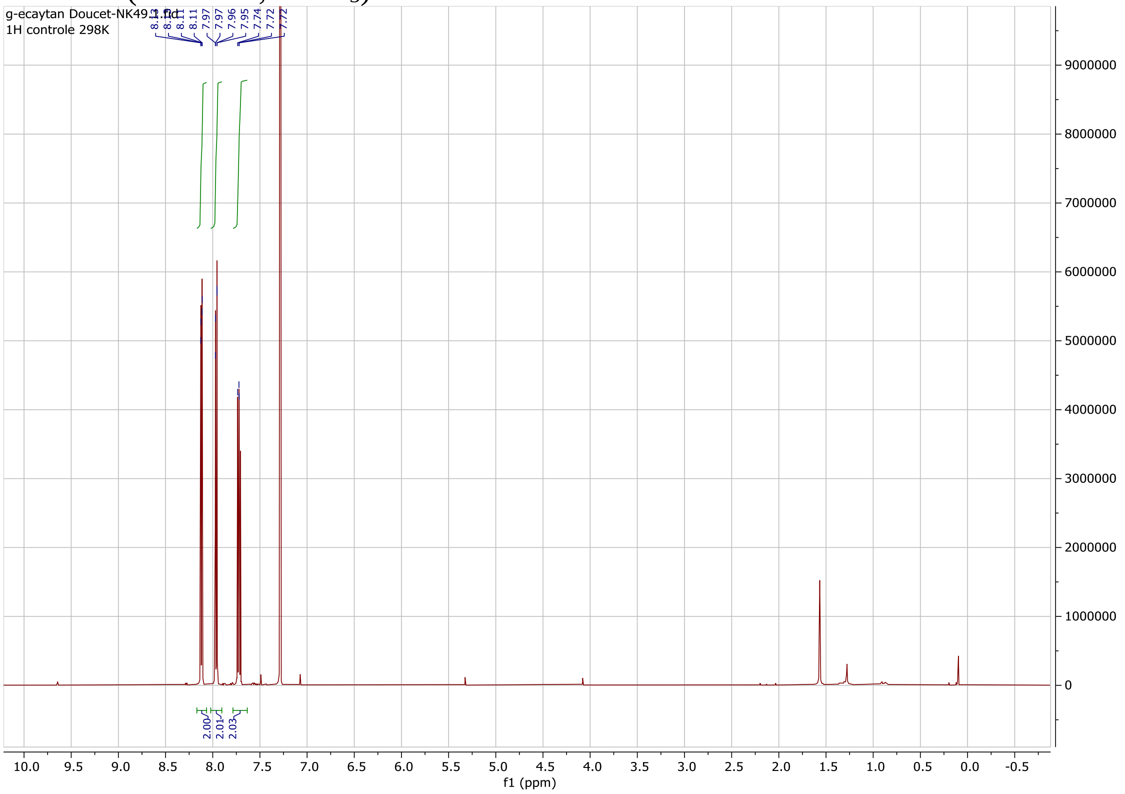

^{19}F NMR (376 MHz, $CDCl_3$)

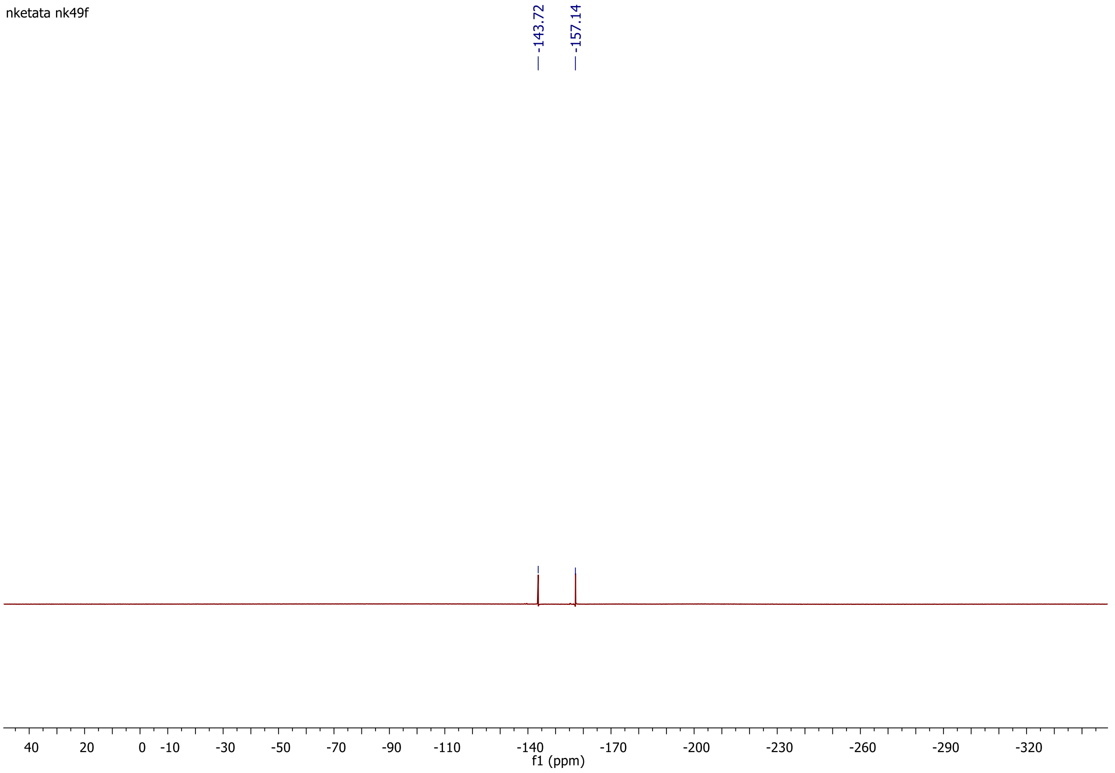

^{13}C NMR (125 MHz, $CDCl_3$)

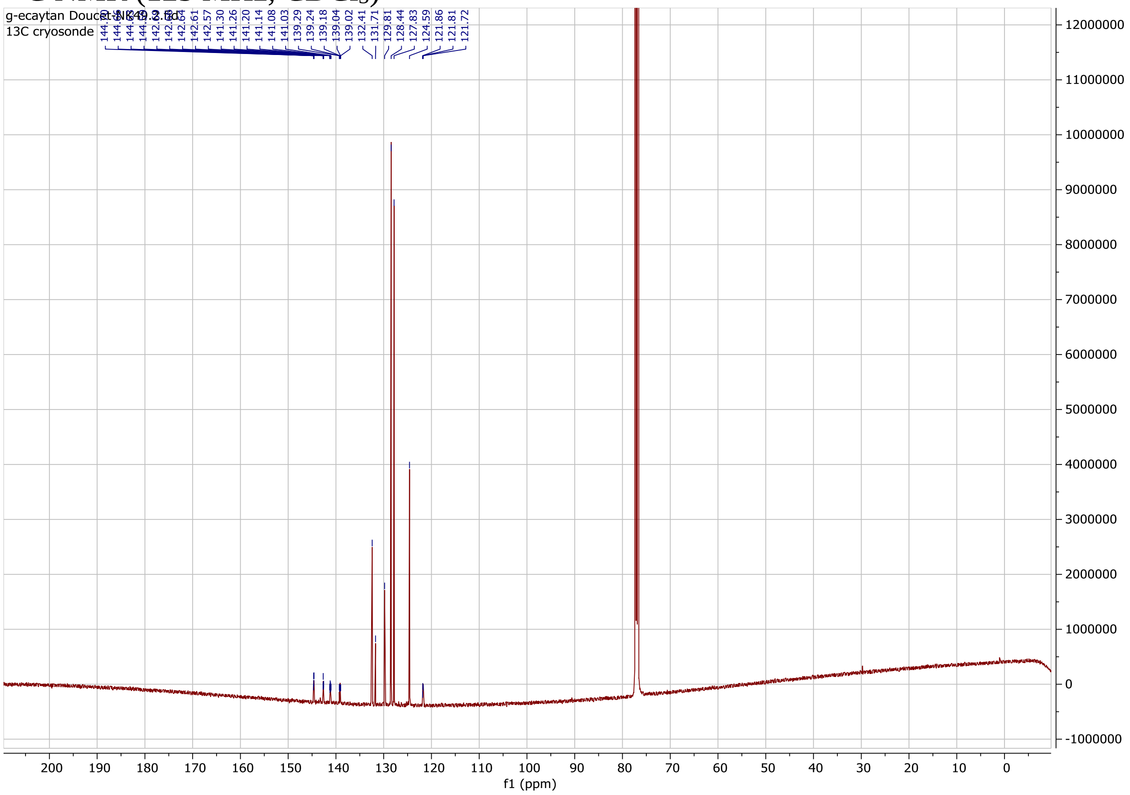

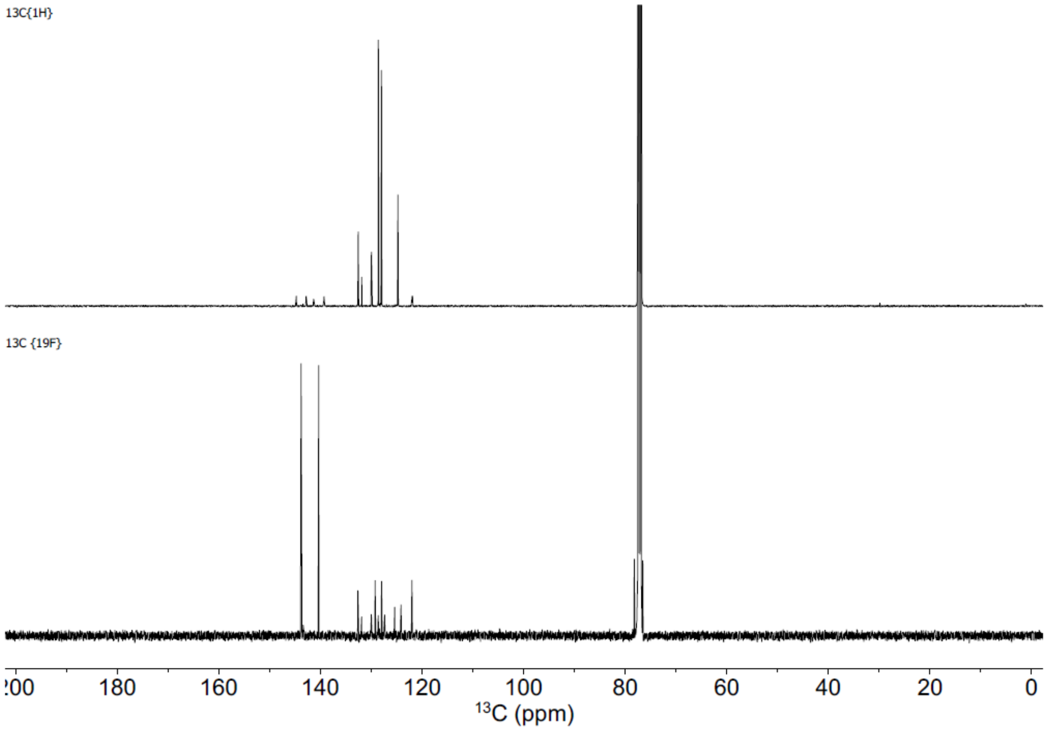

7,10-Difluorofluoranthene (2)

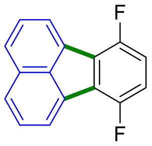

^{1}H NMR (400 MHz, $CDCl_3$)

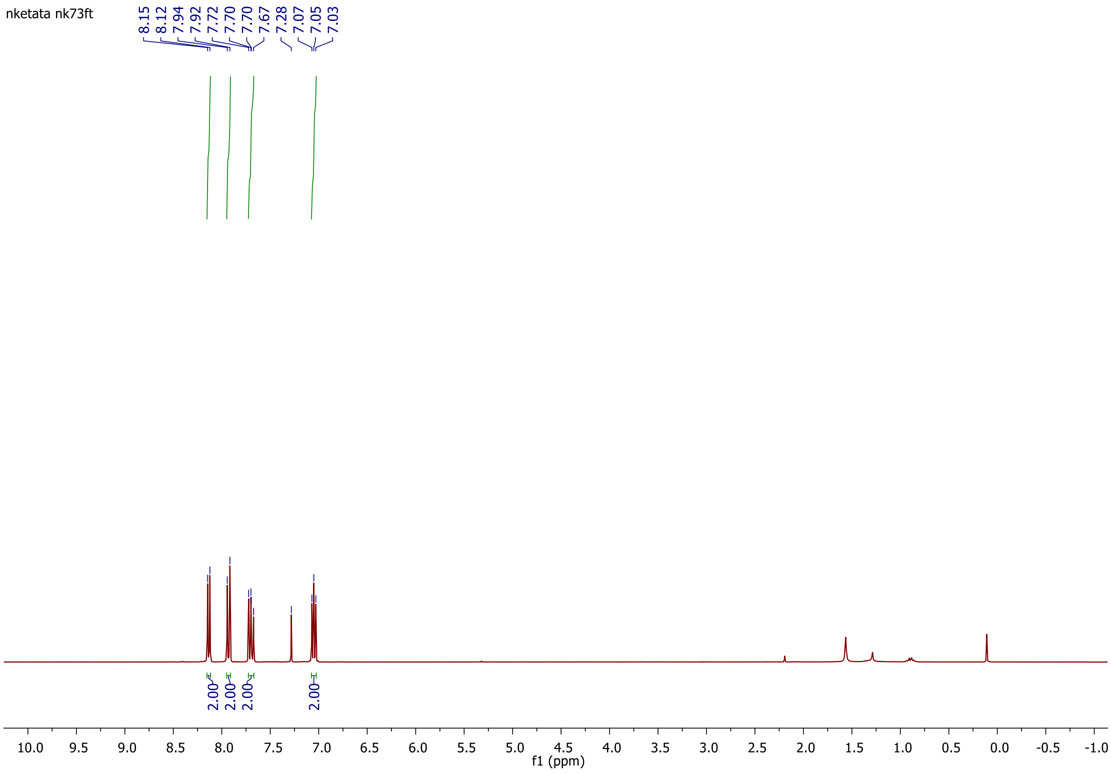

^{19}F NMR (376 MHz, $CDCl_3$)

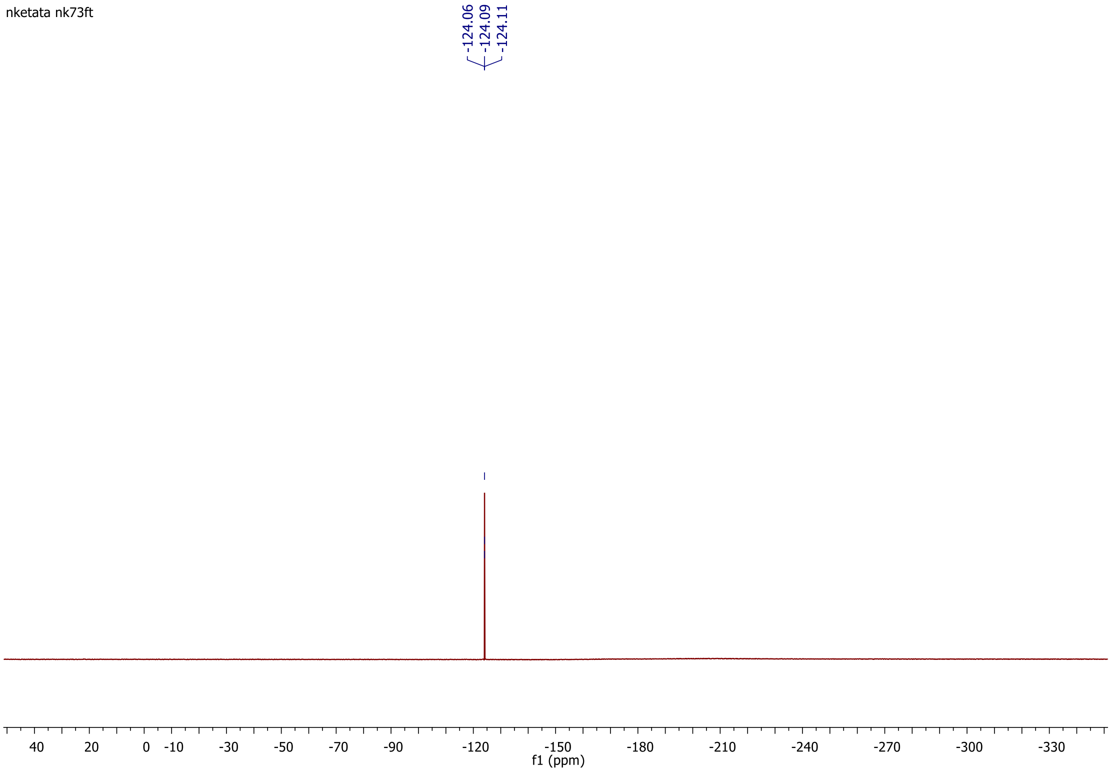

^{13}C NMR (100 MHz, $CDCl_3$)

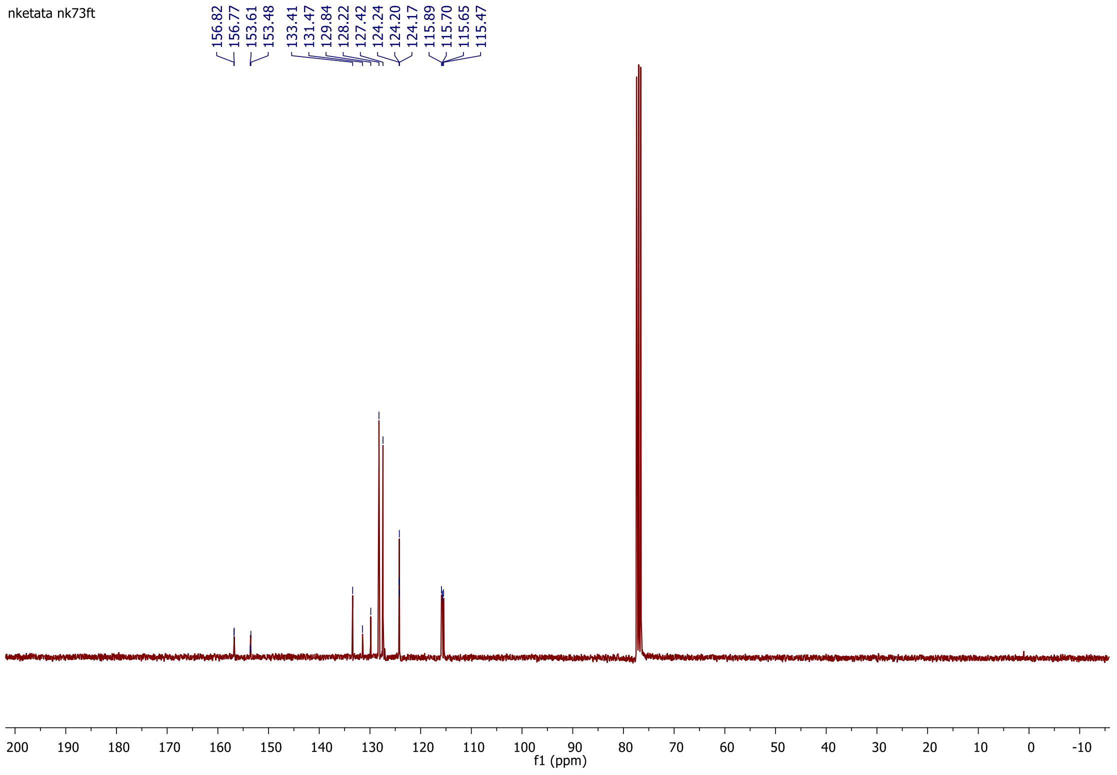

7-Fluoro-10-methoxyfluoranthene (3)

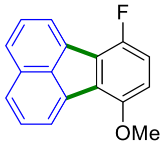

^{1}H NMR (400MHz, $CDCl_3$)

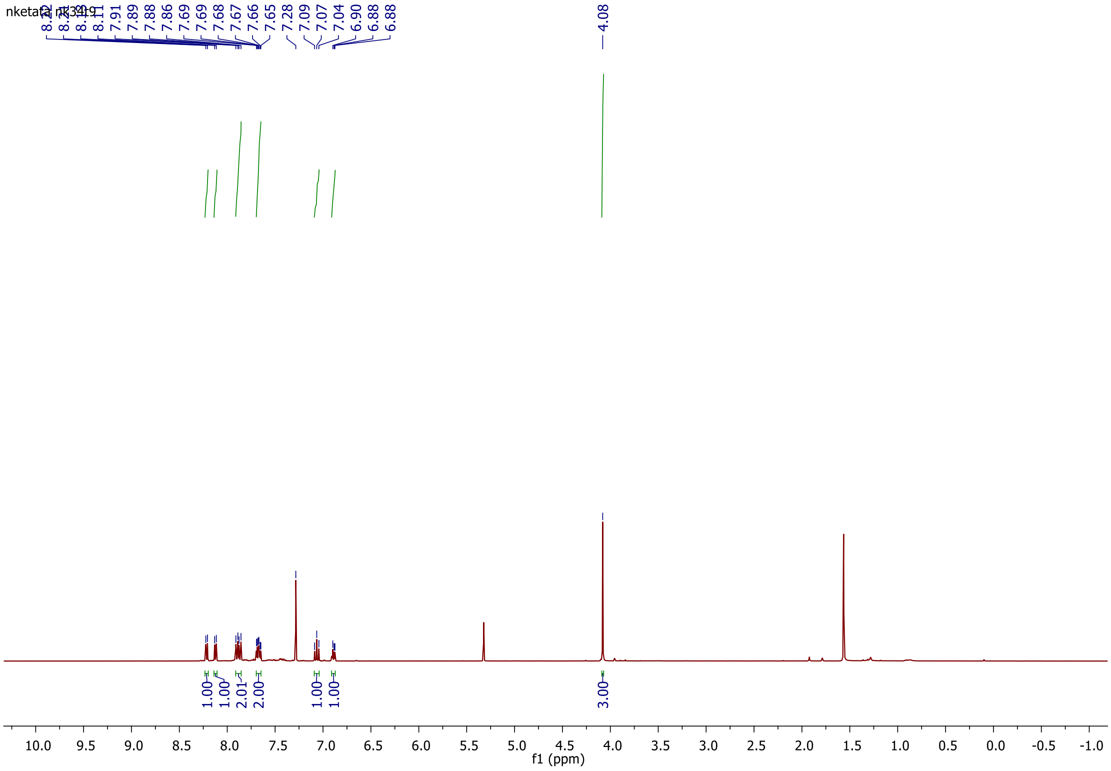

^{19}F NMR (376 MHz, $CDCl_3$)

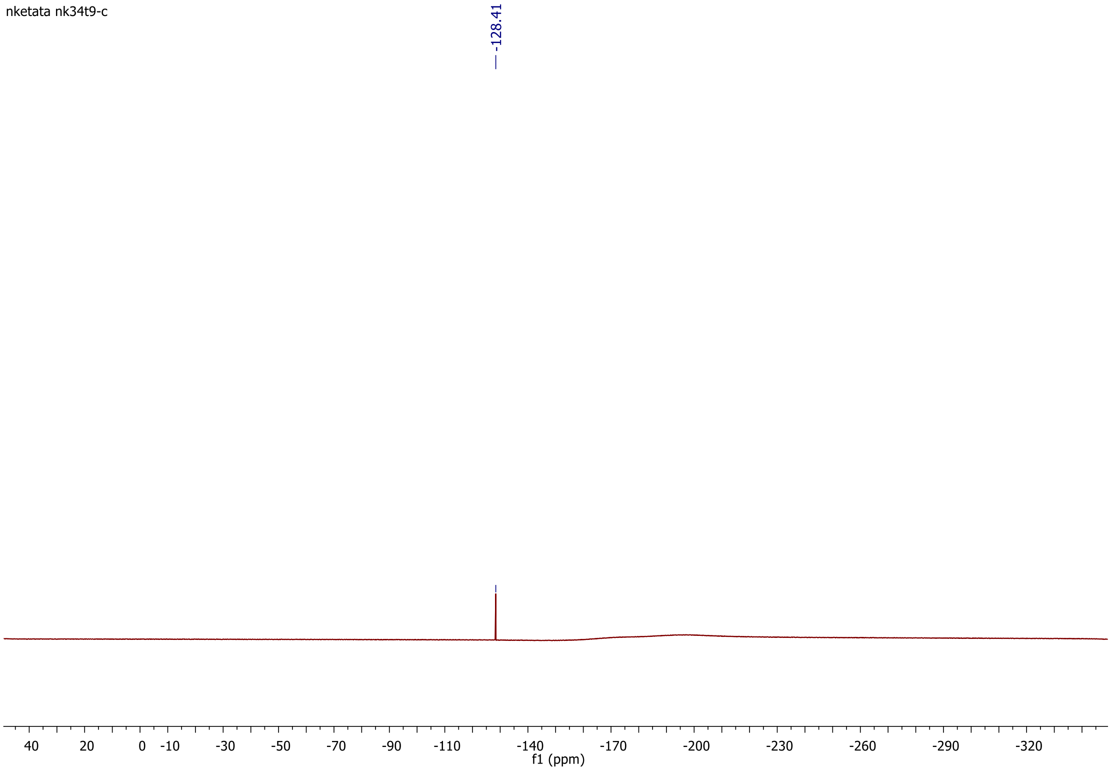

^{13}C NMR (100 MHz, $CDCl_3$)

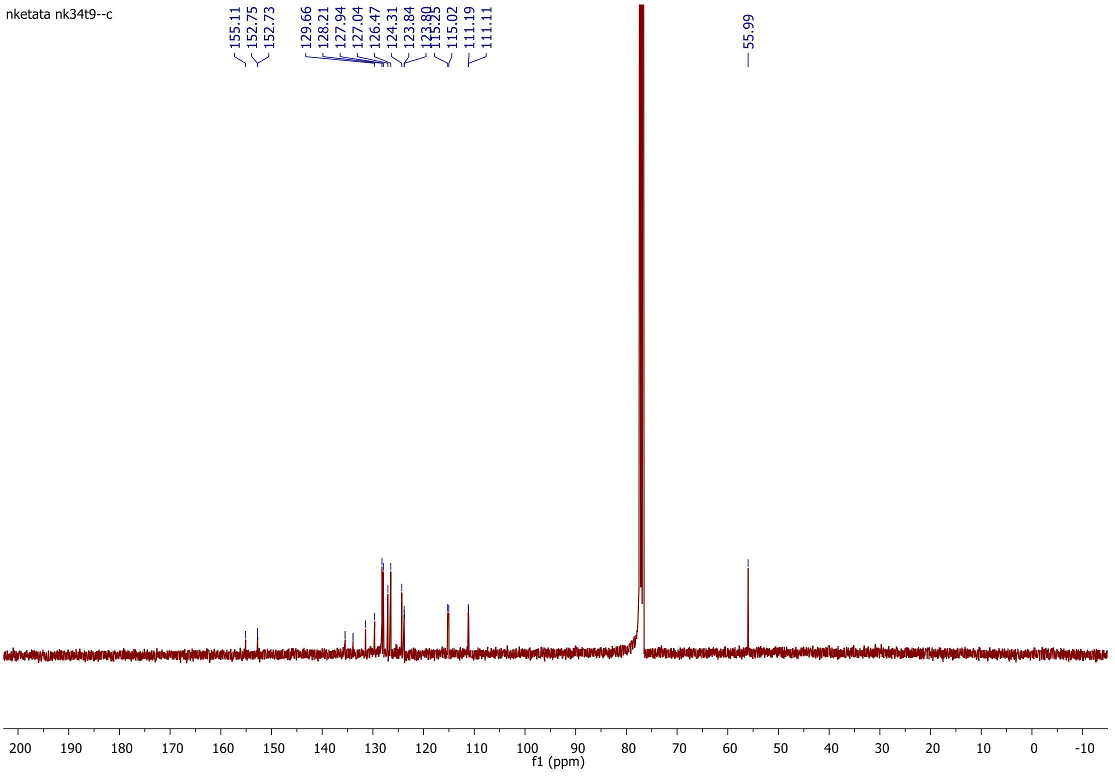

7-Fluoro-10-methylfluoranthene (4)

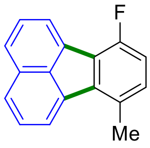

^{1}H NMR (400MHz, $CDCl_3$)

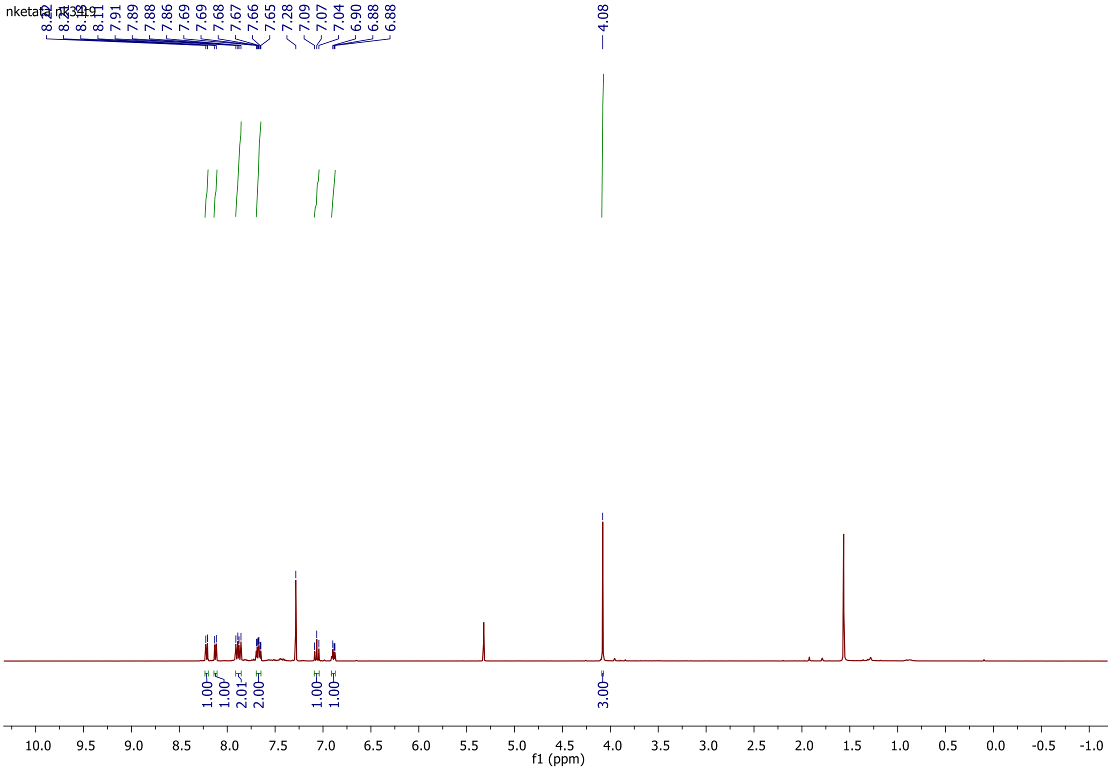

^{19}F NMR (376 MHz, $CDCl_3$)

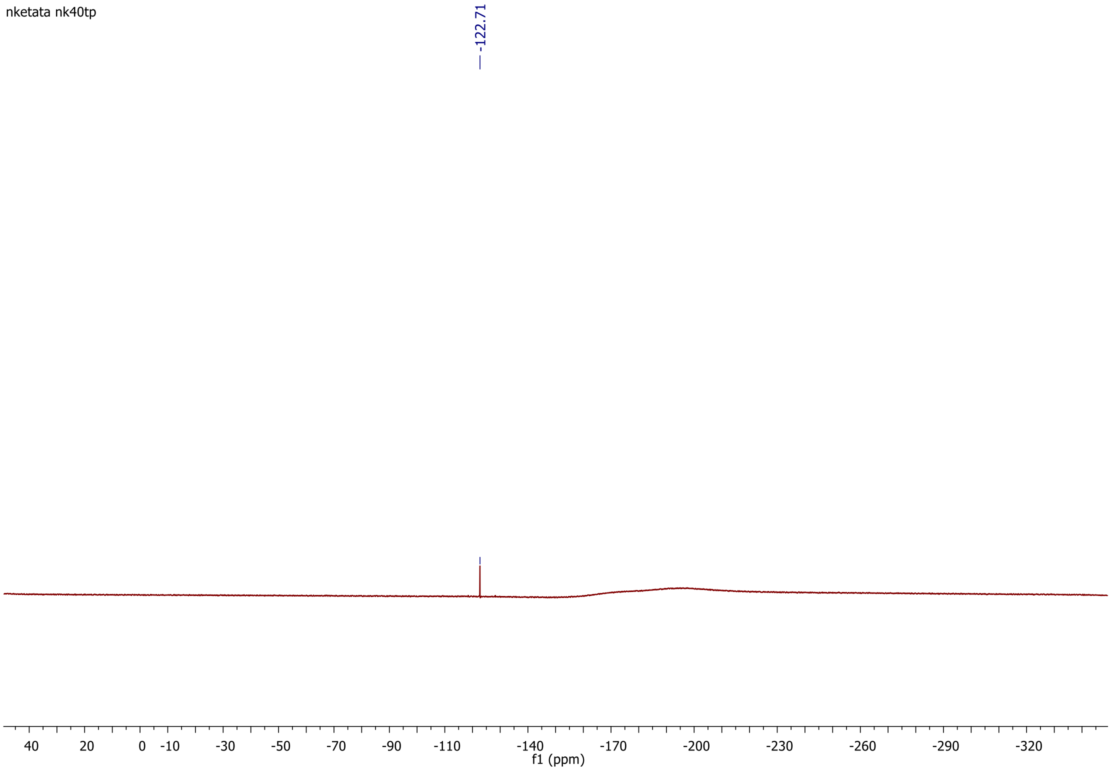

^{13}C NMR (100 MHz, $CDCl_3$)

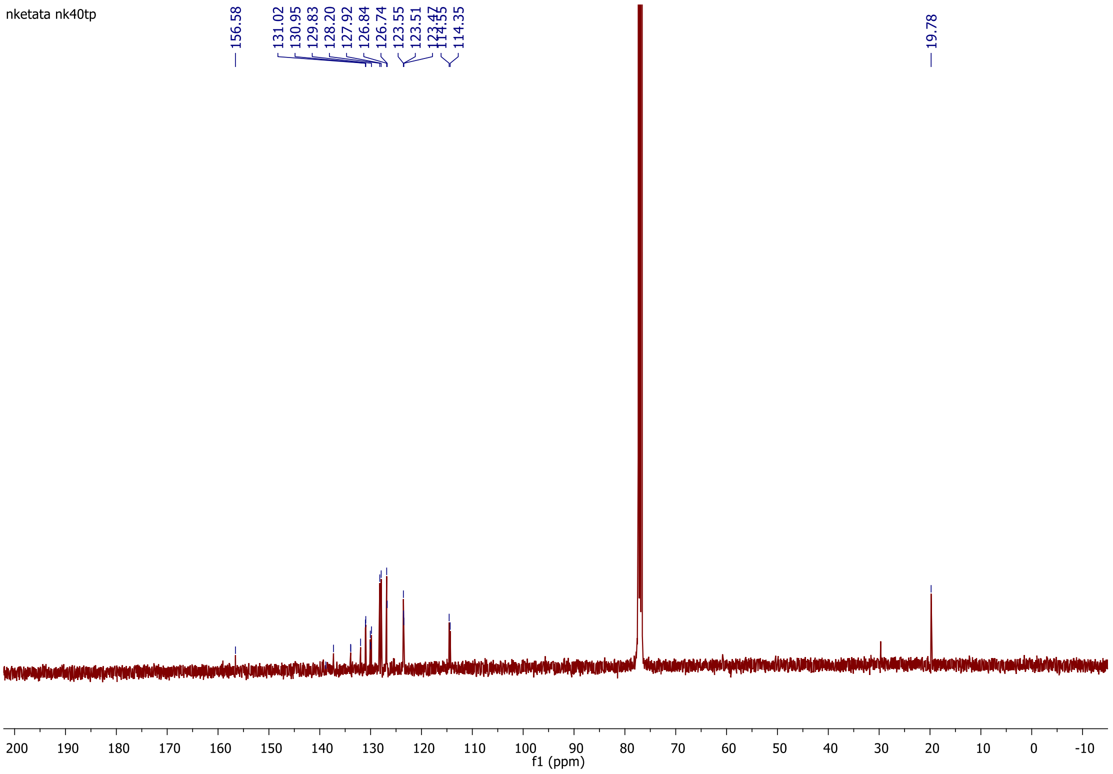

7-Chloro-10-fluorofluoranthene (5)

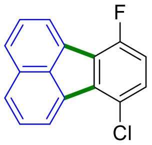

^{1}H NMR (400MHz, $CDCl_3$)

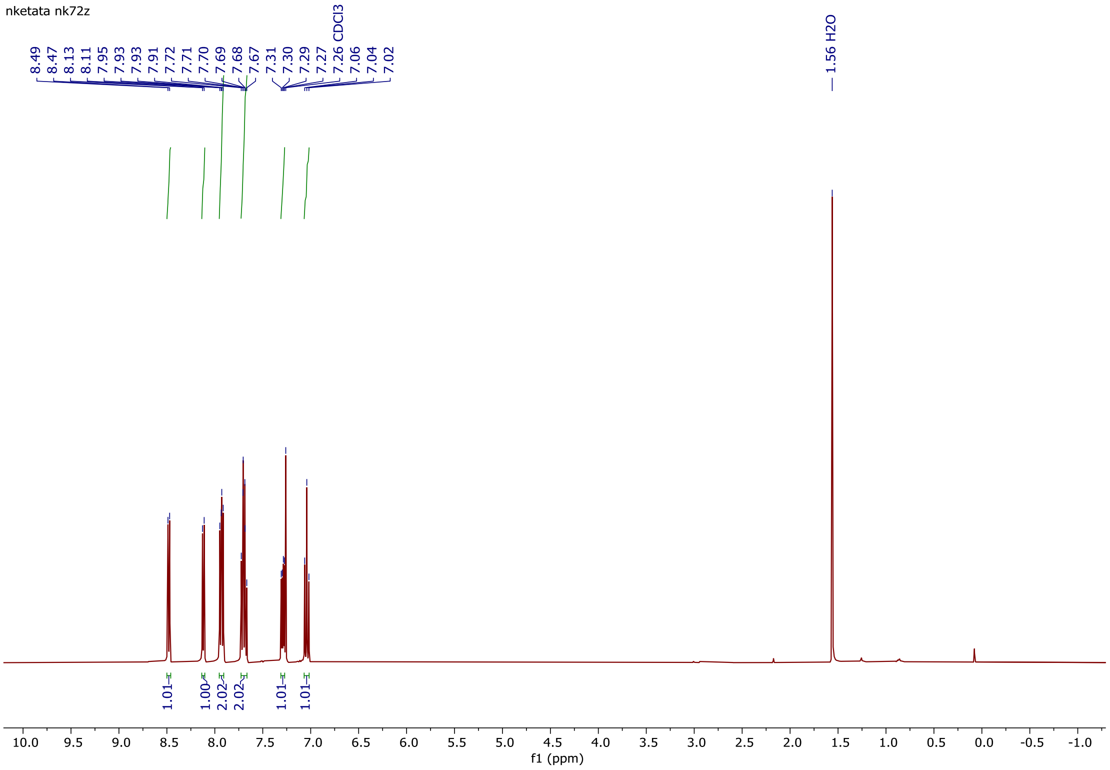

^{19}F NMR (376 MHz, $CDCl_3$)

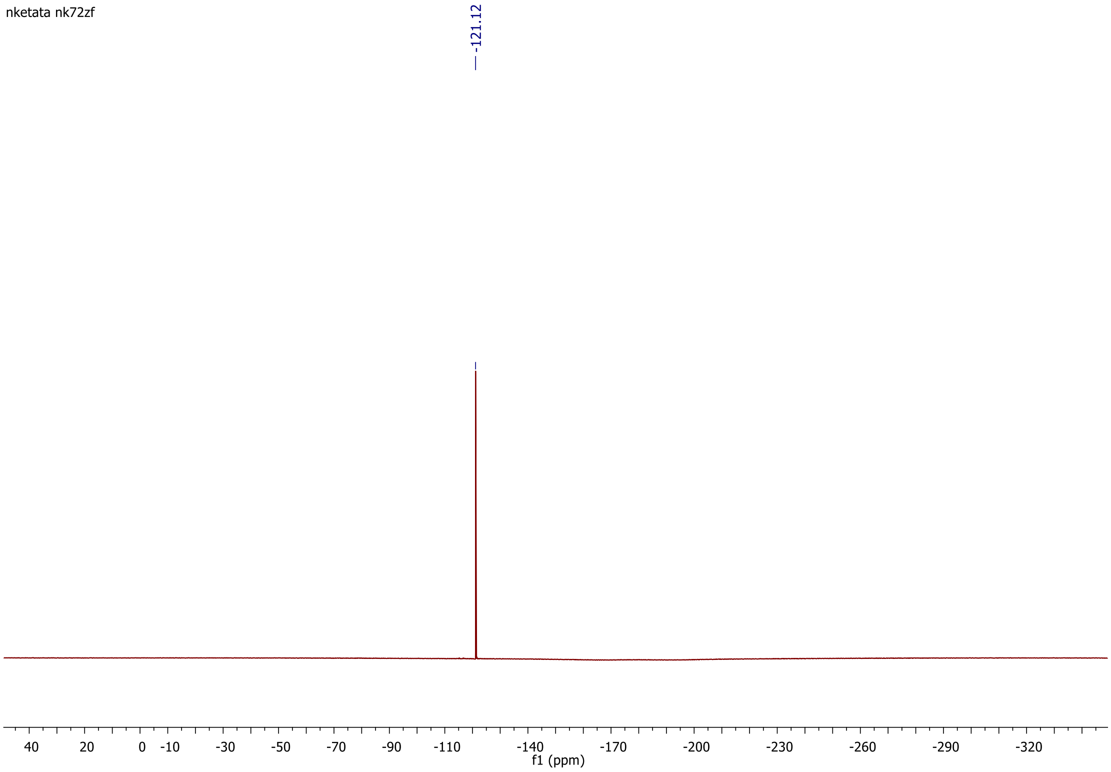

^{13}C NMR (100 MHz, $CDCl_3$)

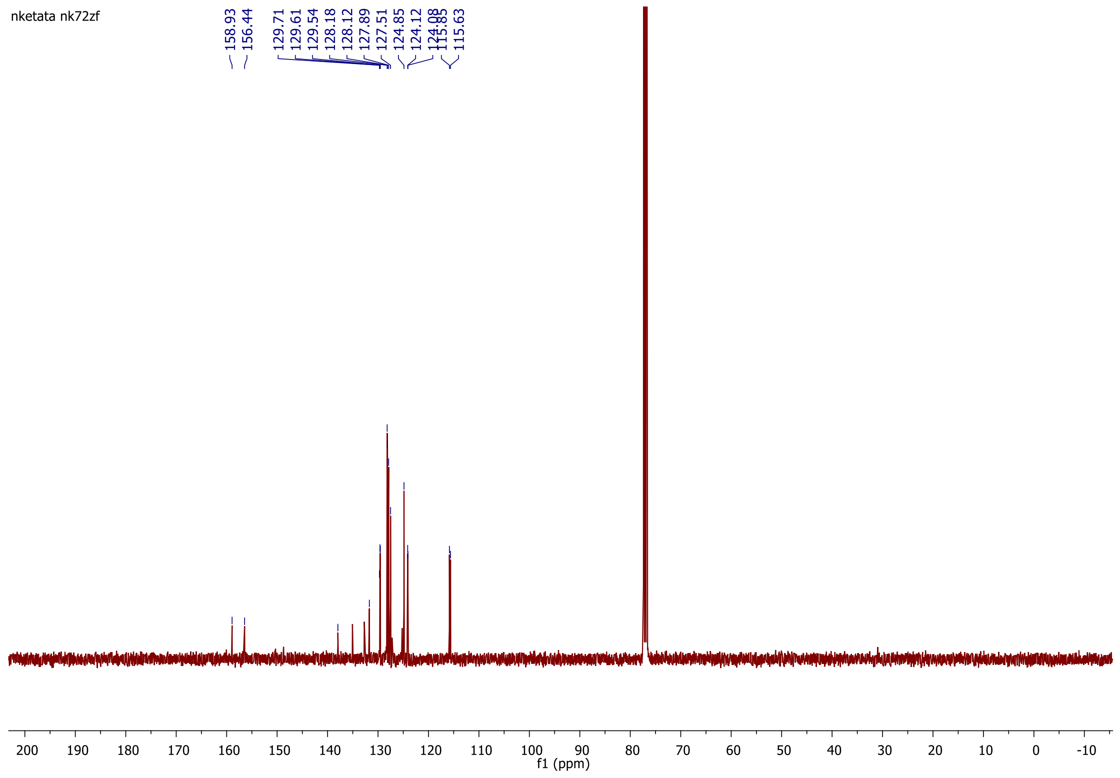

Cyclopropyl(10-fluorofluoranthen-7-yl)methanone (6)

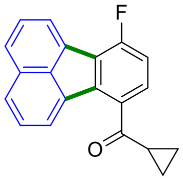

^{1}H NMR (400MHz, $CDCl_3$)

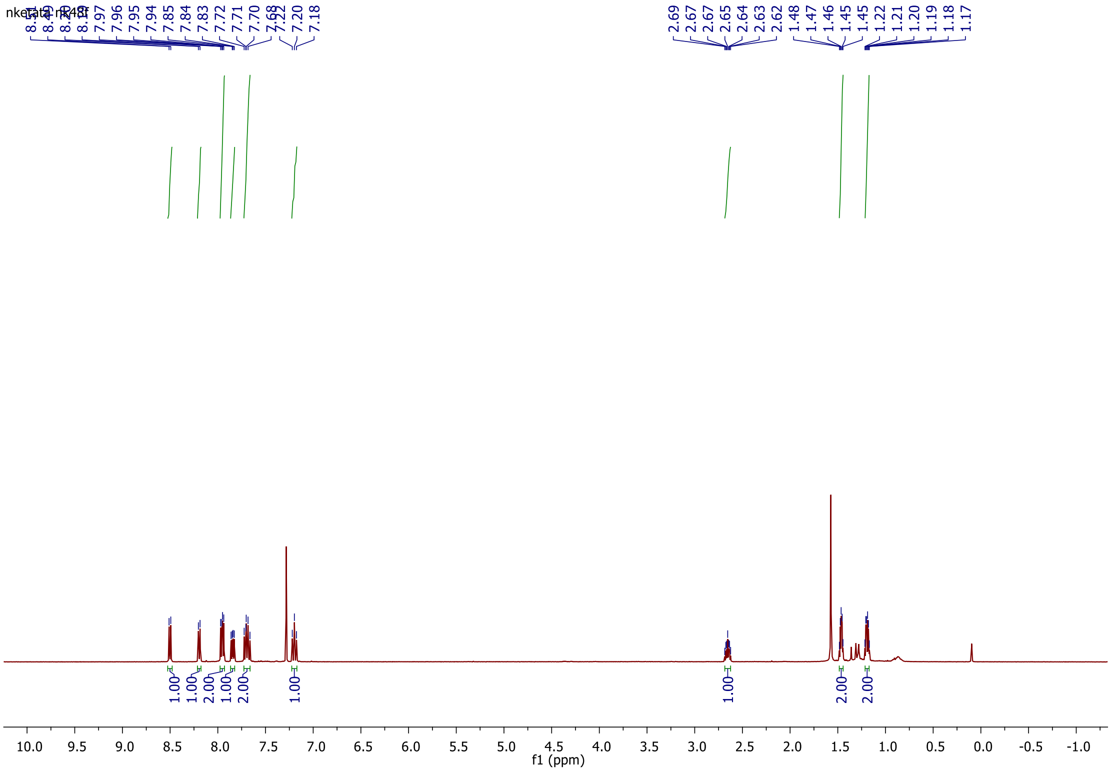

^{19}F NMR (376 MHz, $CDCl_3$)

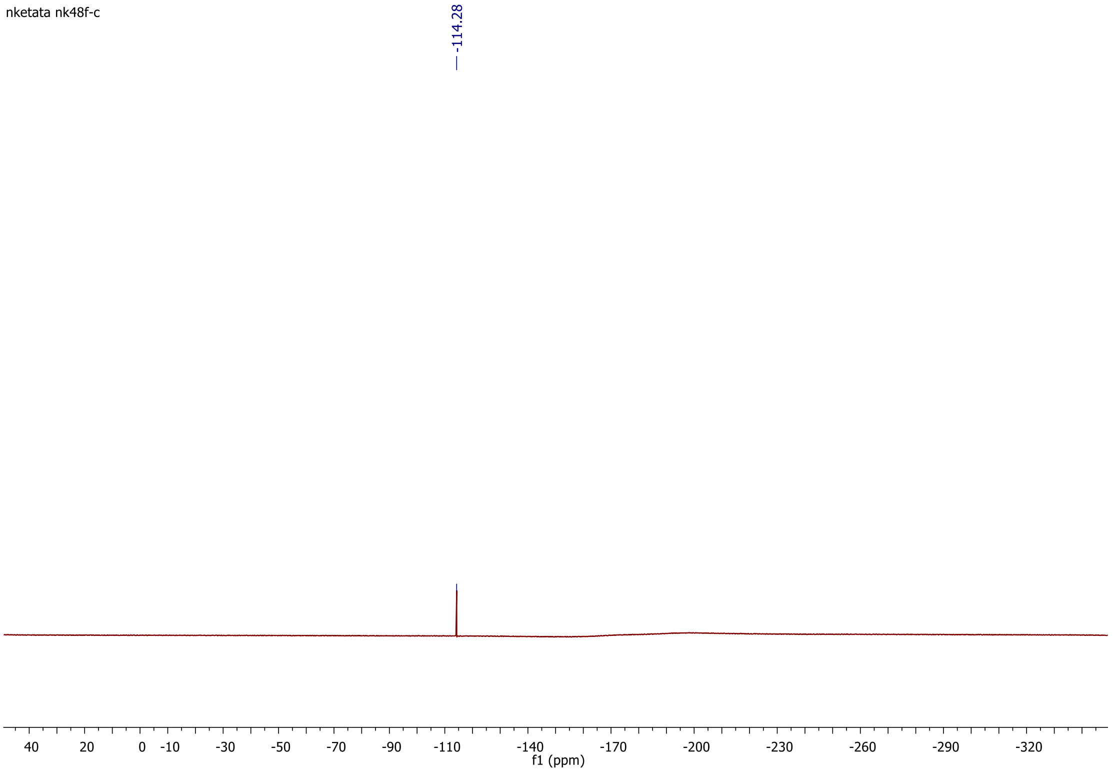

^{13}C NMR (100 MHz, $CDCl_3$)

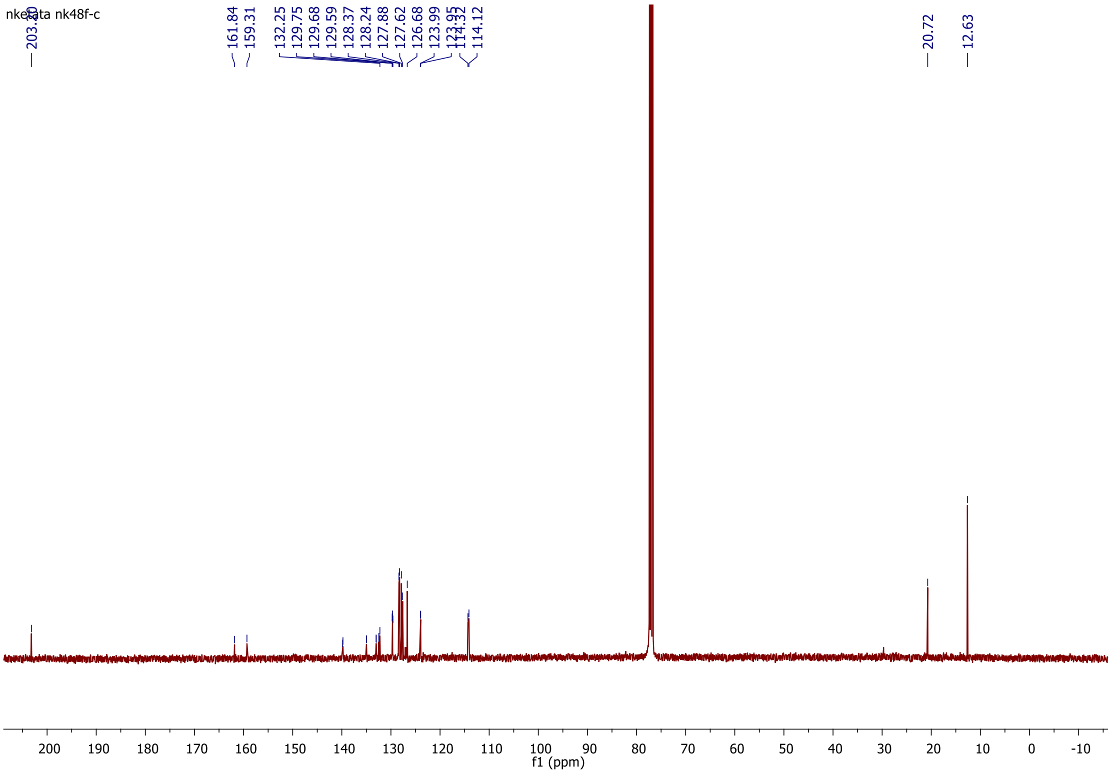

8-Chloro-7-fluorofluoranthene (7)

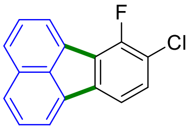

^{1}H NMR (400MHz, $CDCl_3$)

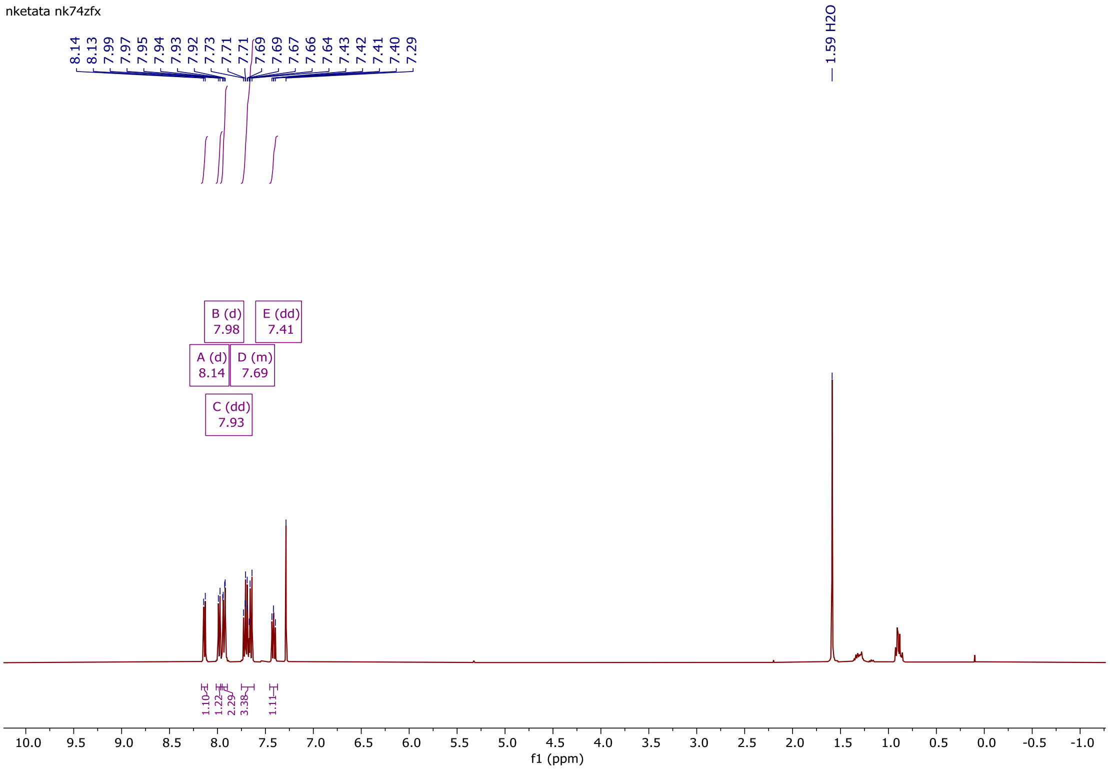

^{19}F NMR (376 MHz, $CDCl_3$)

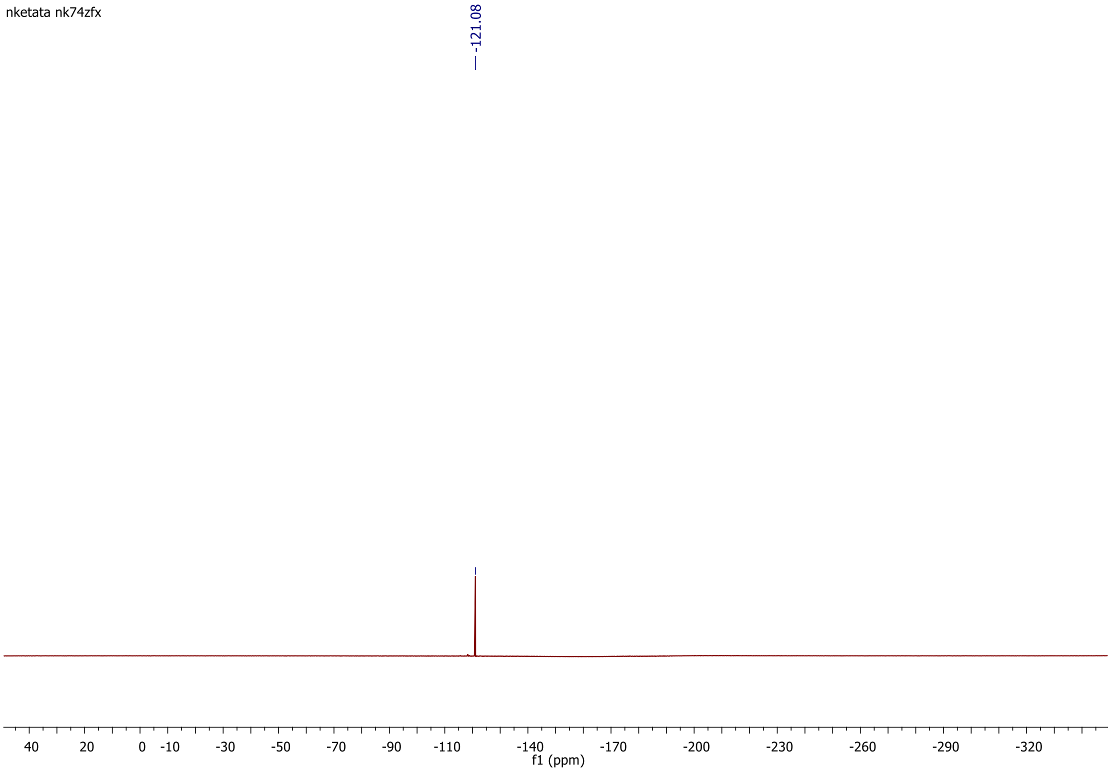

^{13}C NMR (100 MHz, $CDCl_3$)

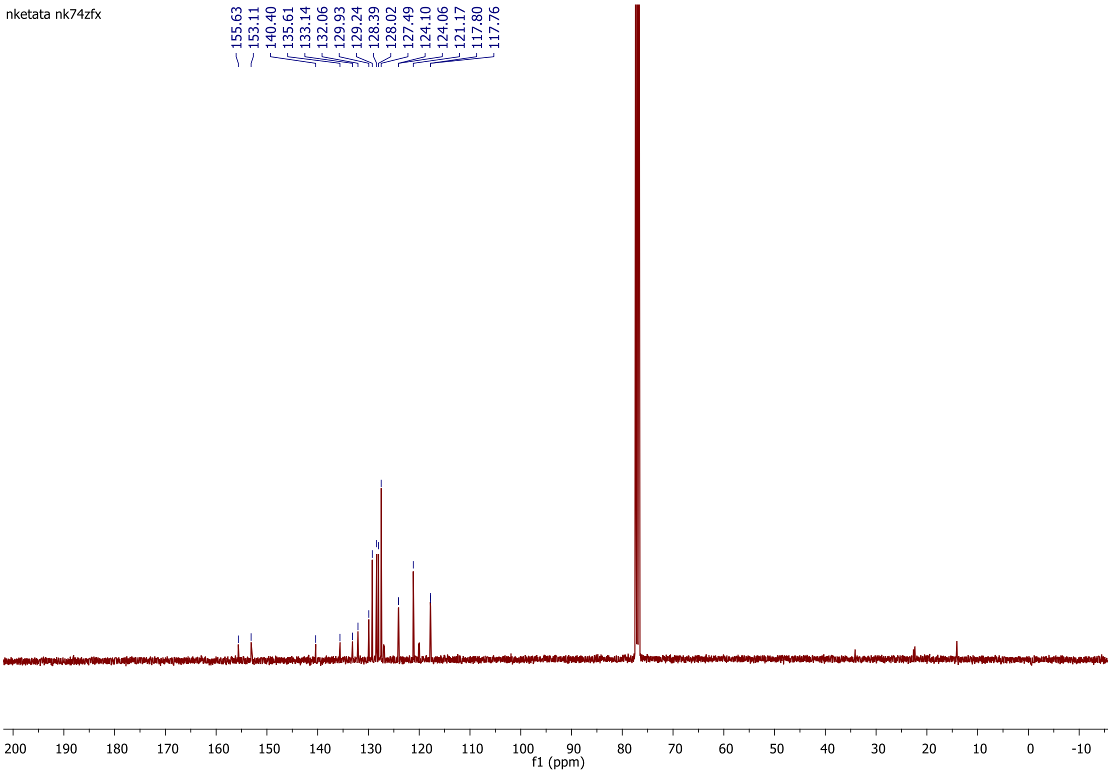

7-Fluorofluoranthene-8-carbonitrile (8)

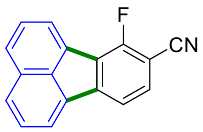

^{1}H NMR (400MHz, $CDCl_3$)

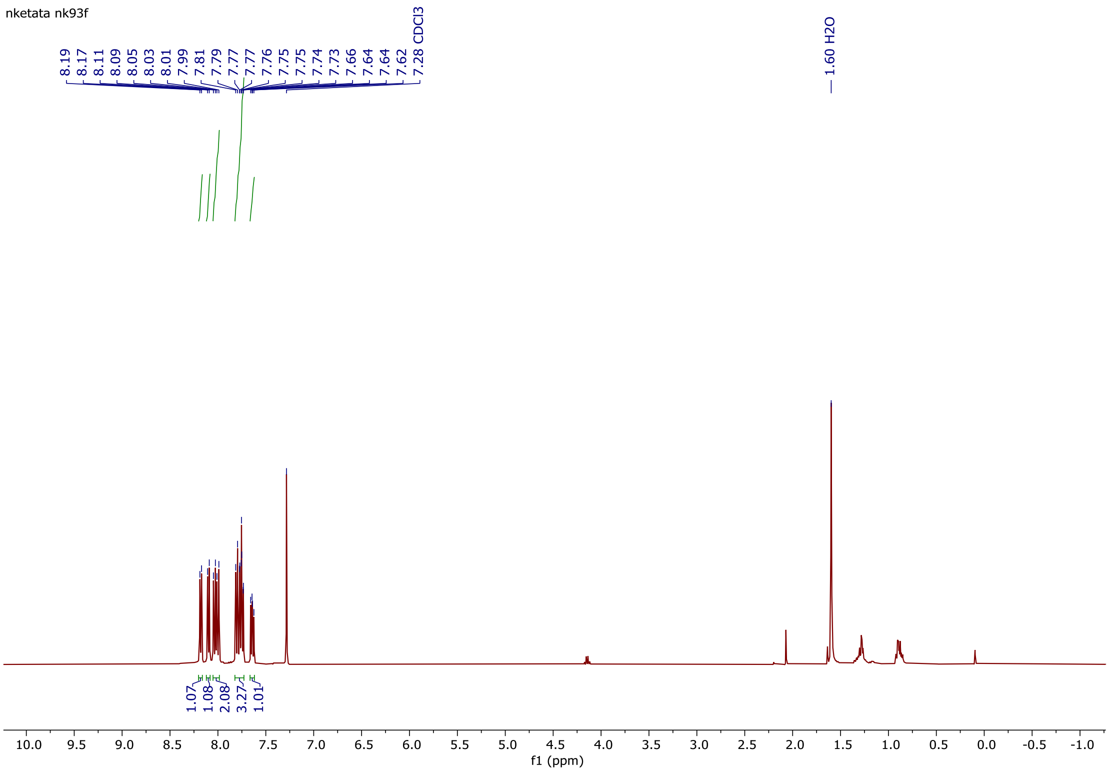

^{19}F NMR (376 MHz, $CDCl_3$)

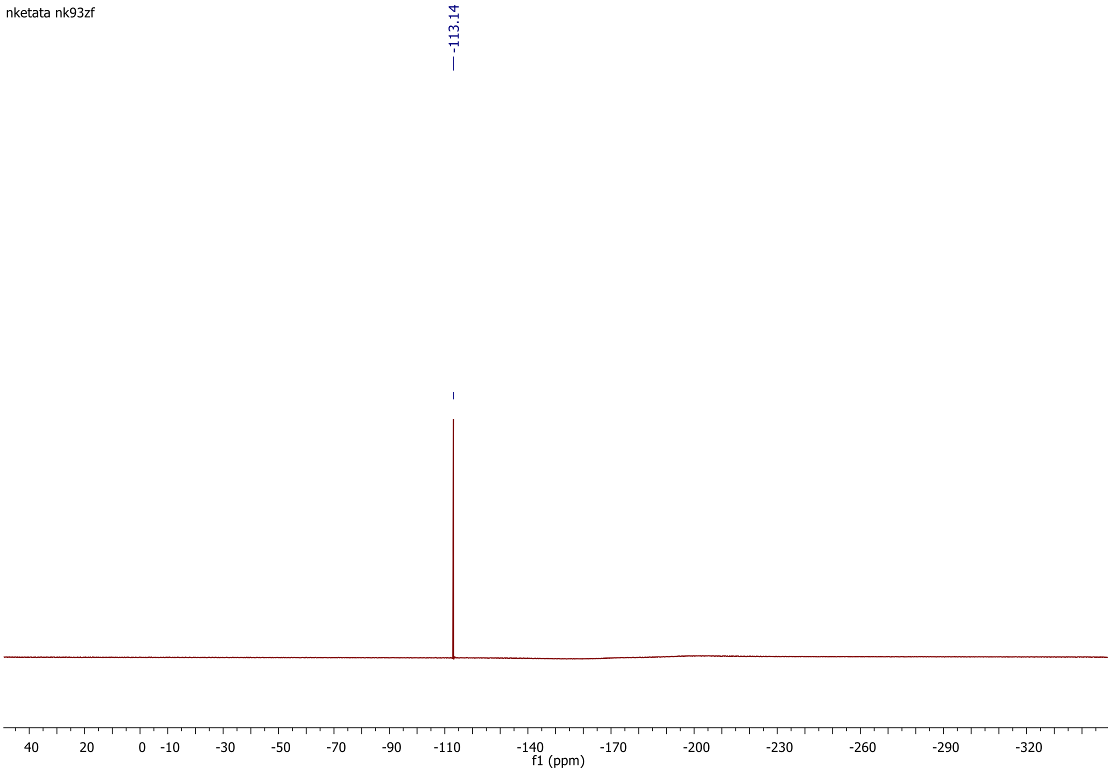

^{13}C NMR (100 MHz, $CDCl_3$)

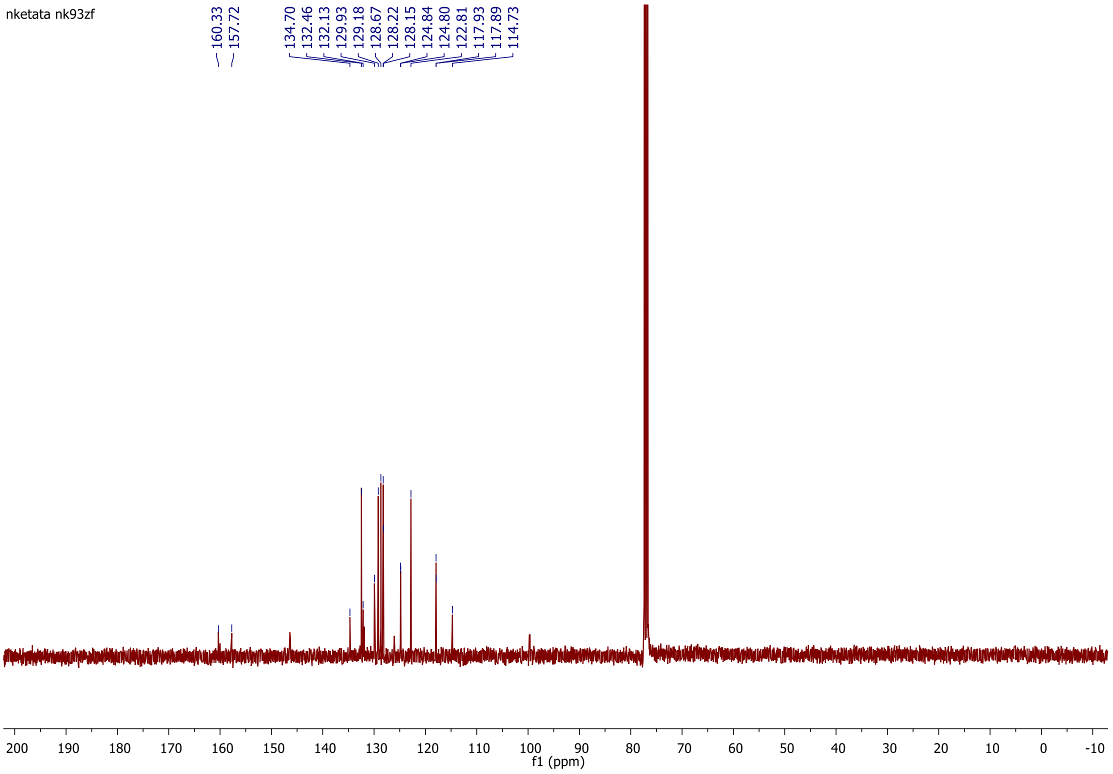

7,8,9-Trifluorofluoranthene (9)

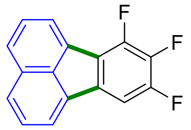

^{1}H NMR (500MHz, $CDCl_3$)

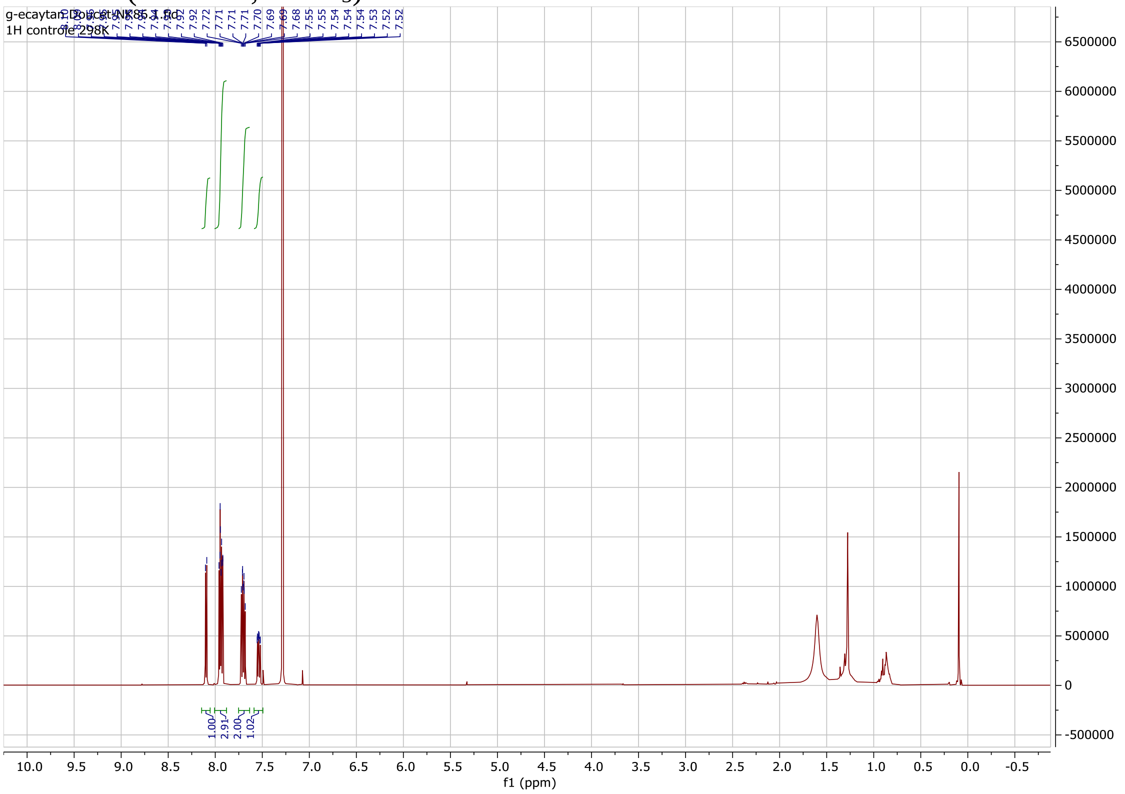

^{19}F NMR (376 MHz, $CDCl_3$)

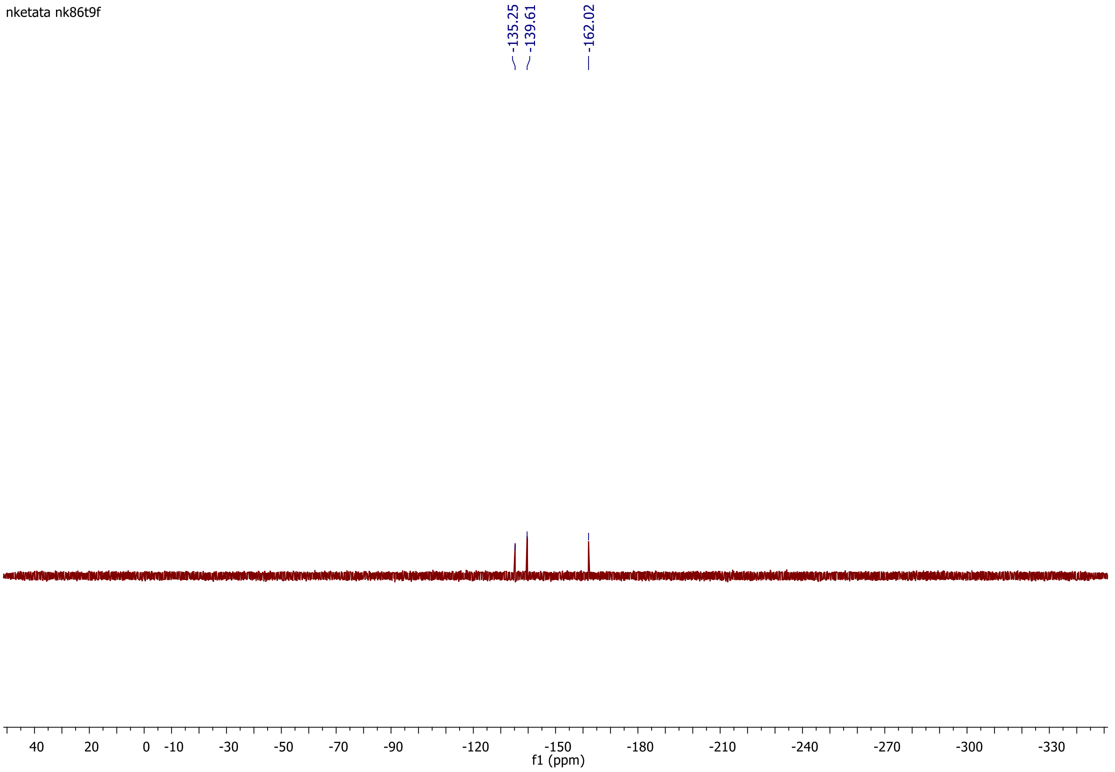

^{13}C NMR (125 MHz, $CDCl_3$)

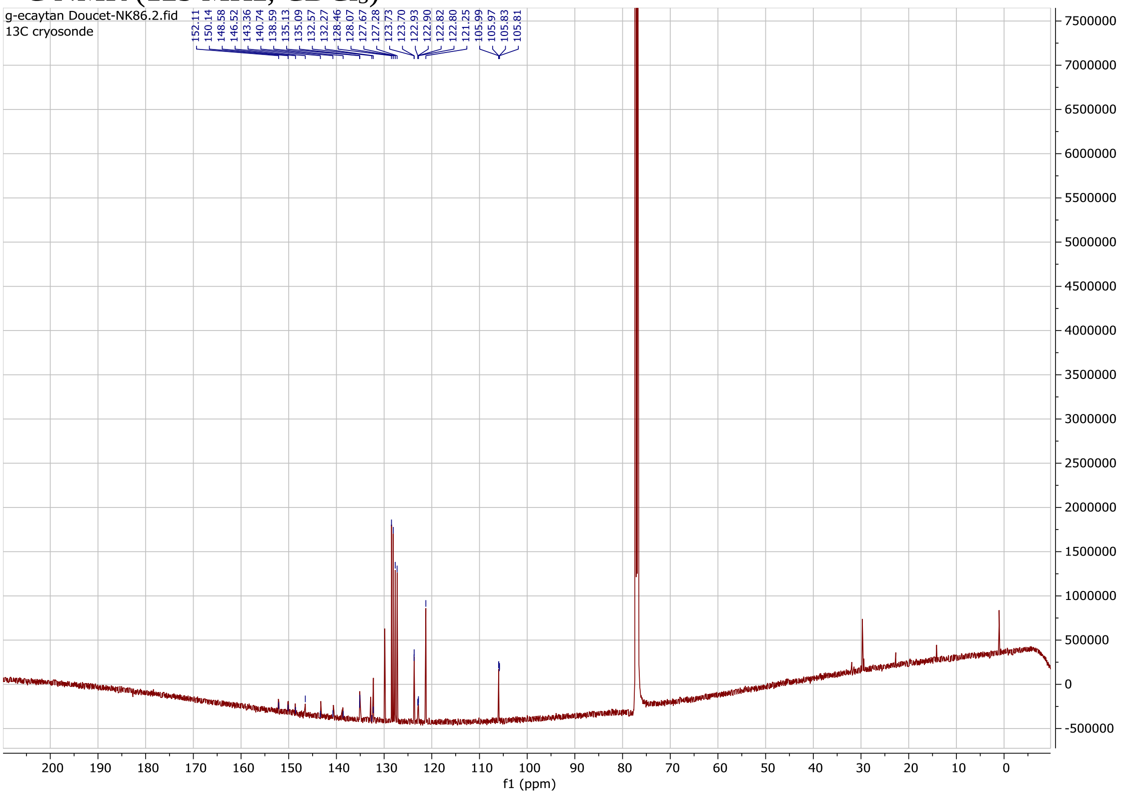

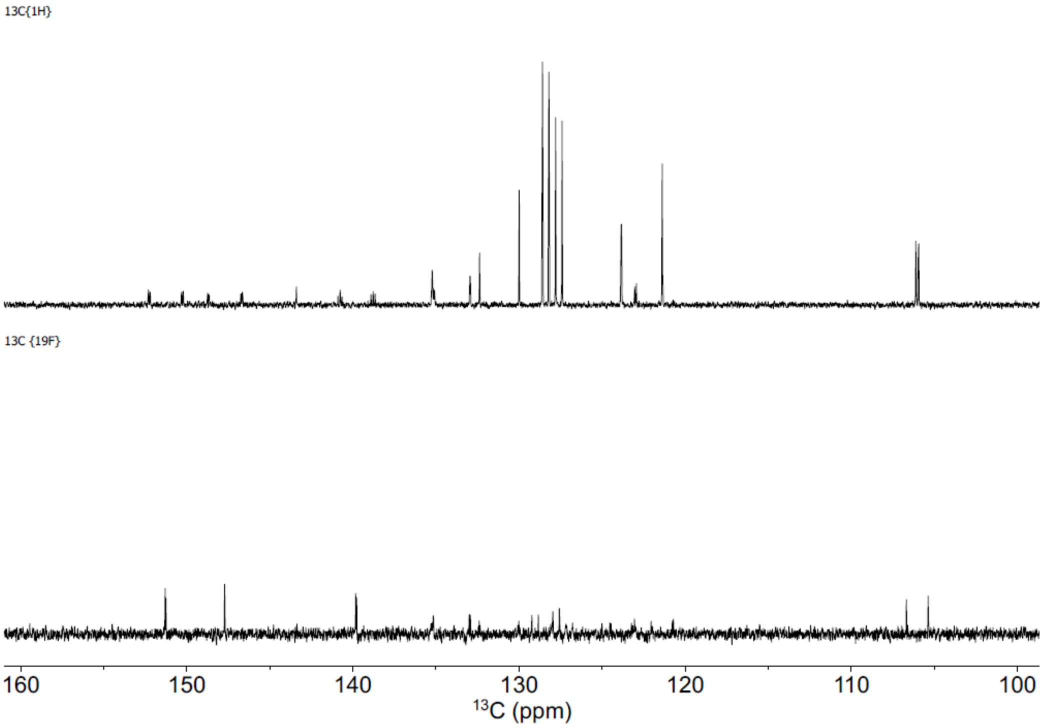

7,8-Difluoro-9-methylfluoranthene (10)

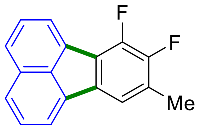

^{1}H NMR (400MHz, $CDCl_3$)

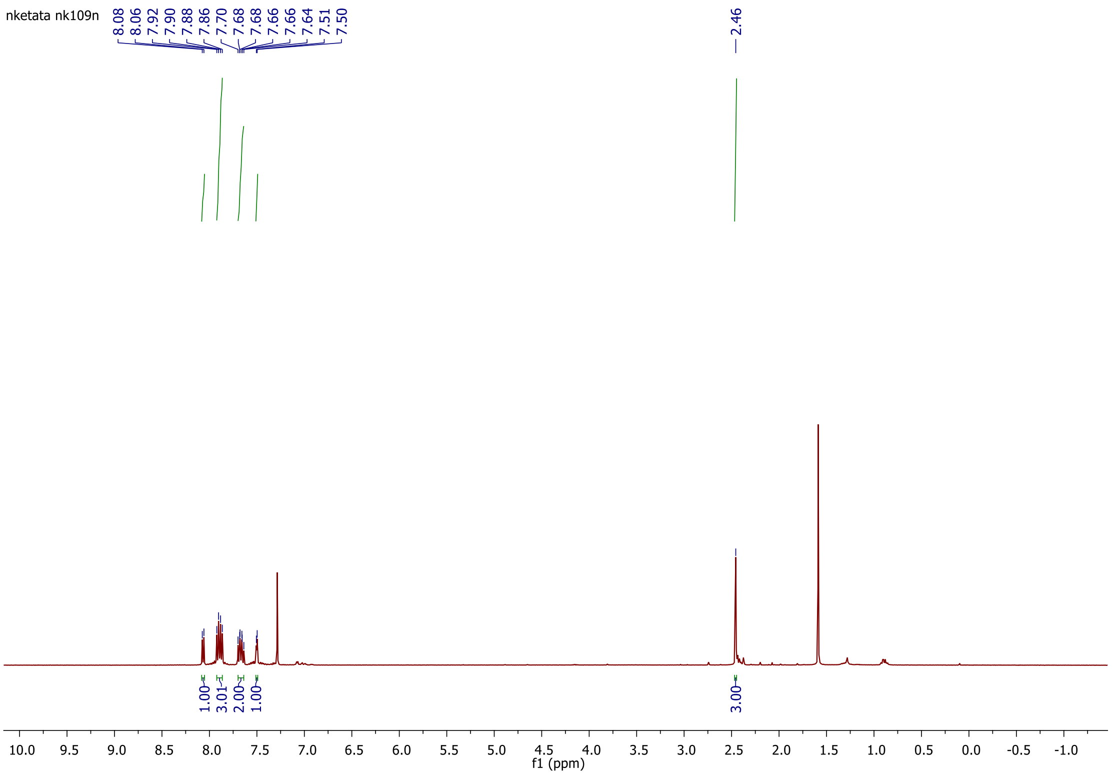

^{19}F NMR (376 MHz, $CDCl_3$)

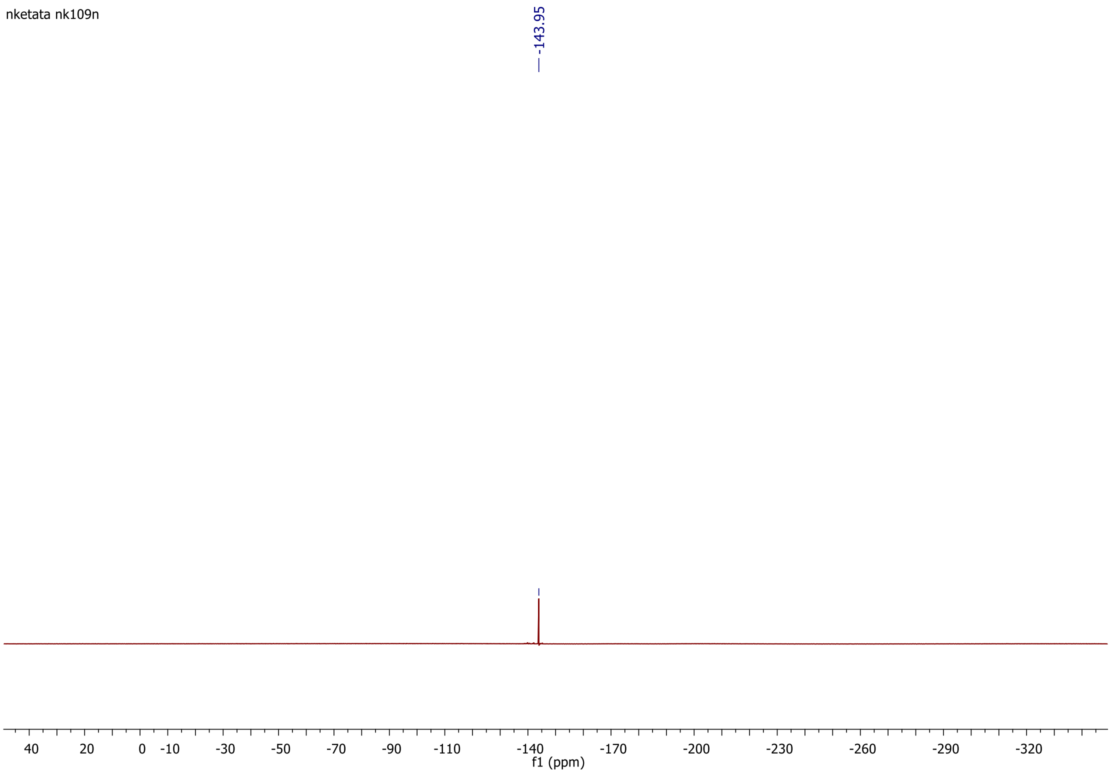

^{13}C NMR (100 MHz, $CDCl_3$)

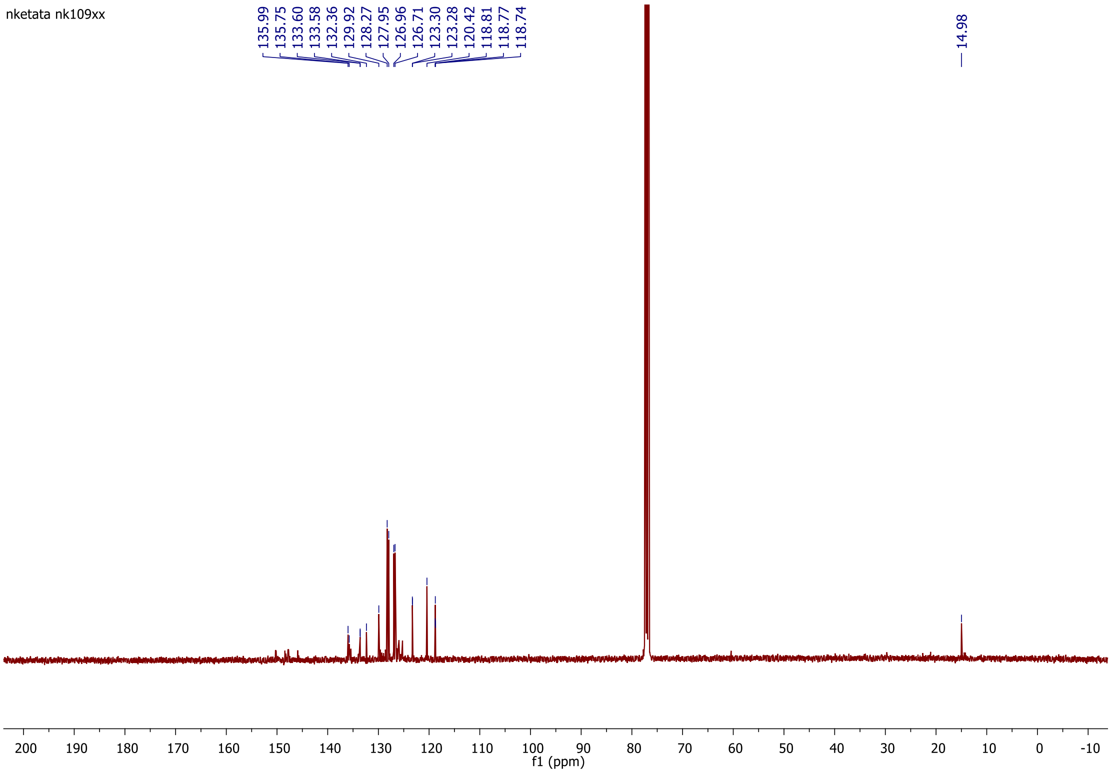

8-Chloro-7-fluoro-10-methoxyfluoranthene (11)

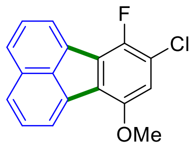

^{1}H NMR (400MHz, $CDCl_3$)

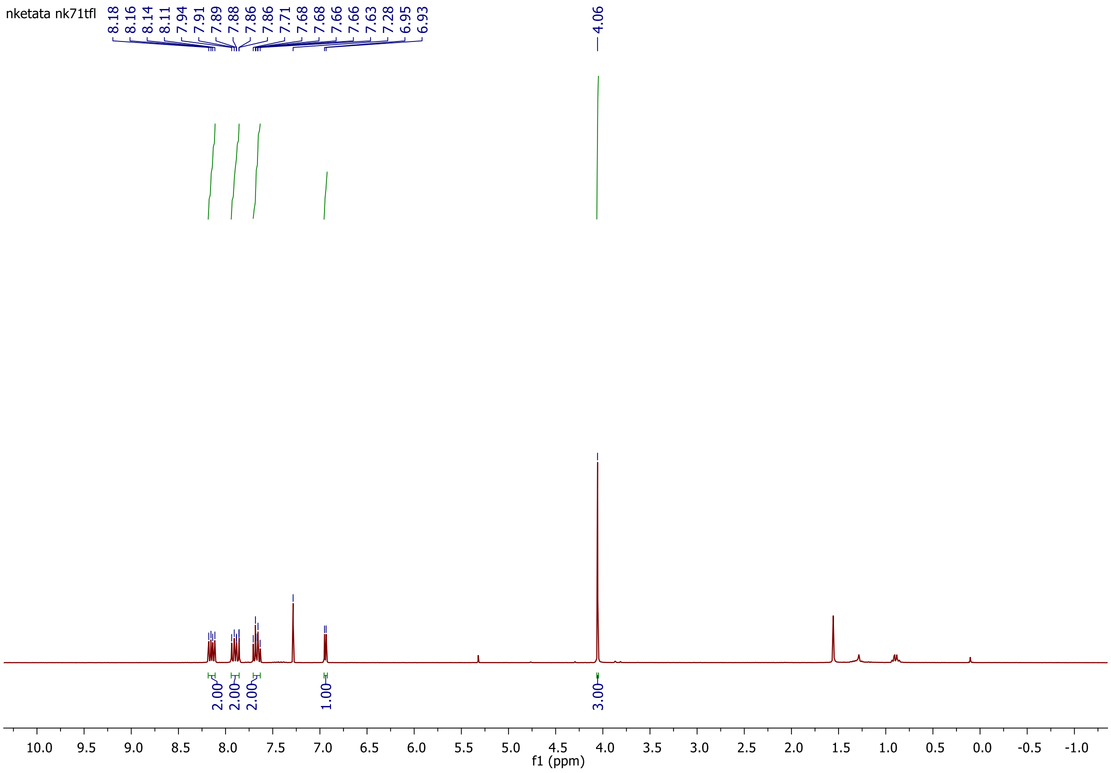

^{19}F NMR (376 MHz, $CDCl_3$)

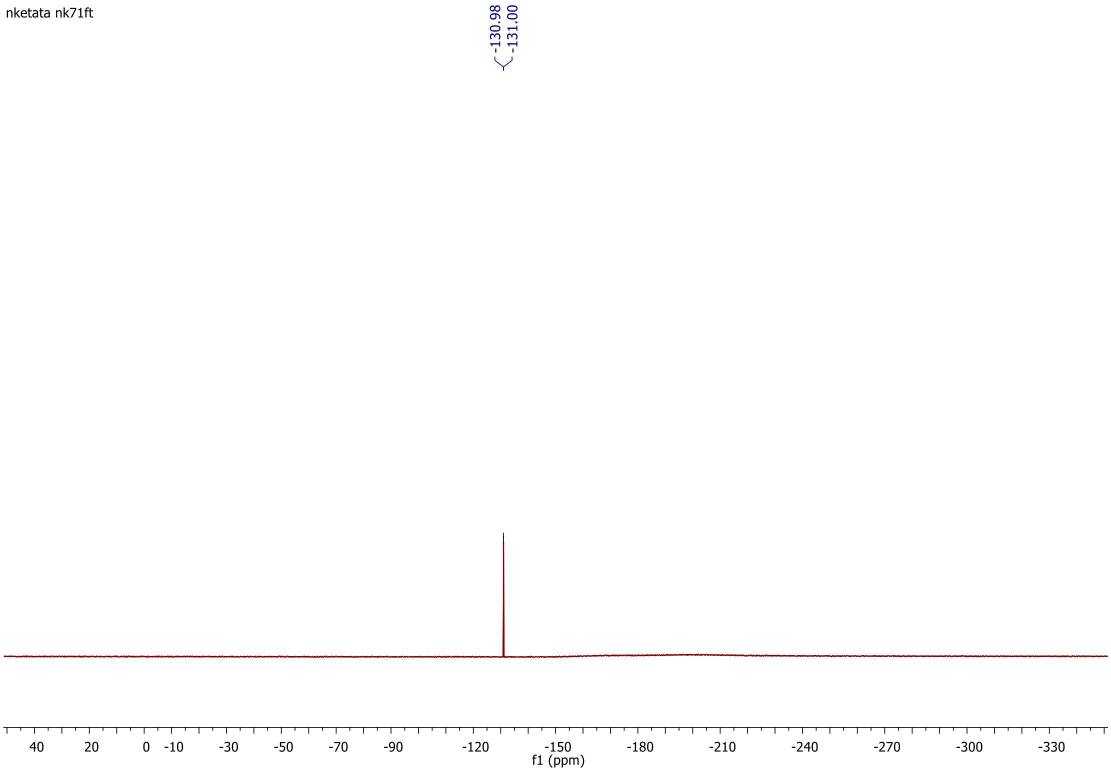

^{13}C NMR (100 MHz, $CDCl_3$)

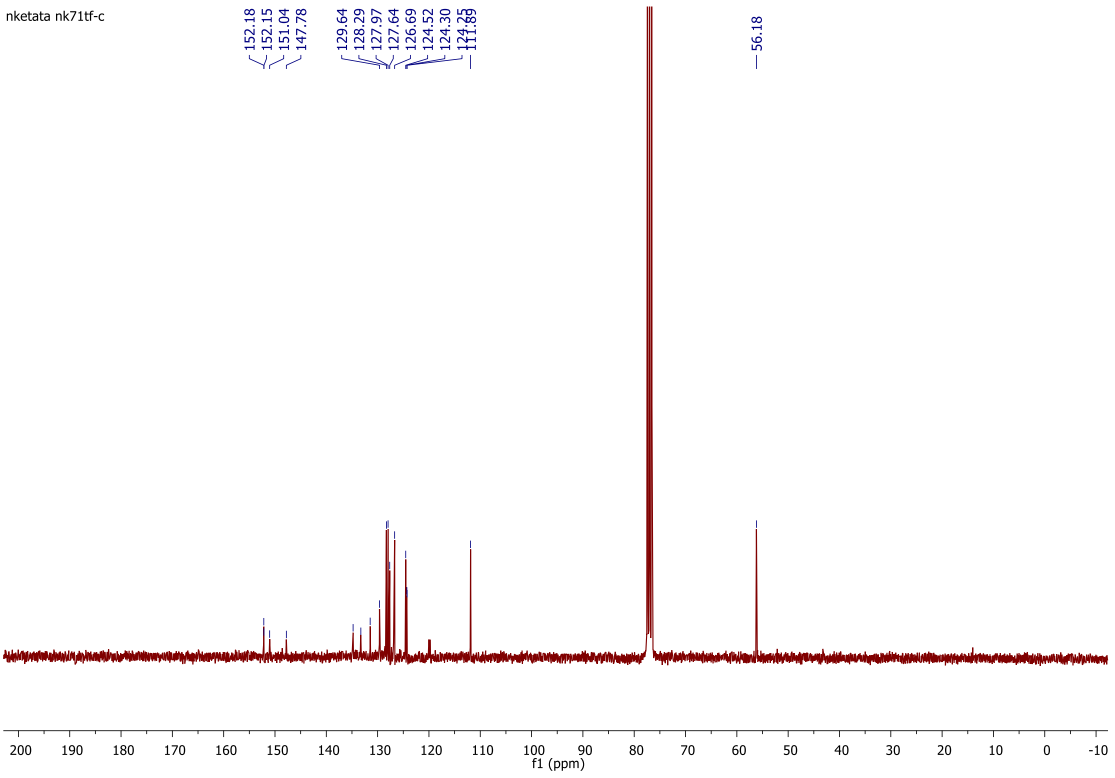

8,10-Dichloro-7-fluorofluoranthene (12)

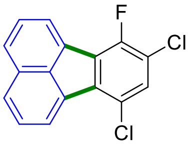

^{1}H NMR (400MHz, $CDCl_3$)

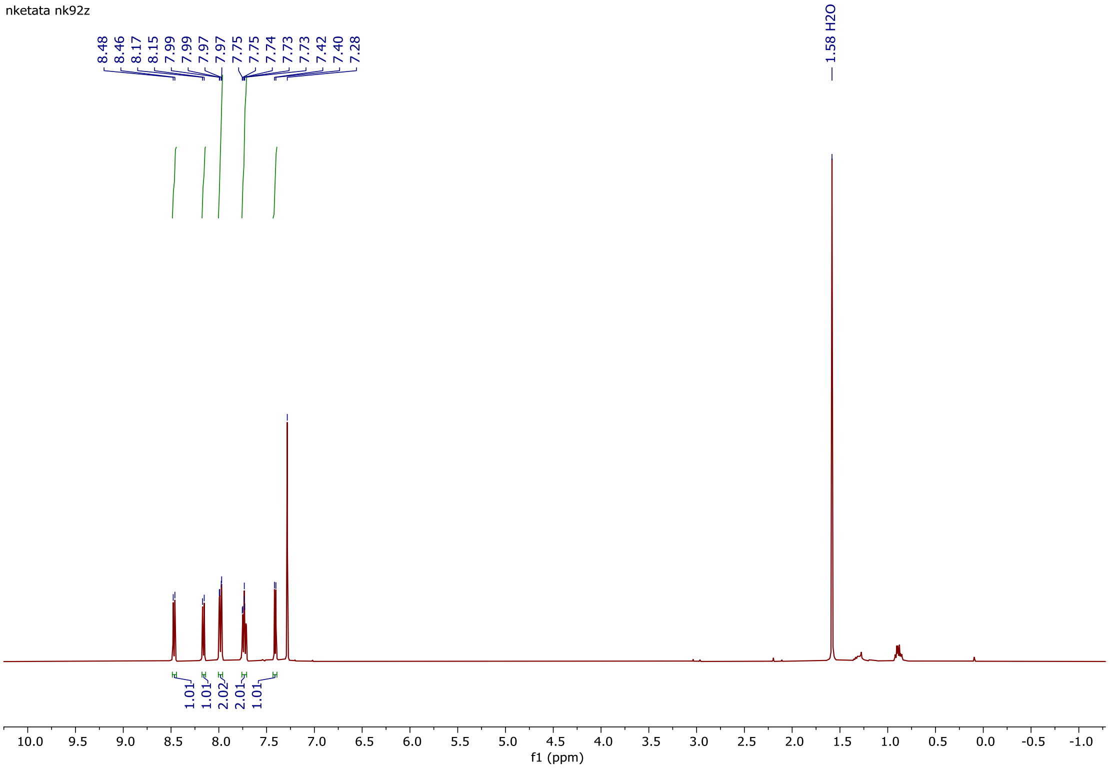

^{19}F NMR (376 MHz, $CDCl_3$)

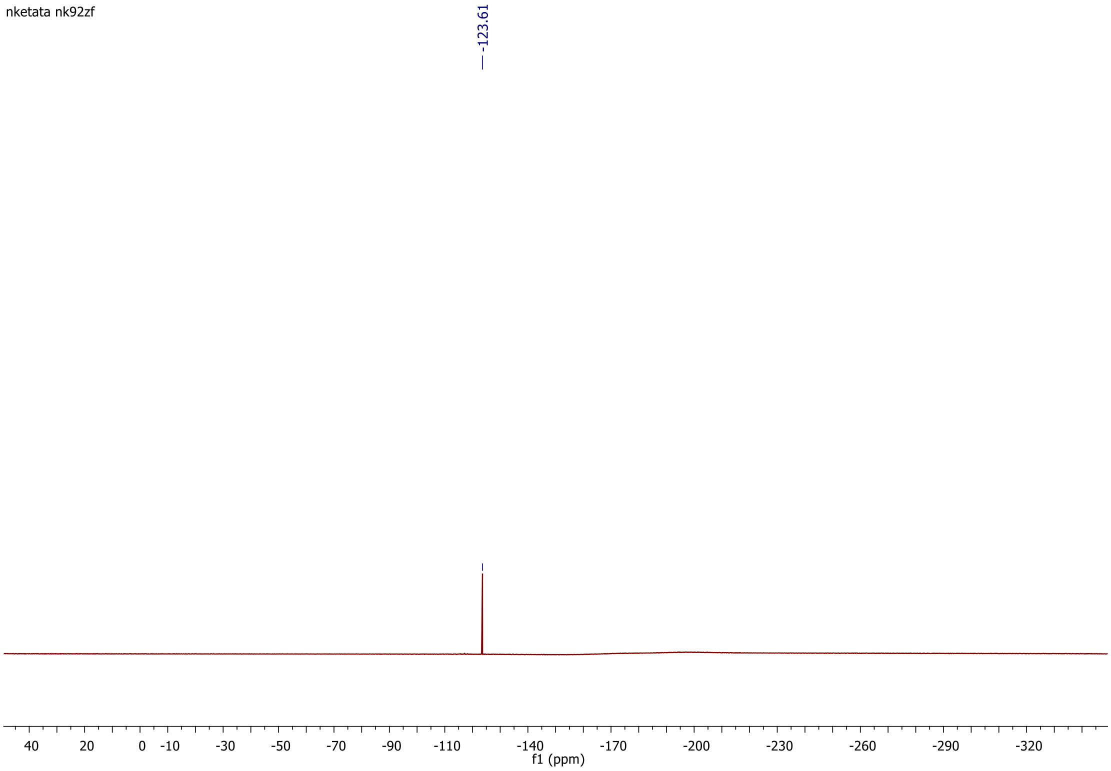

^{13}C NMR (100 MHz, $CDCl_3$)

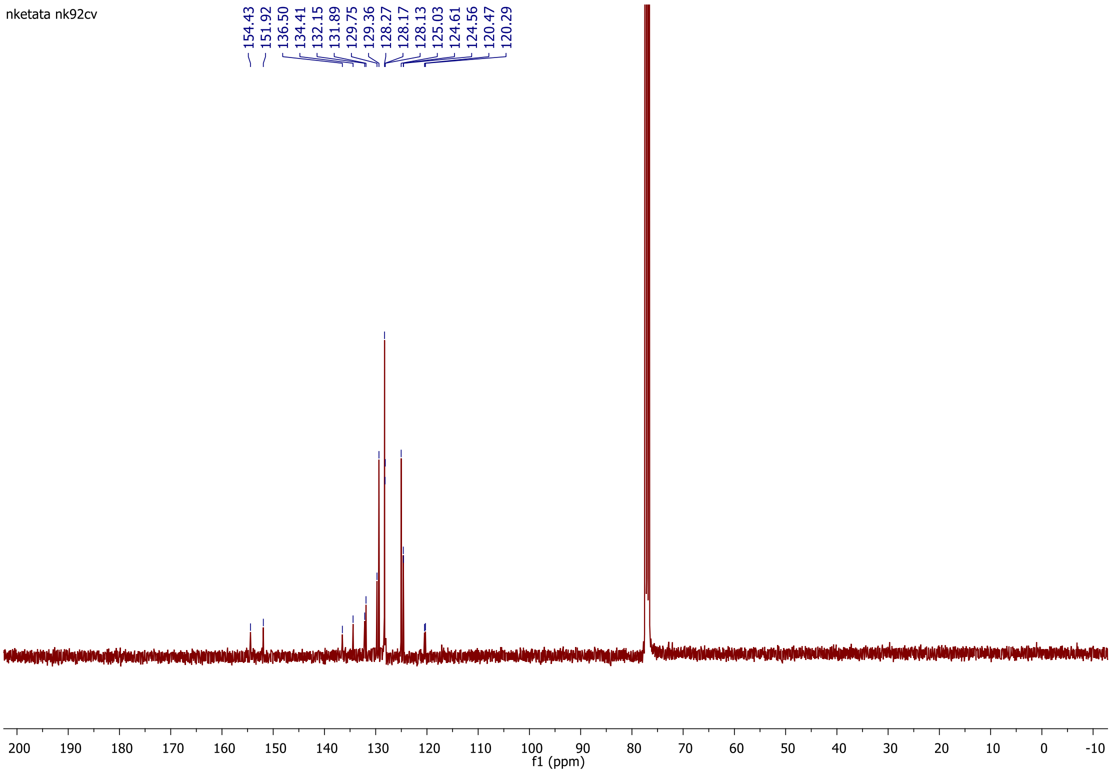

10-Chloro-7-fluoro-8-methylfluoranthene (13)

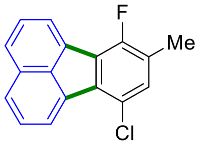

^{1}H NMR (400MHz, $CDCl_3$)

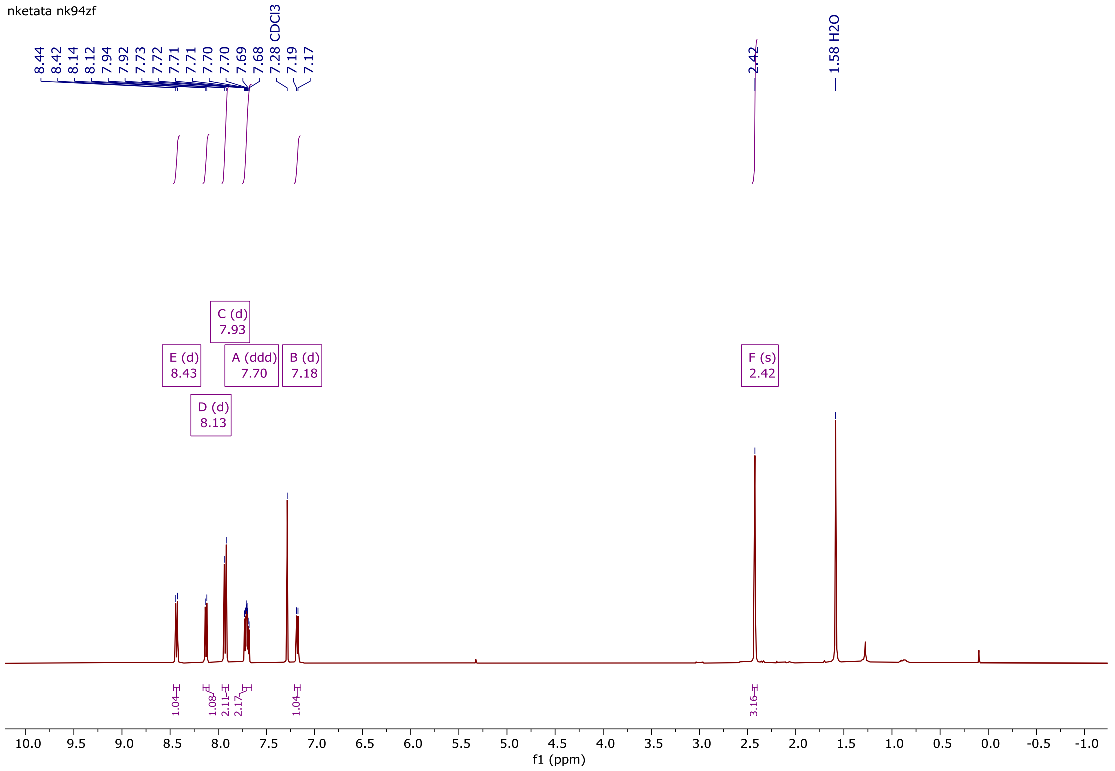

^{19}F NMR (376 MHz, $CDCl_3$)

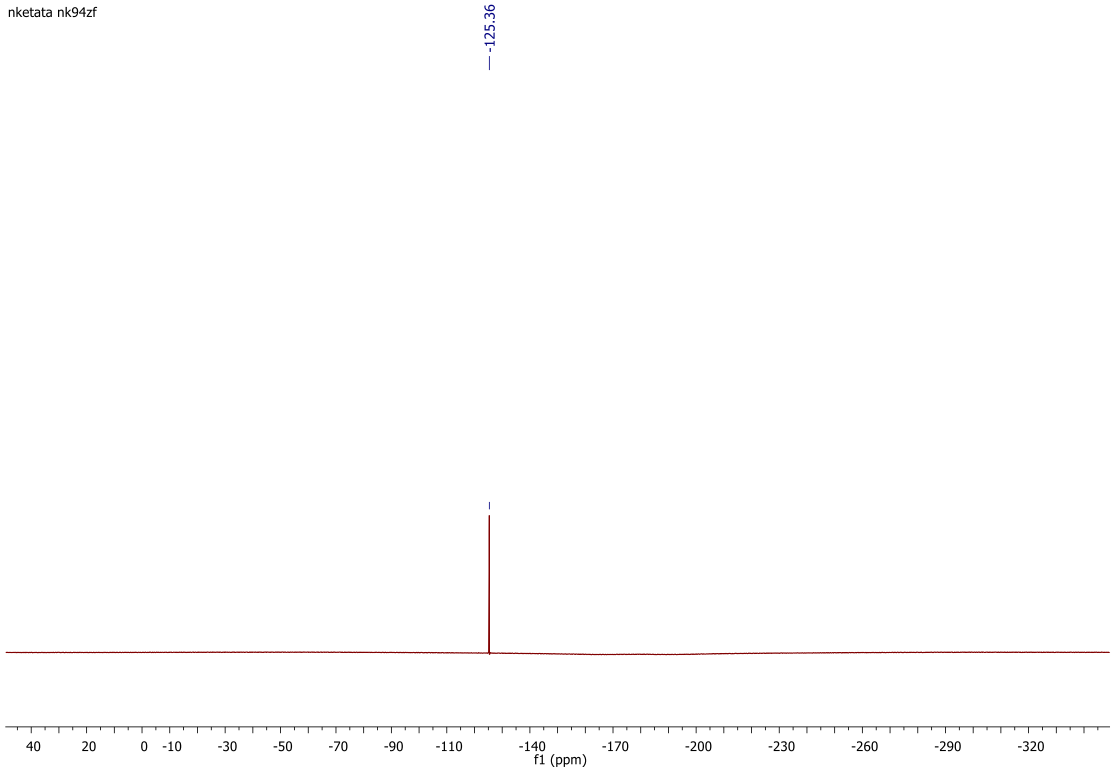

^{13}C NMR (100 MHz, $CDCl_3$)

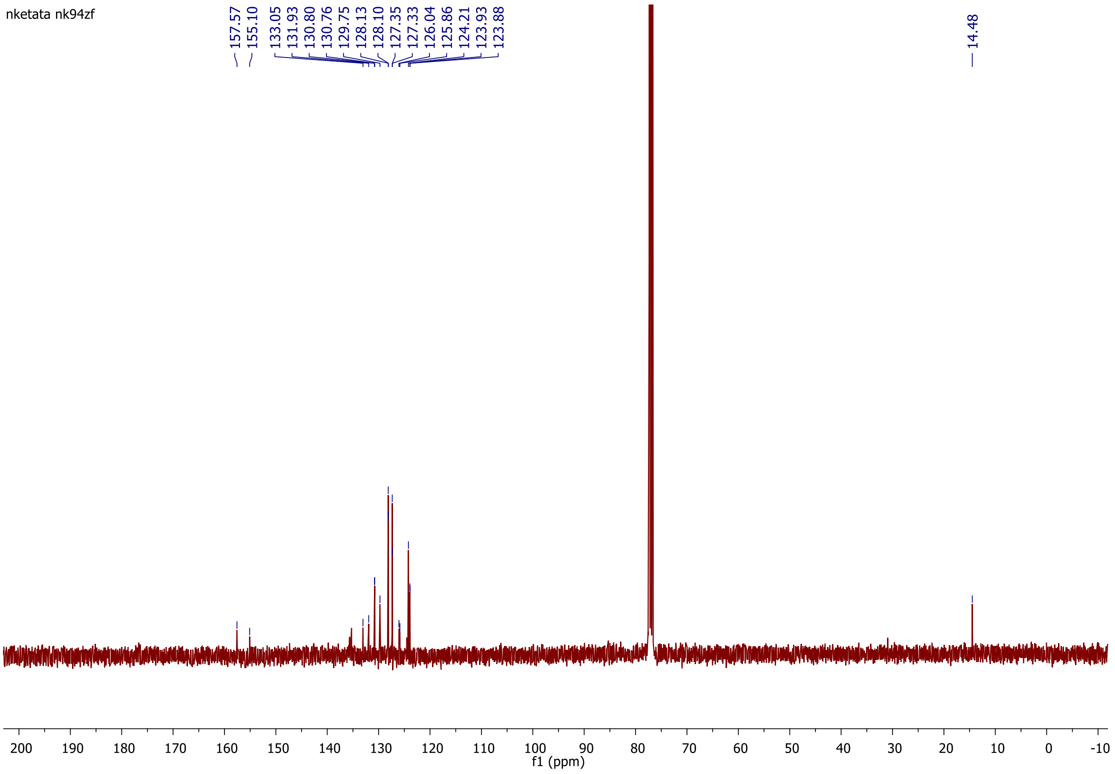

8-Chloro-10-fluoro-7-methylfluoranthene (14)

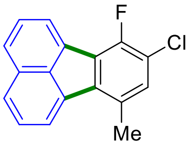

^{1}H NMR (400MHz, $CDCl_3$)

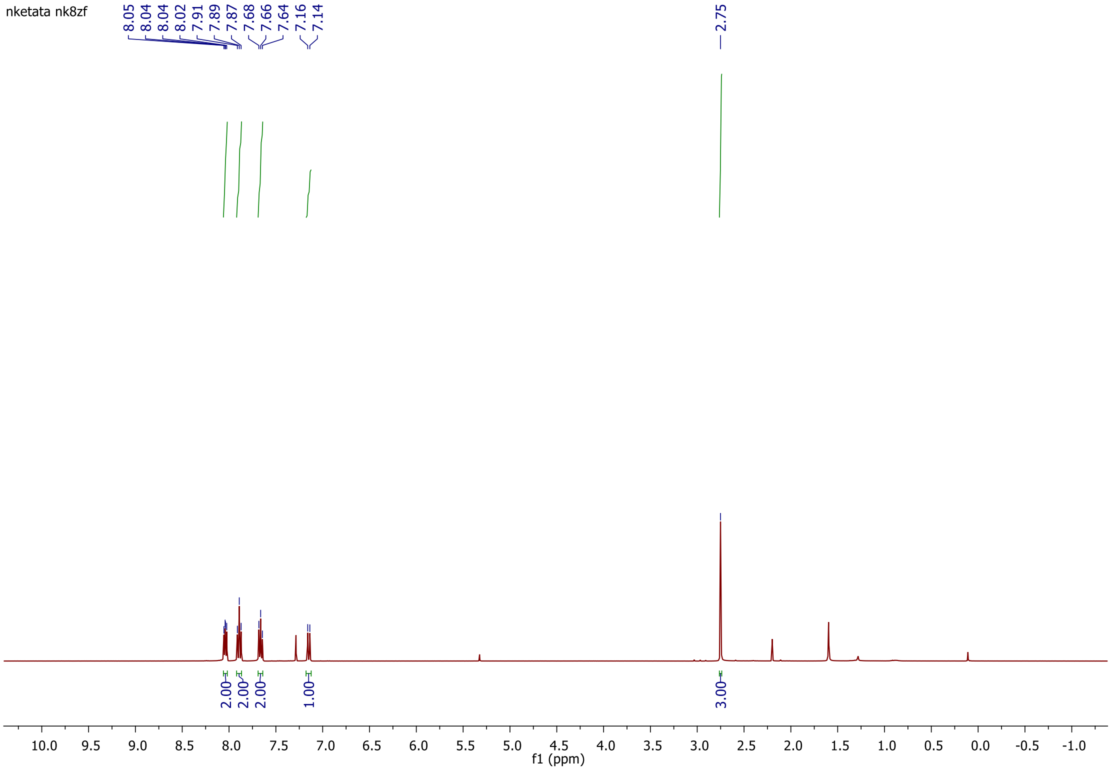

^{19}F NMR (376 MHz, $CDCl_3$)

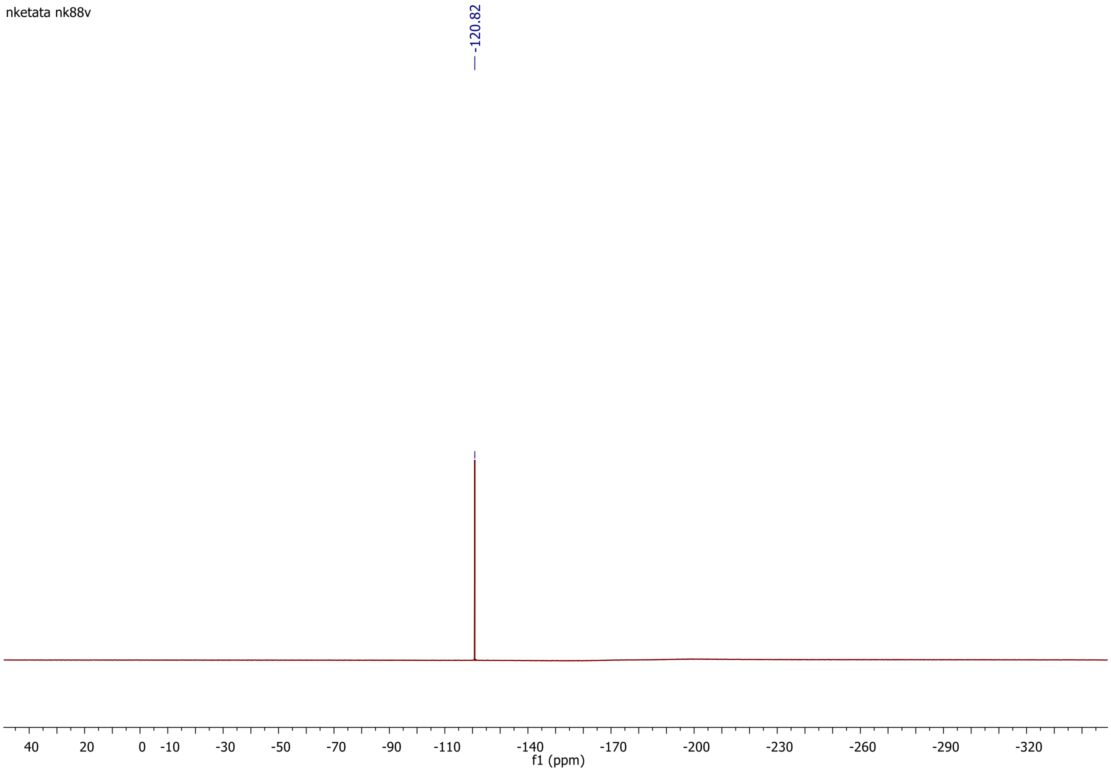

^{13}C NMR (100 MHz, $CDCl_3$)

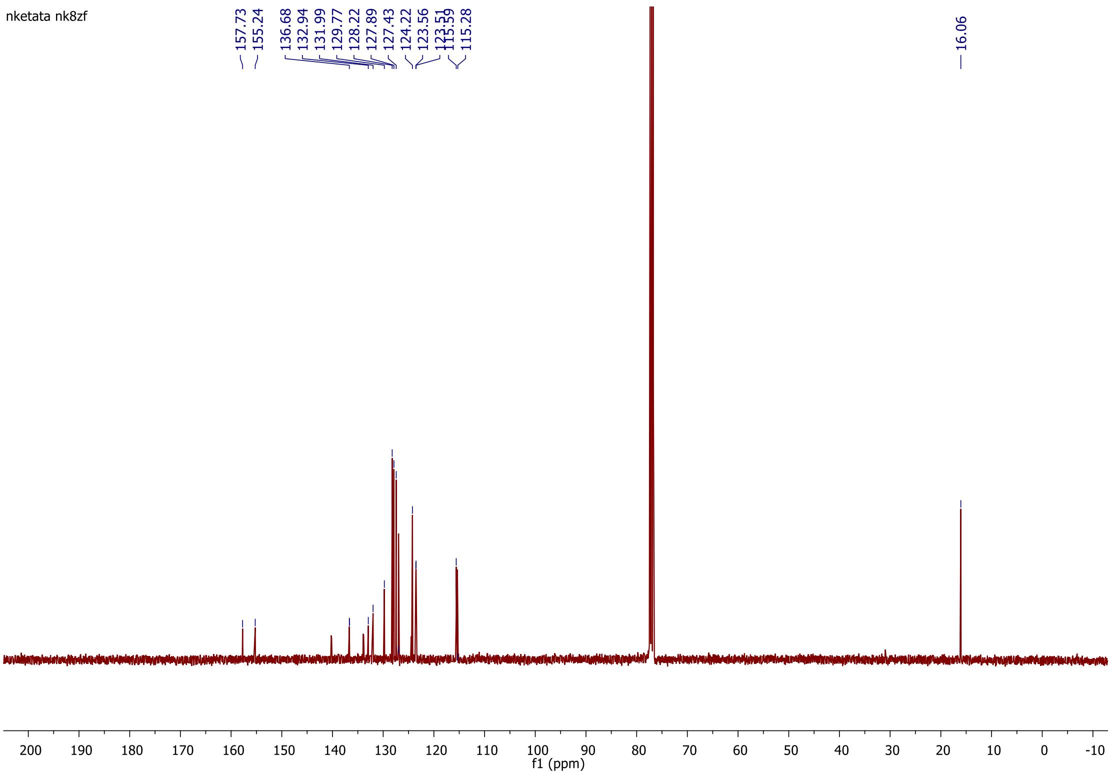

7,9-Dichlorofluoranthene (15)

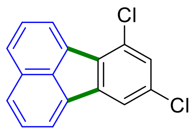

^{1}H NMR (400MHz, $CDCl_3$)

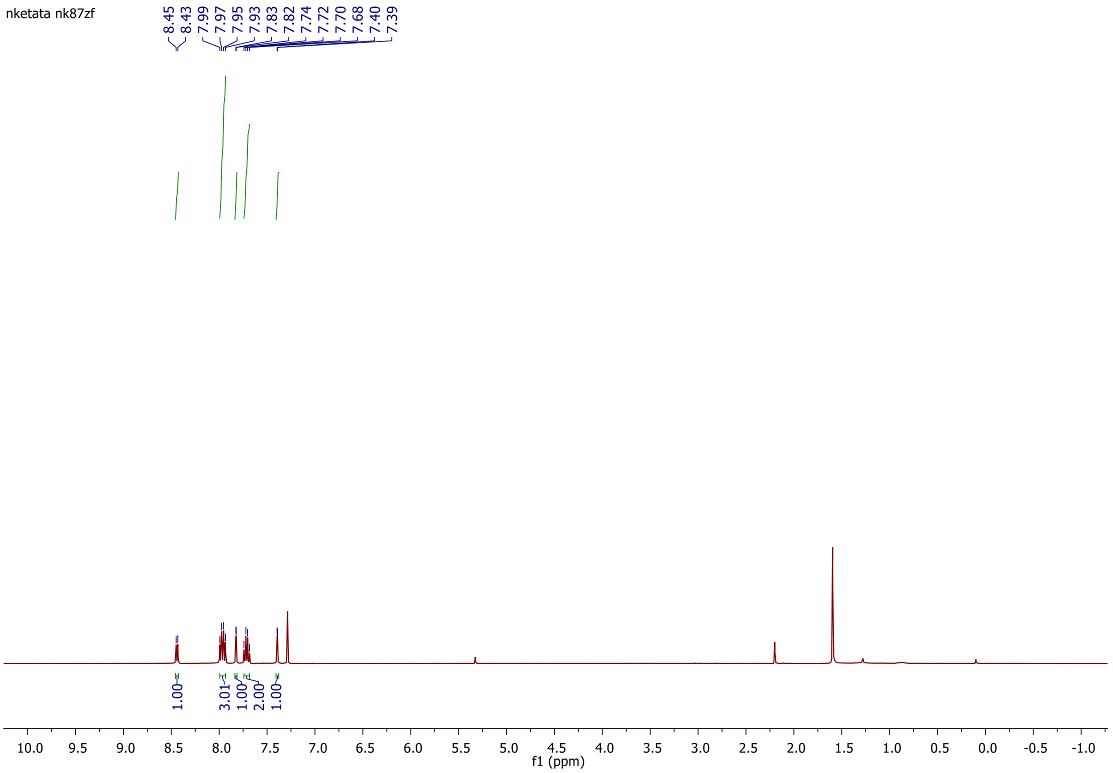

^{13}C NMR (100 MHz, $CDCl_3$)

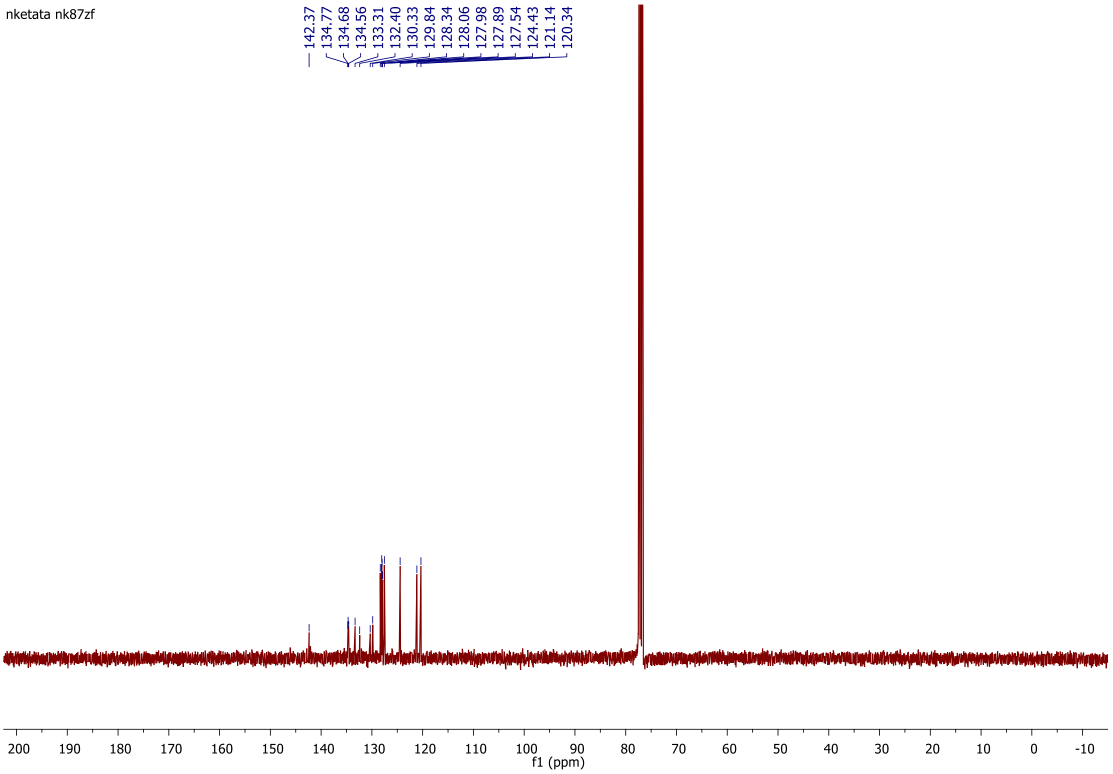

7,9-Dimethylacenaphtho[1,2-*c*]thiophene (16)

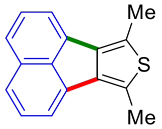

^{1}H NMR (400MHz, $CDCl_3$)

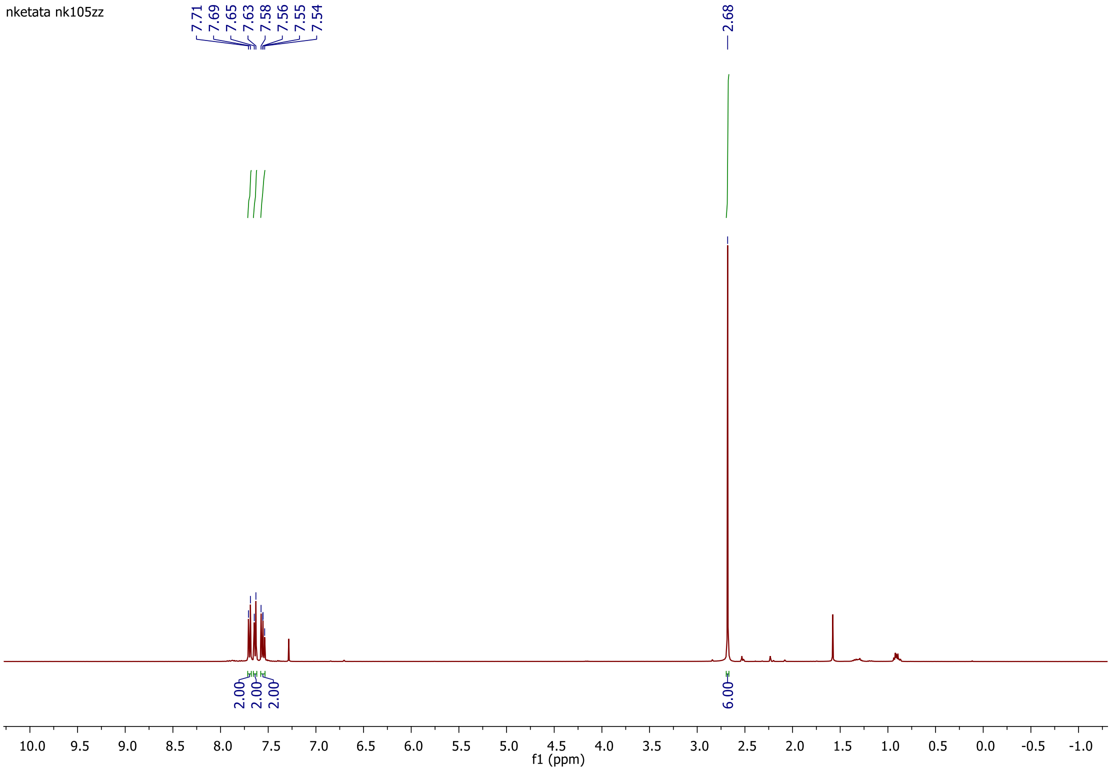

^{13}C NMR (100 MHz, $CDCl_3$)

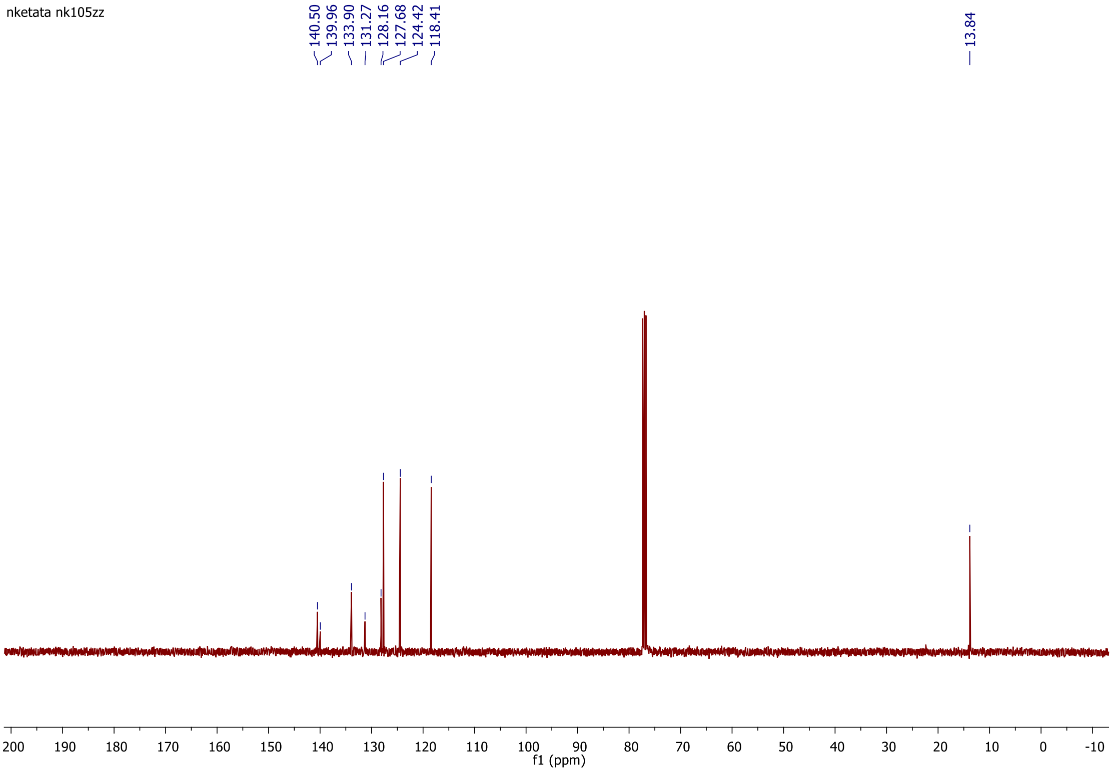

7,8,9-Trimethyl-8H-acenaphtho[1,2-*c*]pyrrole (17)

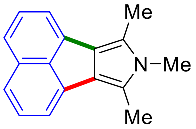

^{1}H NMR (400MHz, $CDCl_3$)

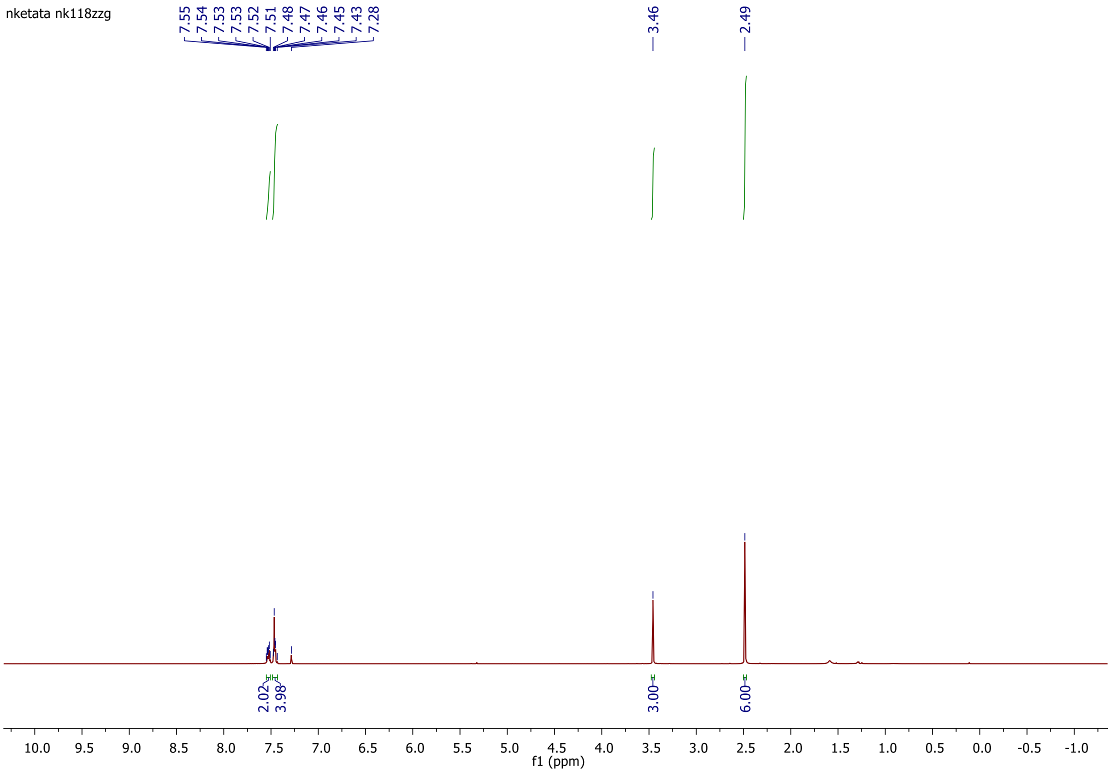

^{13}C NMR (100 MHz, $CDCl_3$)

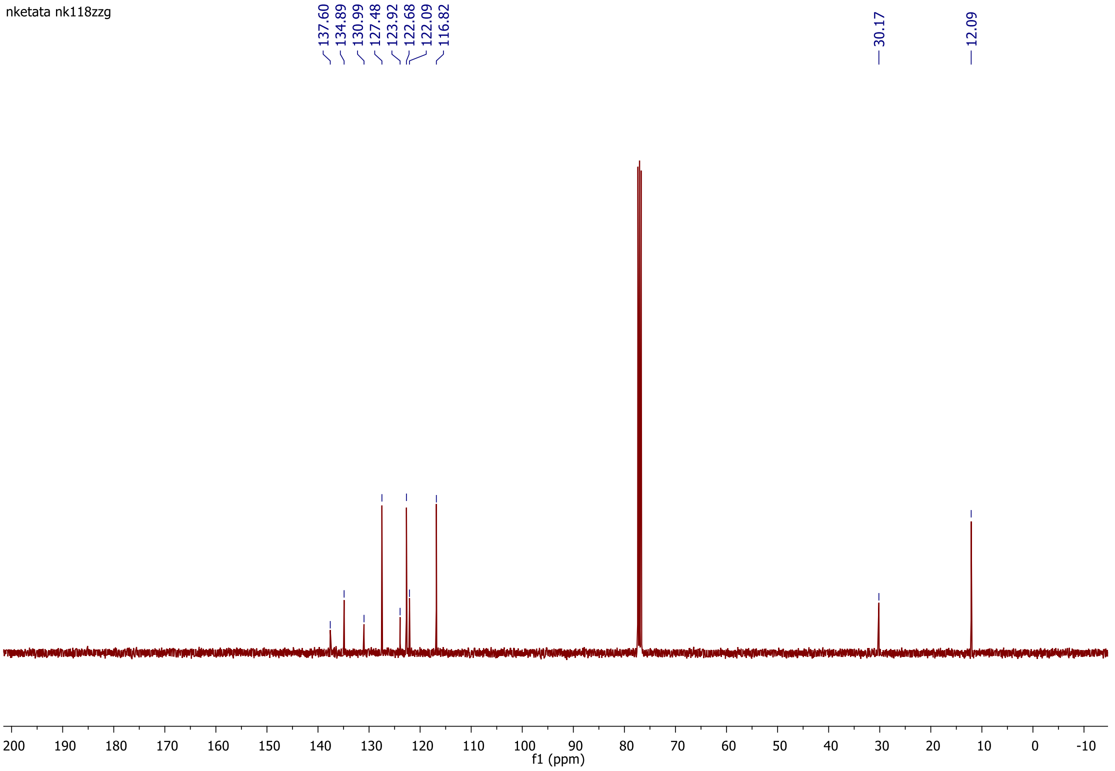

1-(9-Methylacenaphtho[1,2-*c*]furan-7-yl)ethan-1-one (18)

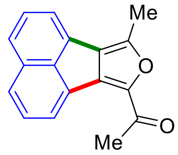

^{1}H NMR (400MHz, $CDCl_3$)

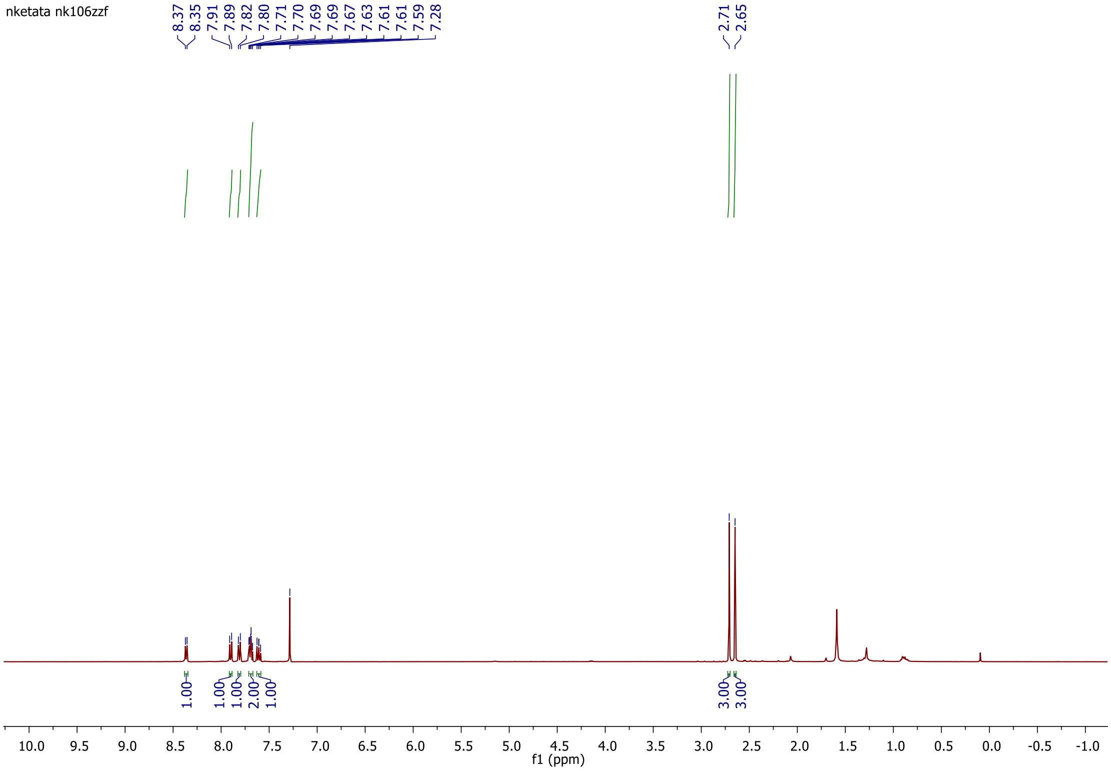

^{13}C NMR (100 MHz, $CDCl_3$)

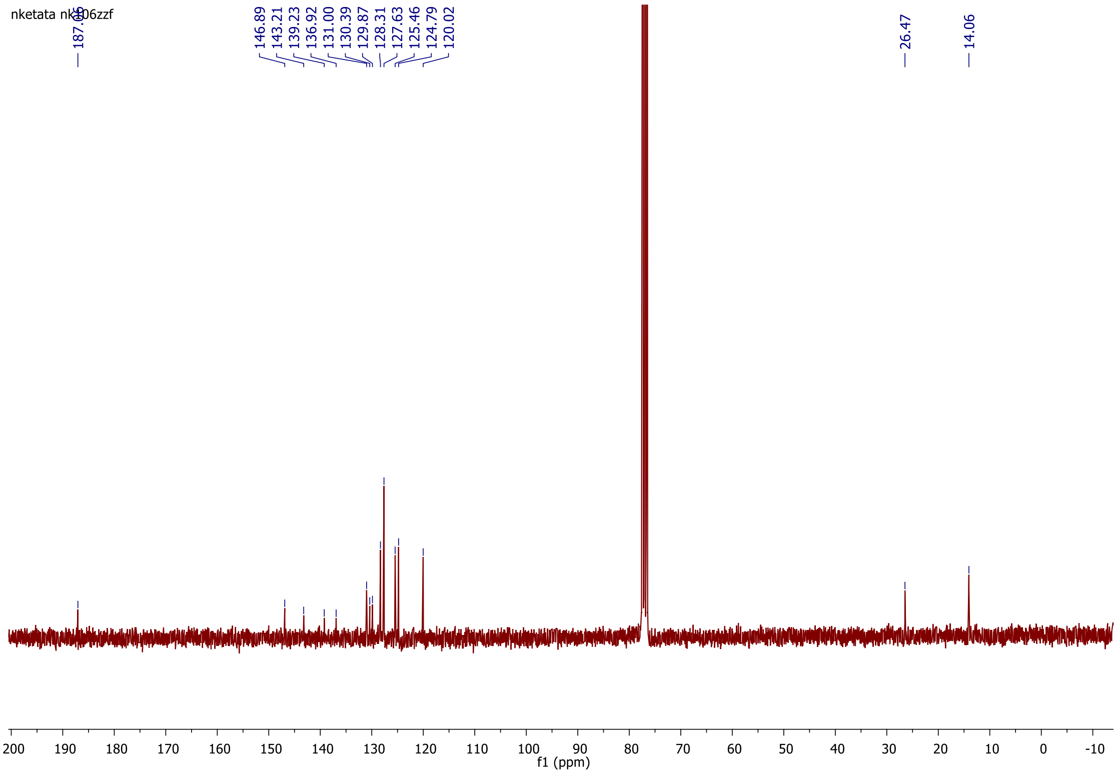

7-Ethoxyfluoranthene (19)

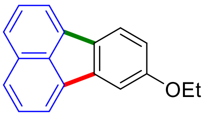

^{1}H NMR (400MHz, $CDCl_3$)

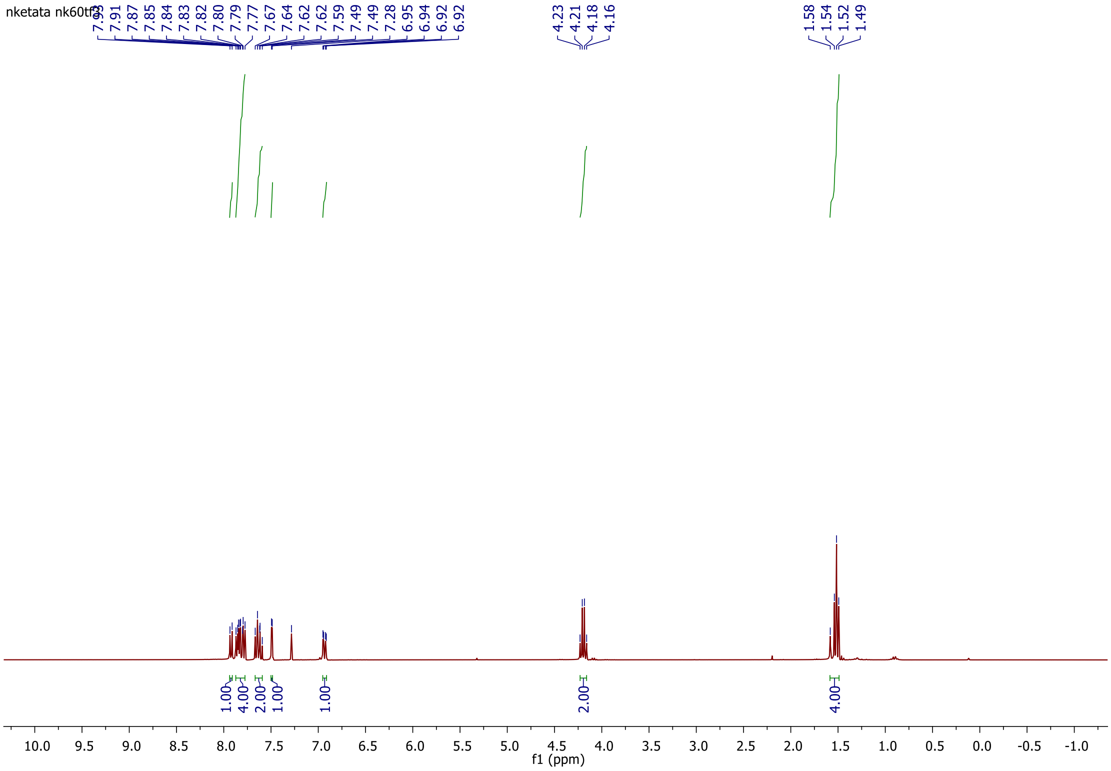

^{13}C NMR (100 MHz, $CDCl_3$)

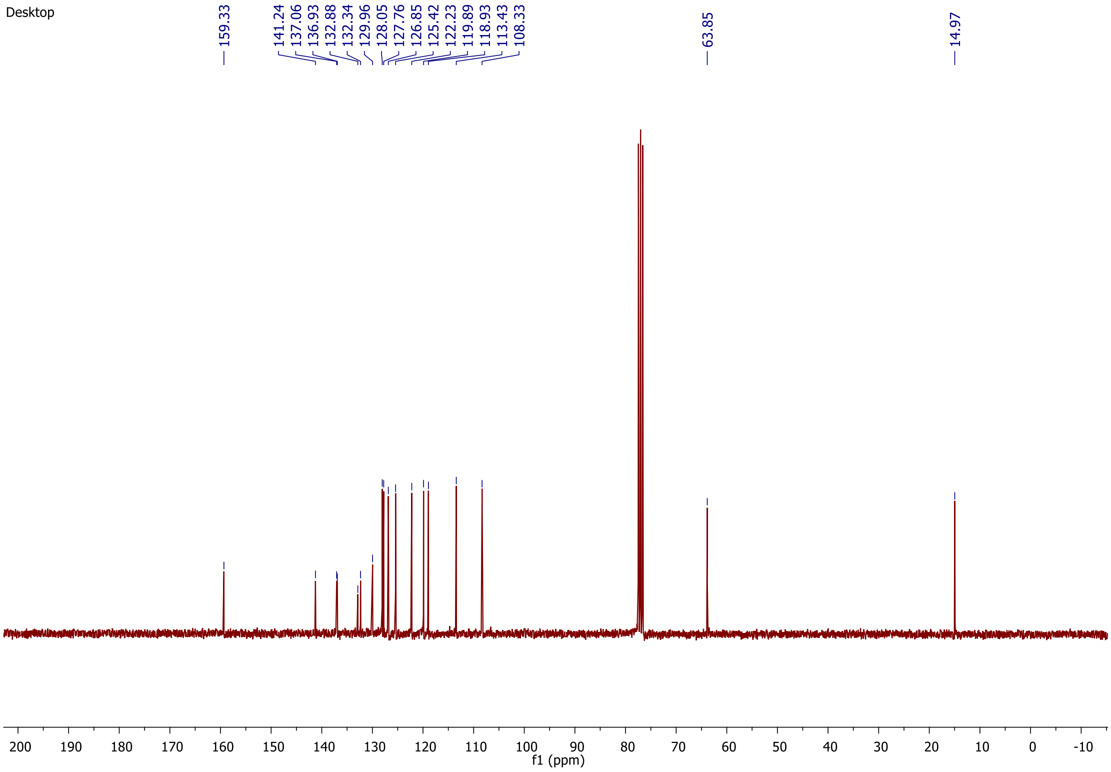

8-Methylfluoranthene (20)

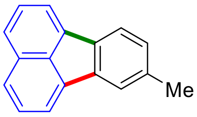

^{1}H NMR (400MHz, $CDCl_3$)

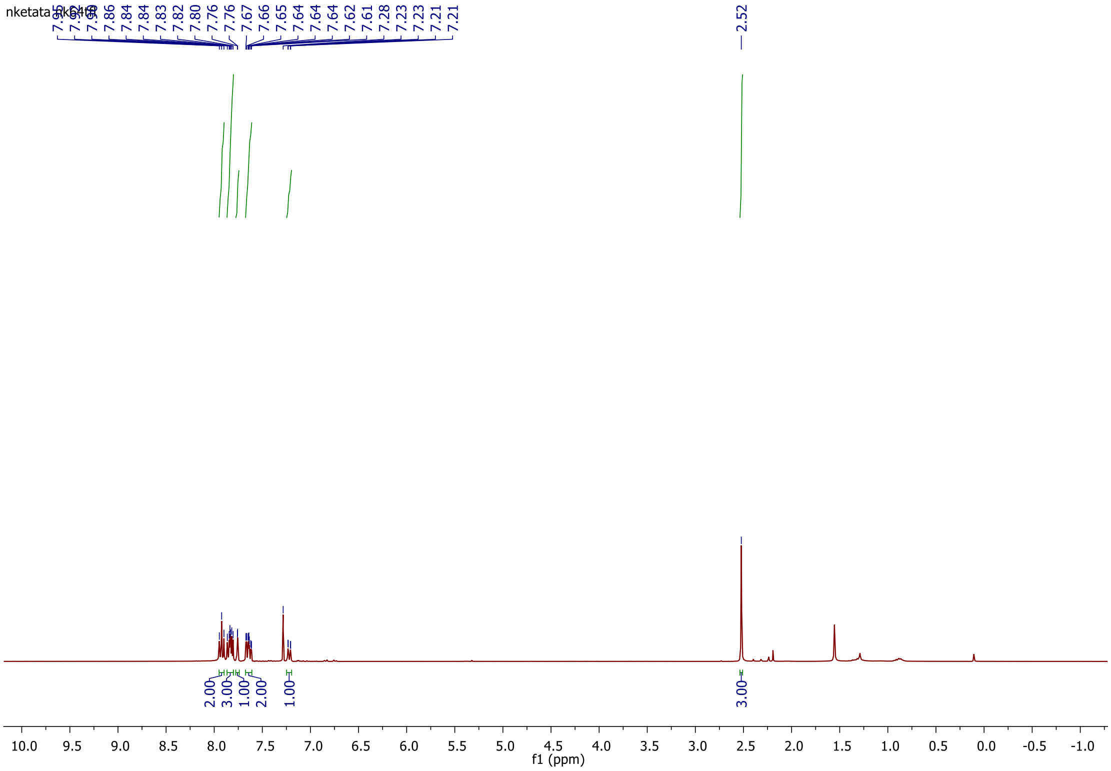

^{13}C NMR (100 MHz, $CDCl_3$)

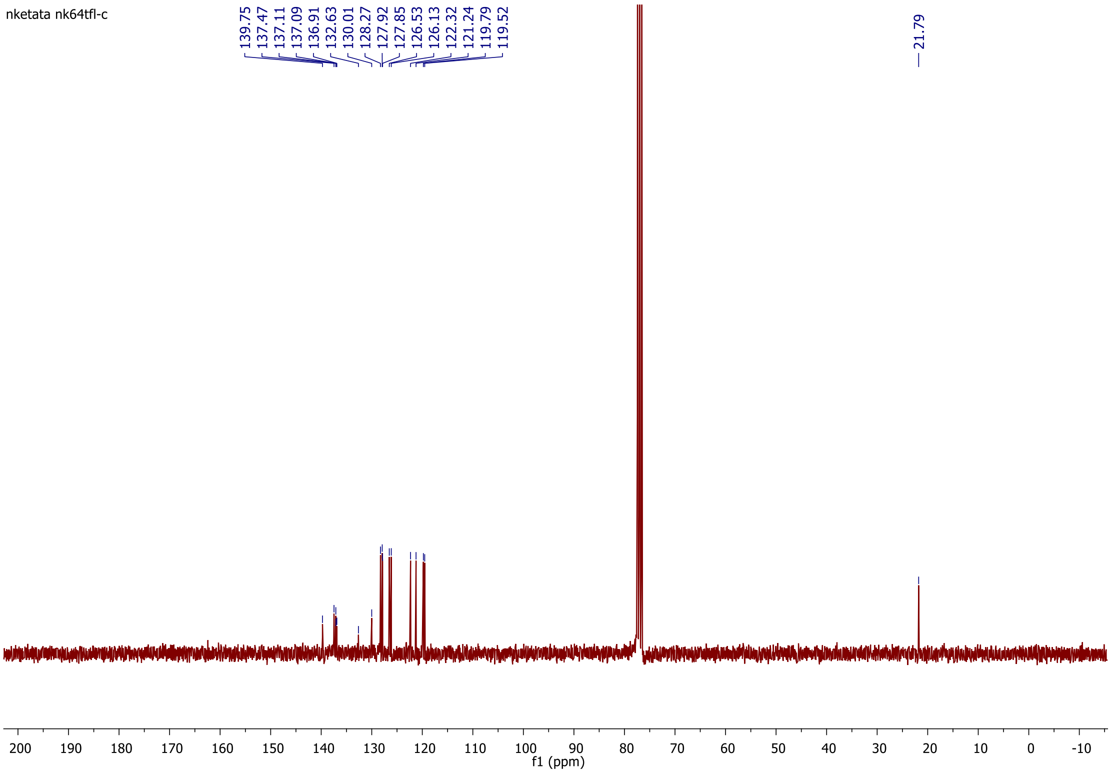

8-Chlorofluoranthene (21)

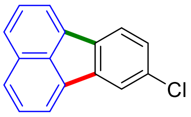

^{1}H NMR (400MHz, $CDCl_3$)

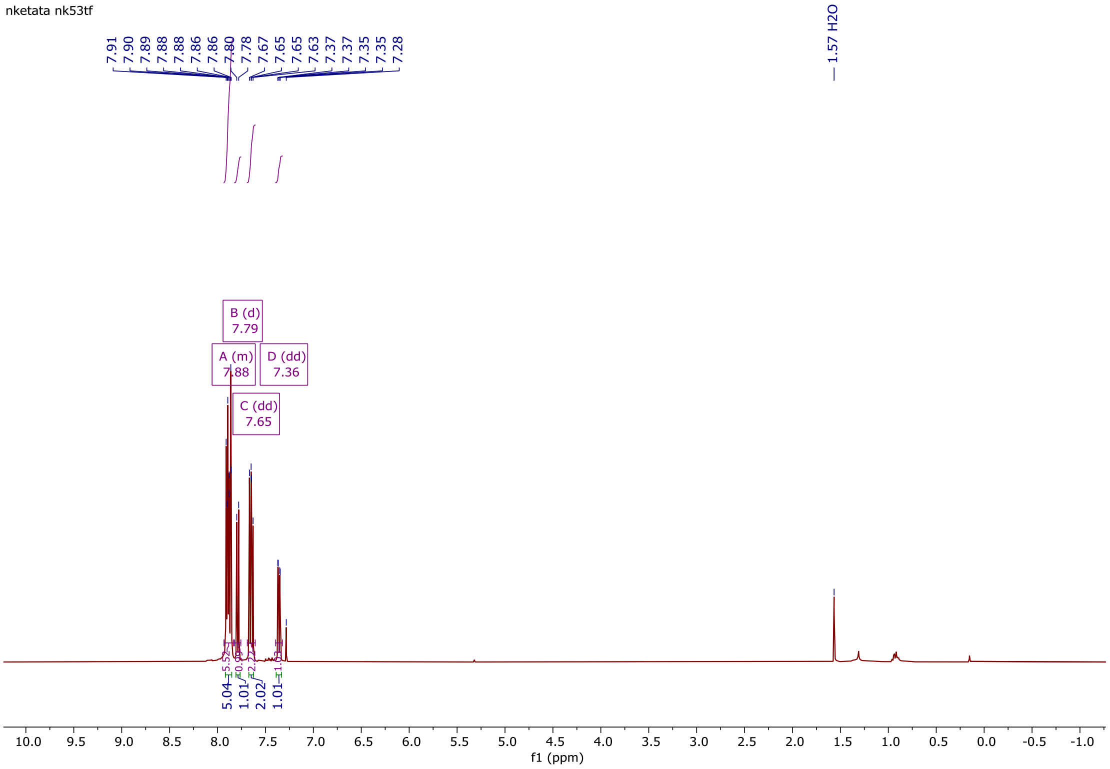

^{13}C NMR (100 MHz, $CDCl_3$)

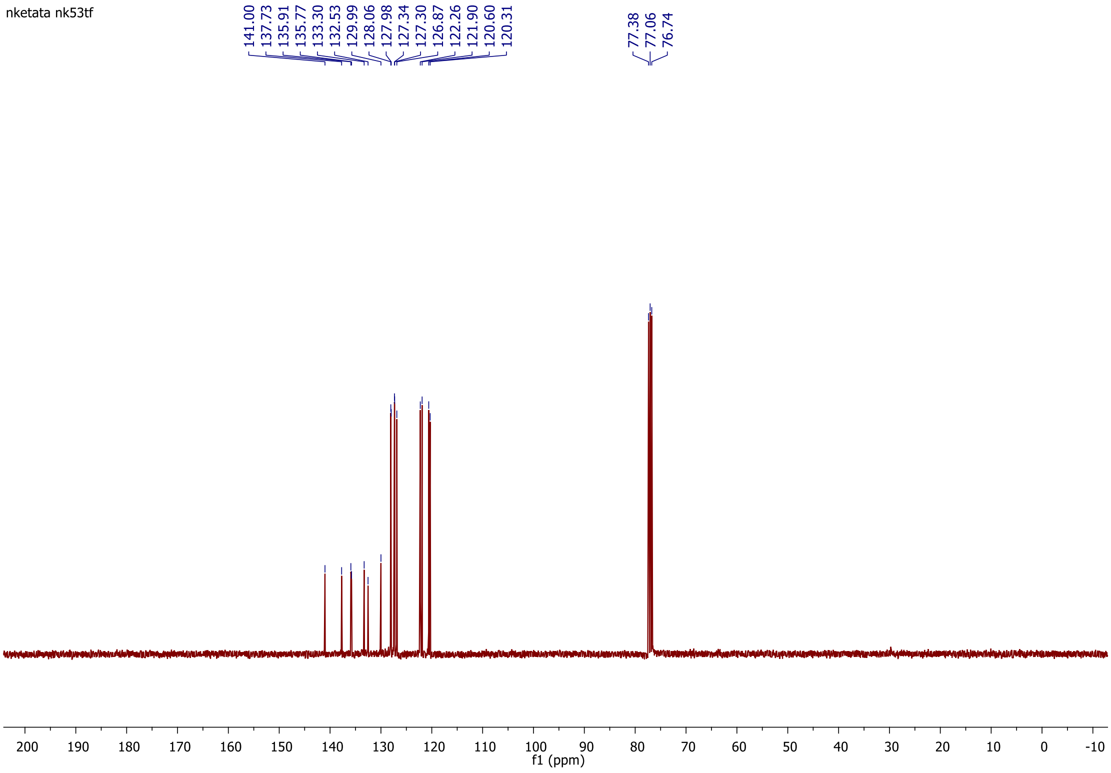

Methyl fluoranthene-8-carboxylate (22)

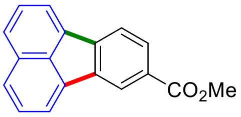

^{1}H NMR (400MHz, $CDCl_3$)

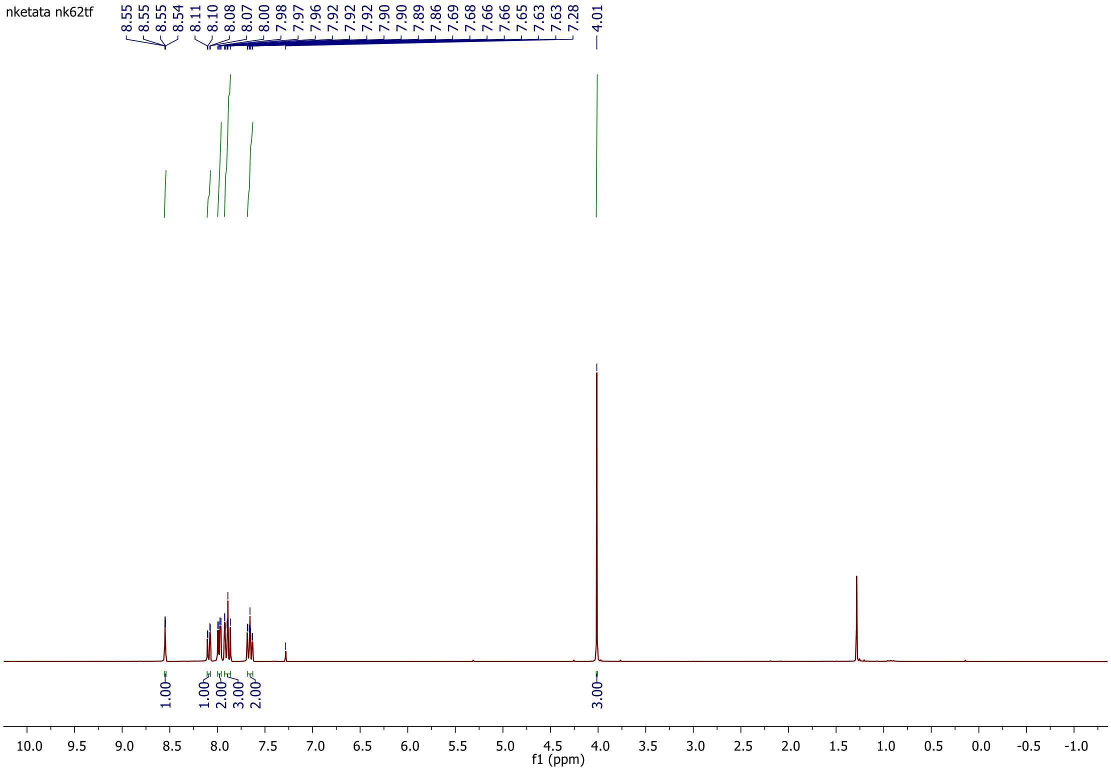

^{13}C NMR (100 MHz, $CDCl_3$)

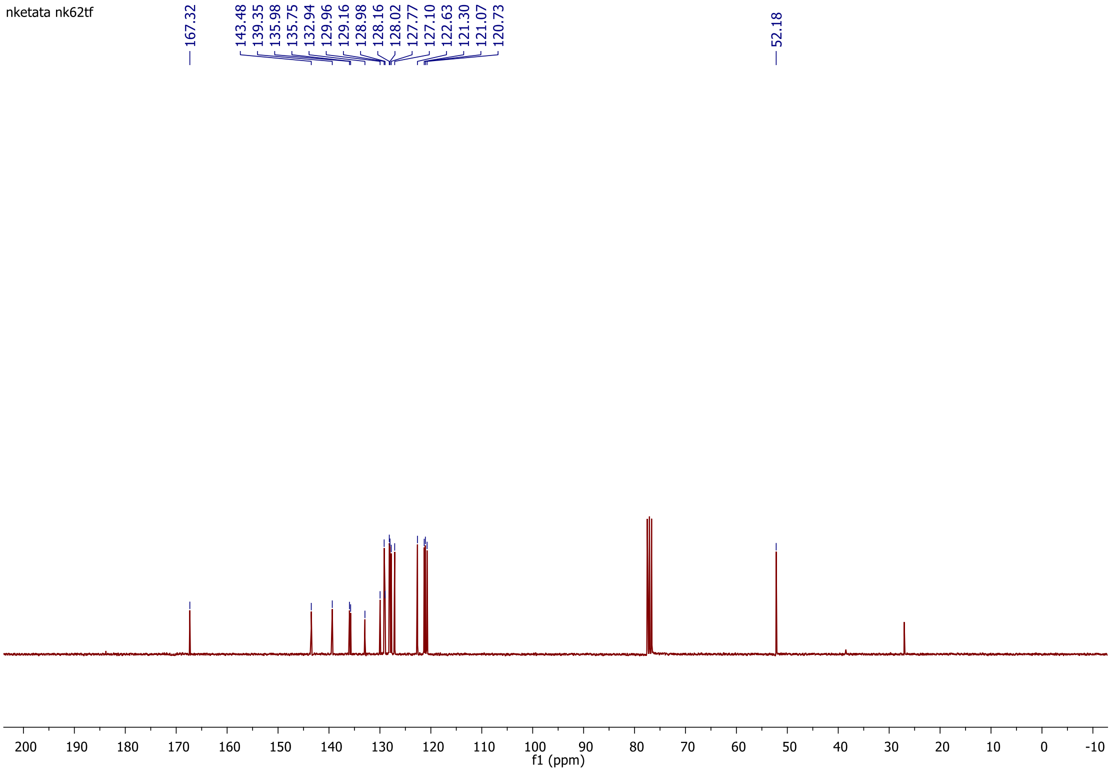

Fluoranthene-8-carbonitrile (23)

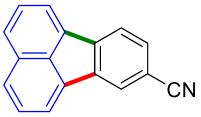

^{1}H NMR (400MHz, $CDCl_3$)

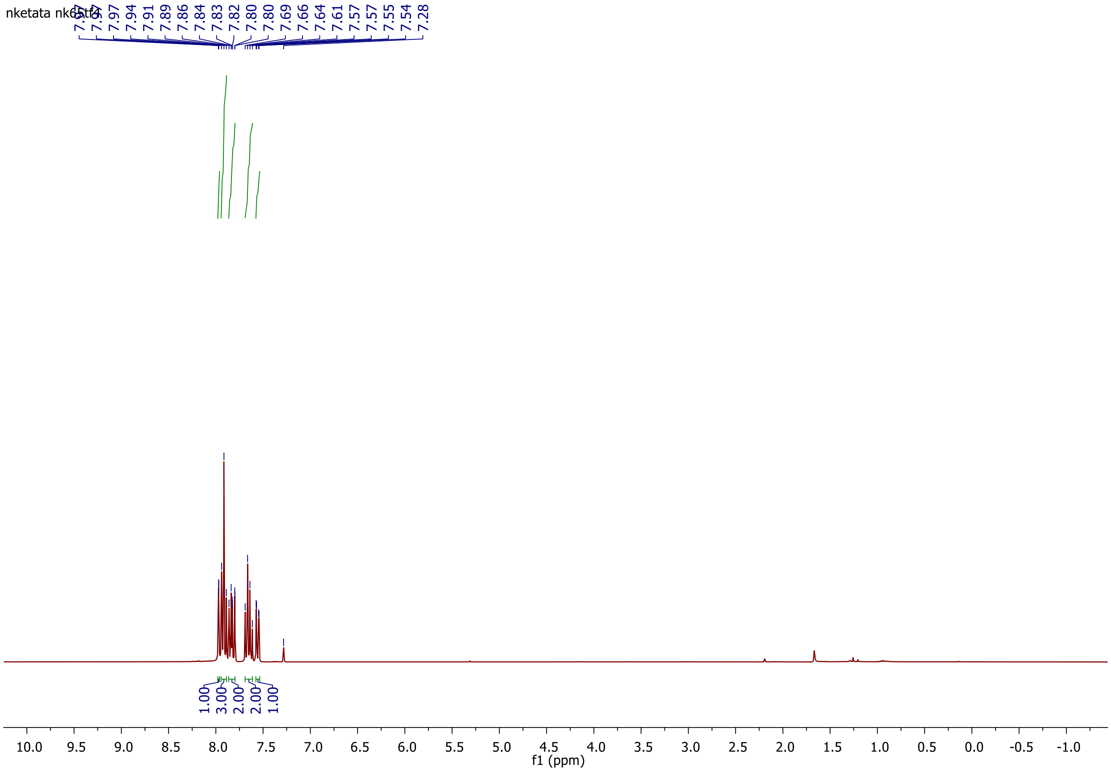

^{13}C NMR (100 MHz, $CDCl_3$)

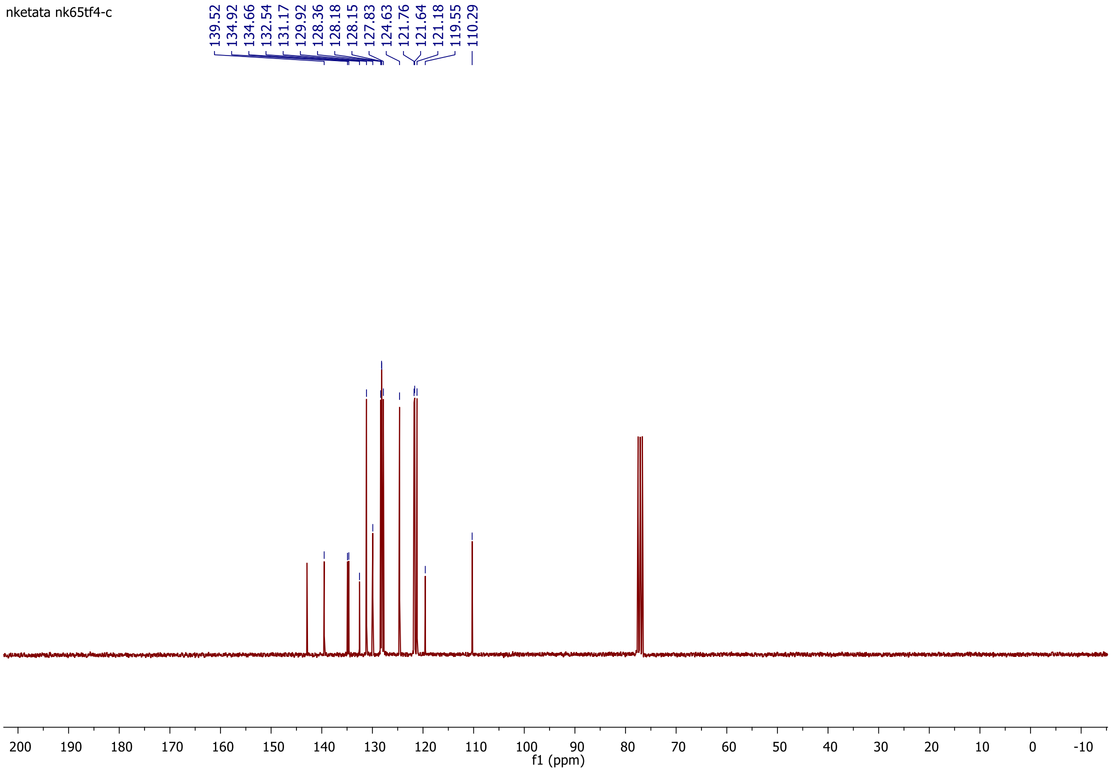

8-Nitrofluoranthene (24)

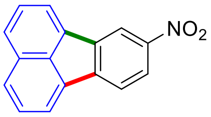

^{1}H NMR (400MHz, $CDCl_3$)

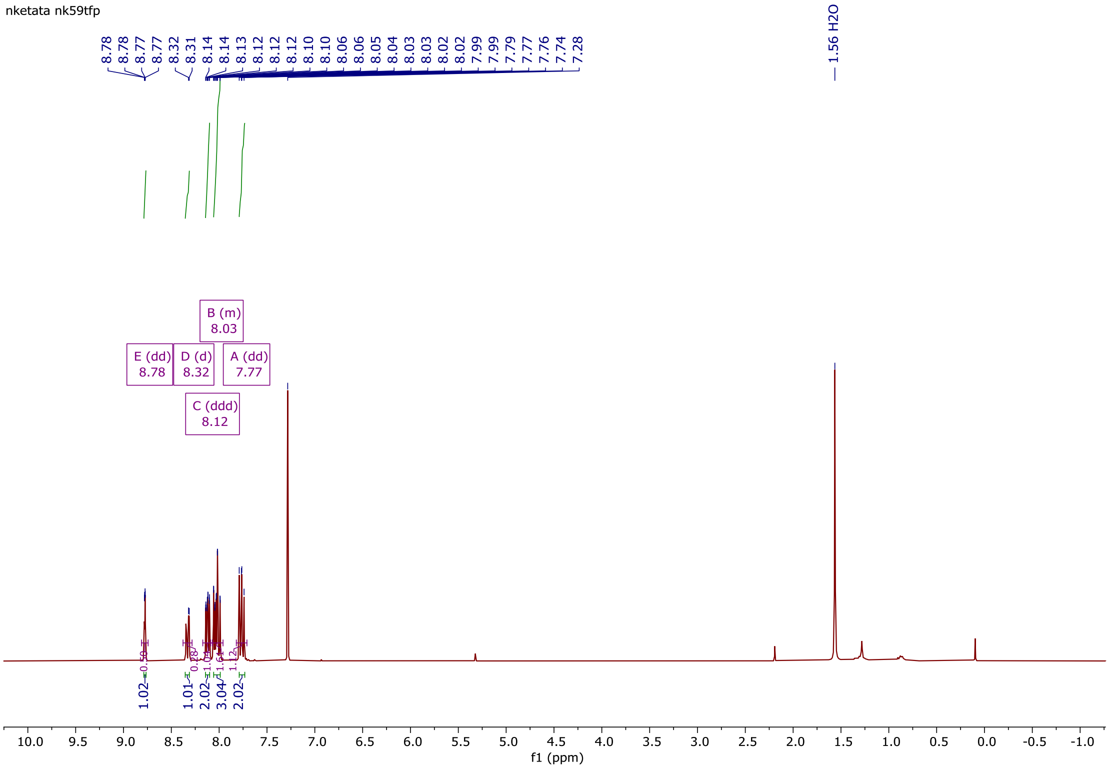

^{13}C NMR (100 MHz, $CDCl_3$)

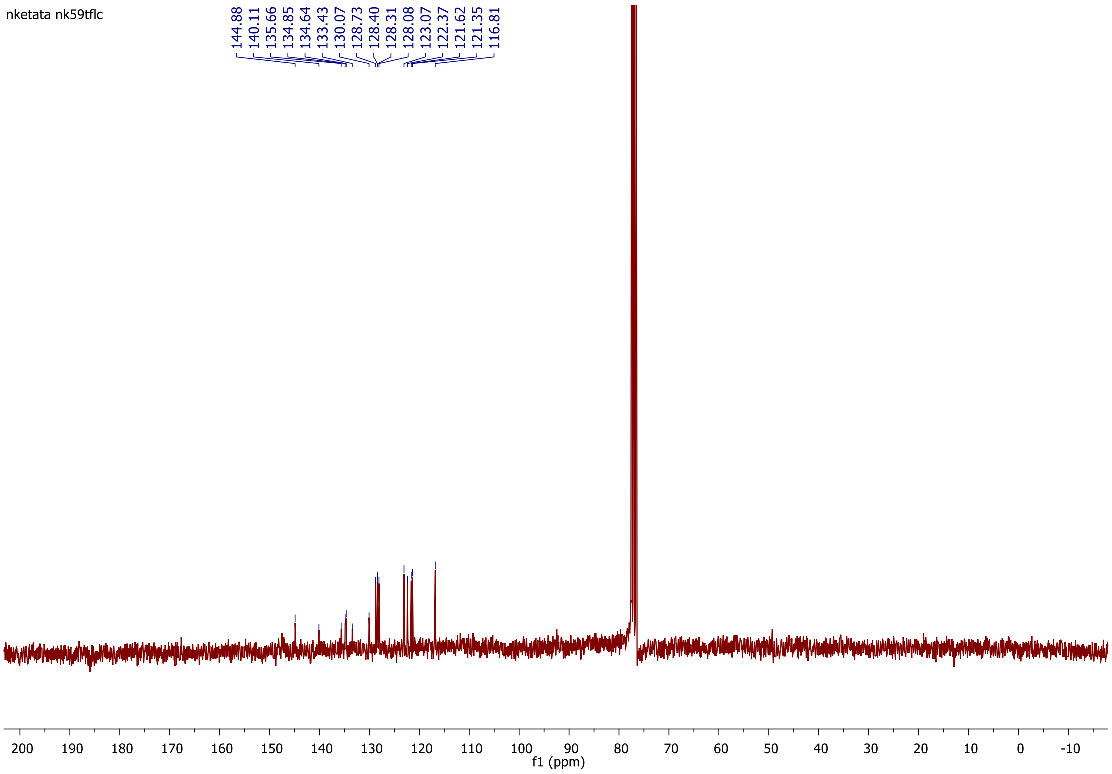

8,9-Difluorofluoranthene (25)

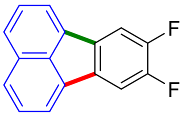

^{1}H NMR (400MHz, $CDCl_3$)

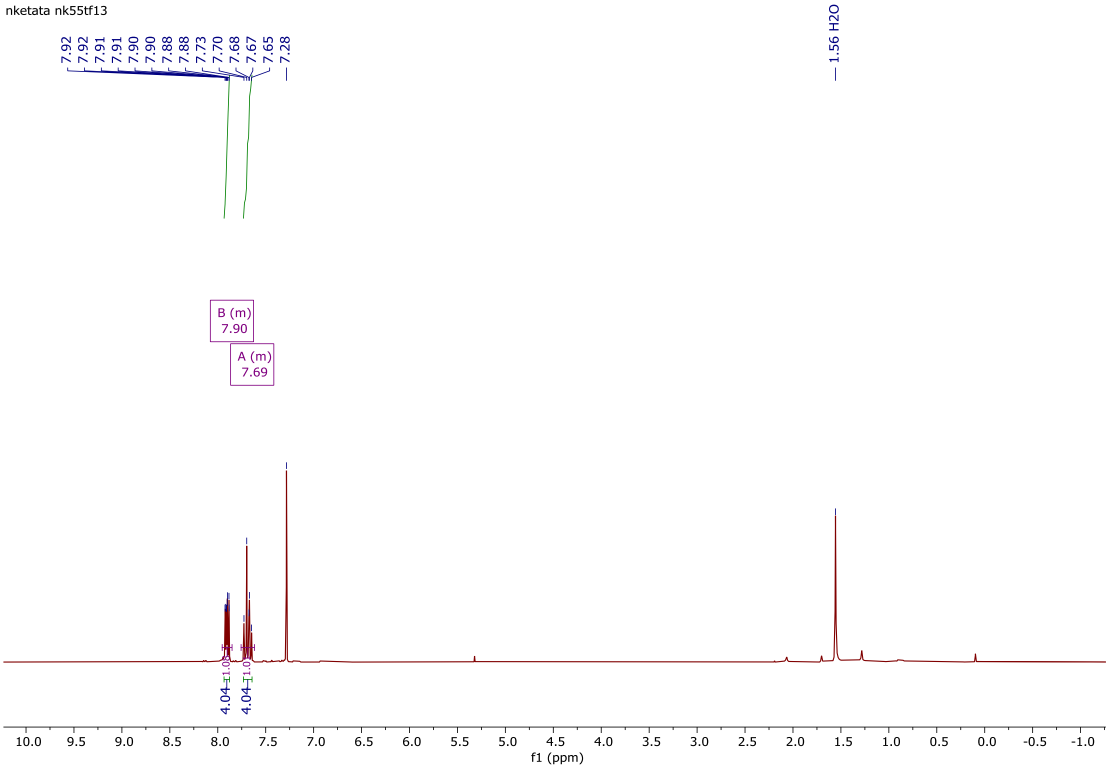

^{19}F NMR (376 MHz, $CDCl_3$)

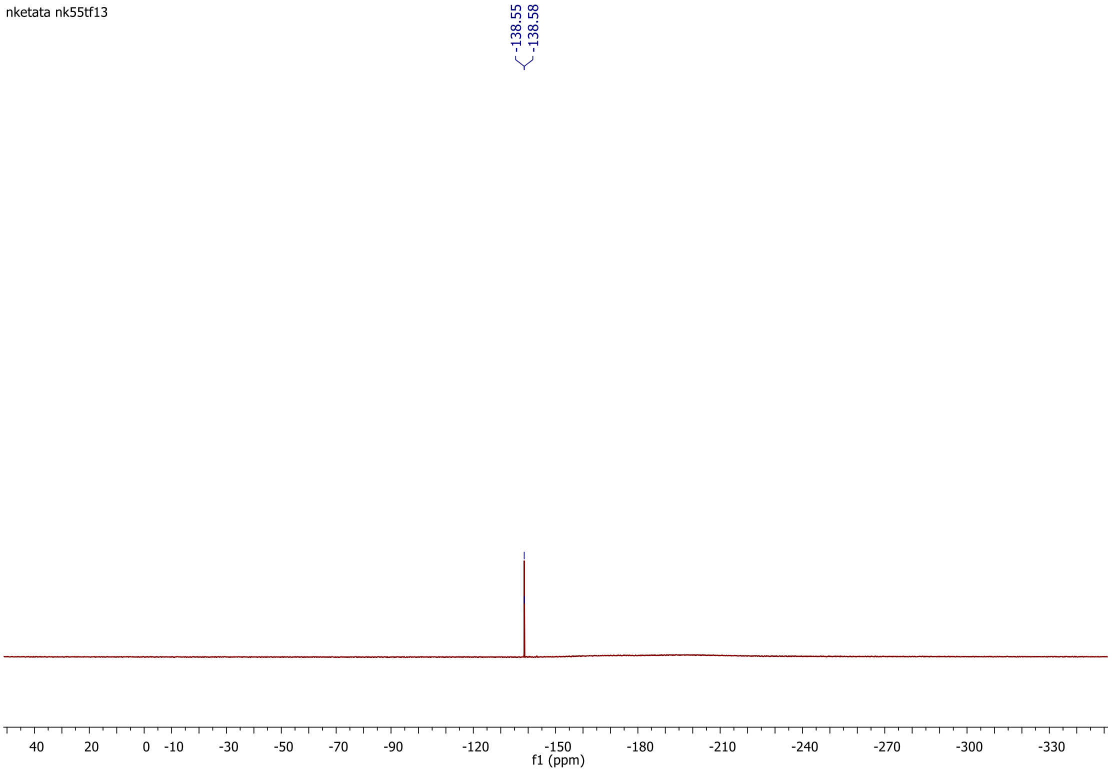

^{13}C NMR (100 MHz, $CDCl_3$)

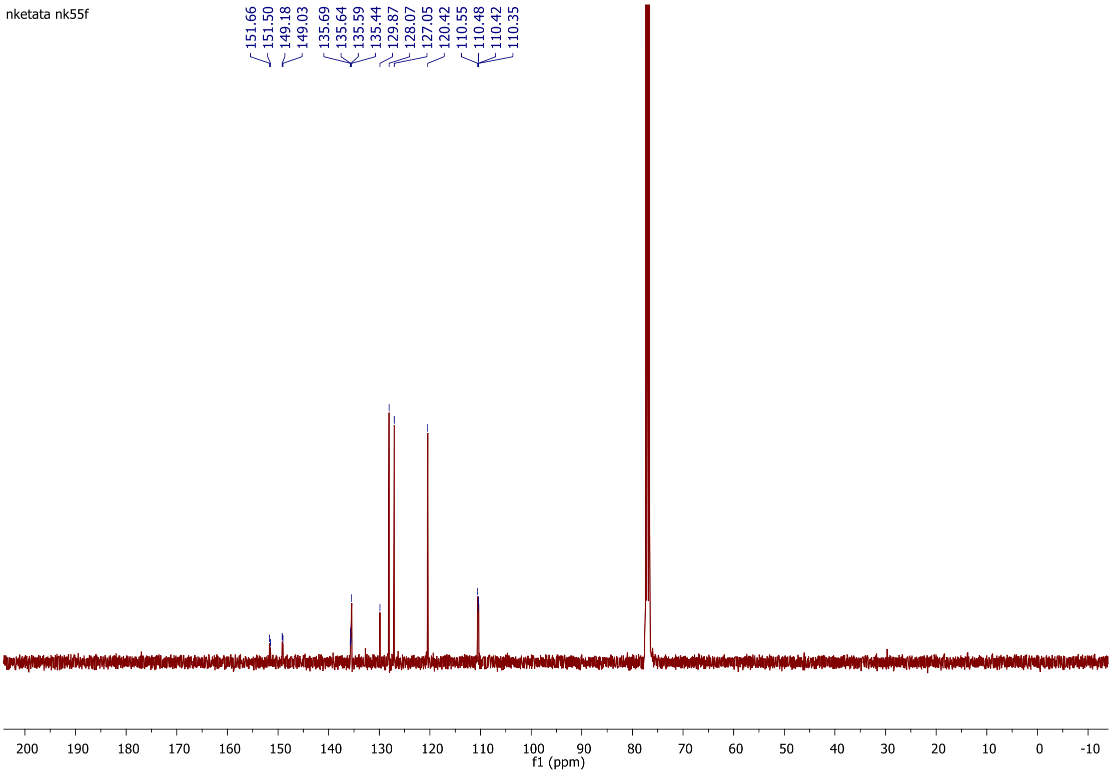

7,9-Difluorofluoranthene (26)

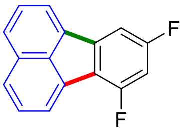

^{1}H NMR (400MHz, $CDCl_3$)

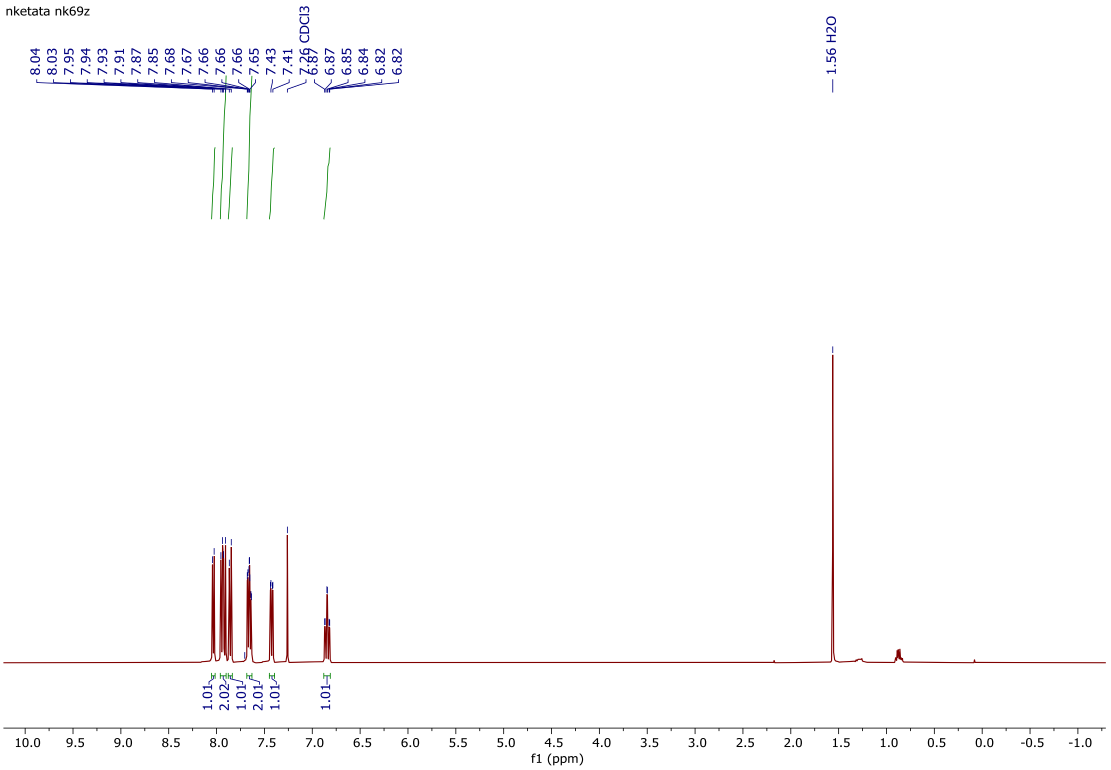

^{19}F NMR (376 MHz, $CDCl_3$)

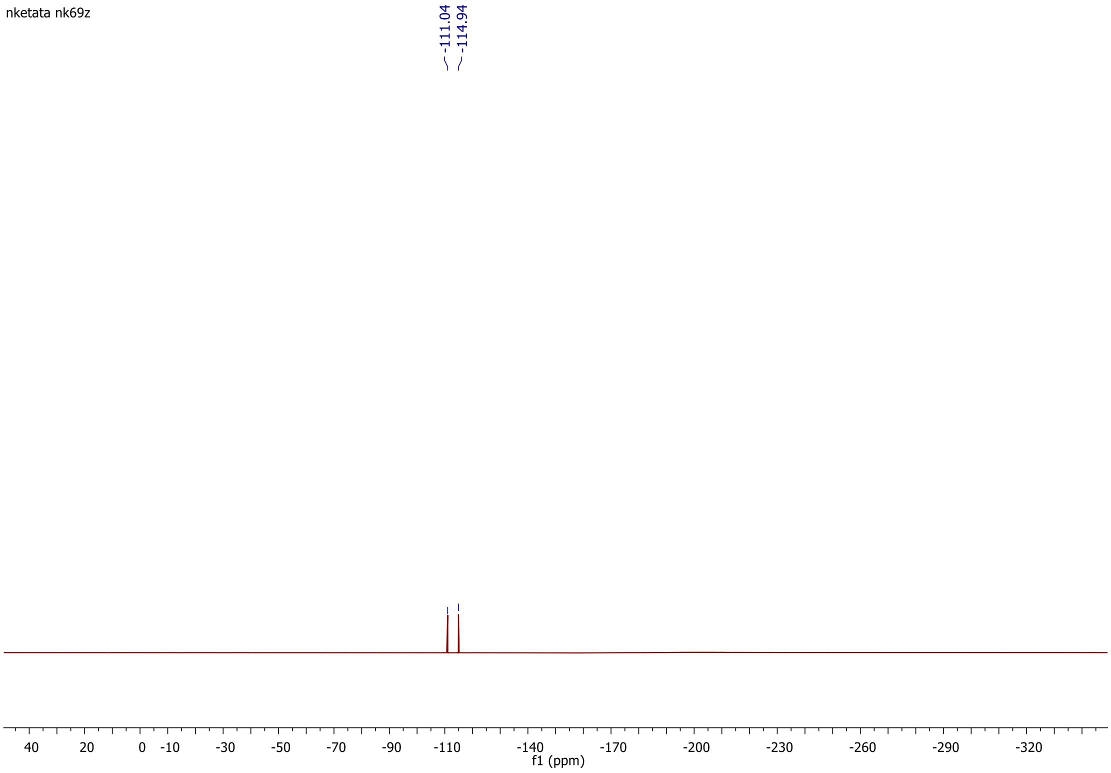

^{13}C NMR (100 MHz, $CDCl_3$)

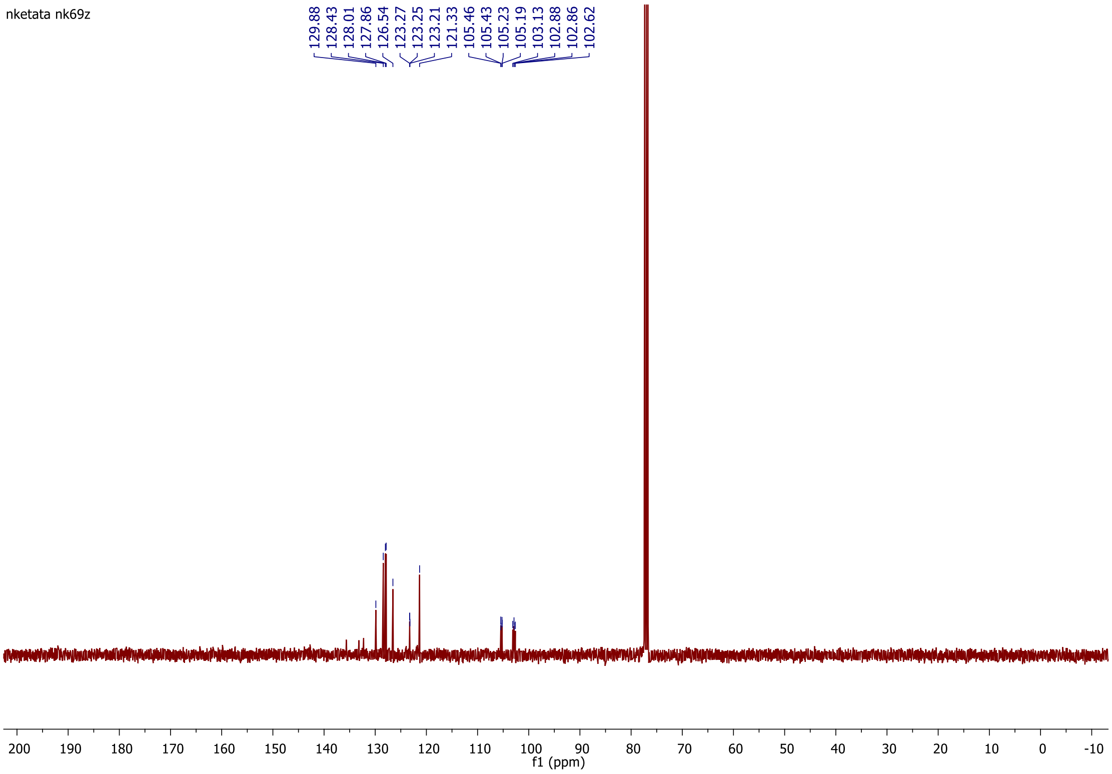

Acenaphtho[1,2-*c*]pyridine (27)

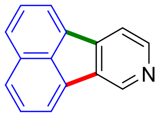

^{1}H NMR (400MHz, $CDCl_3$

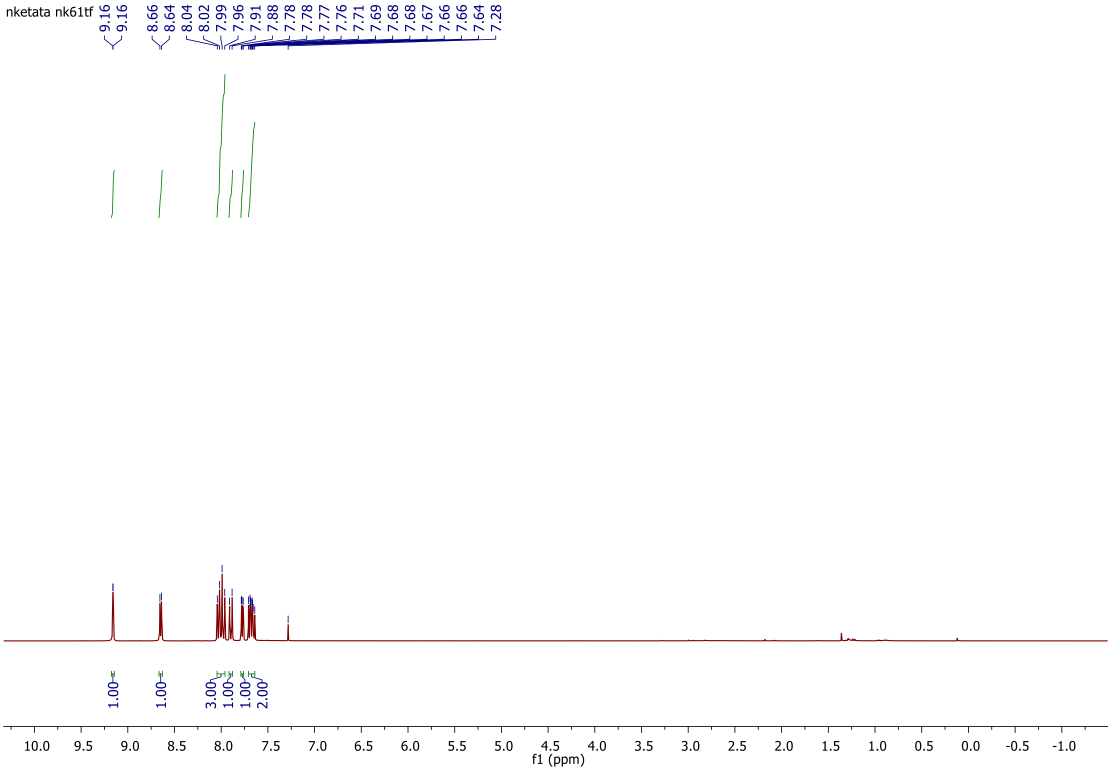

^{13}C NMR (100 MHz, $CDCl_3$)

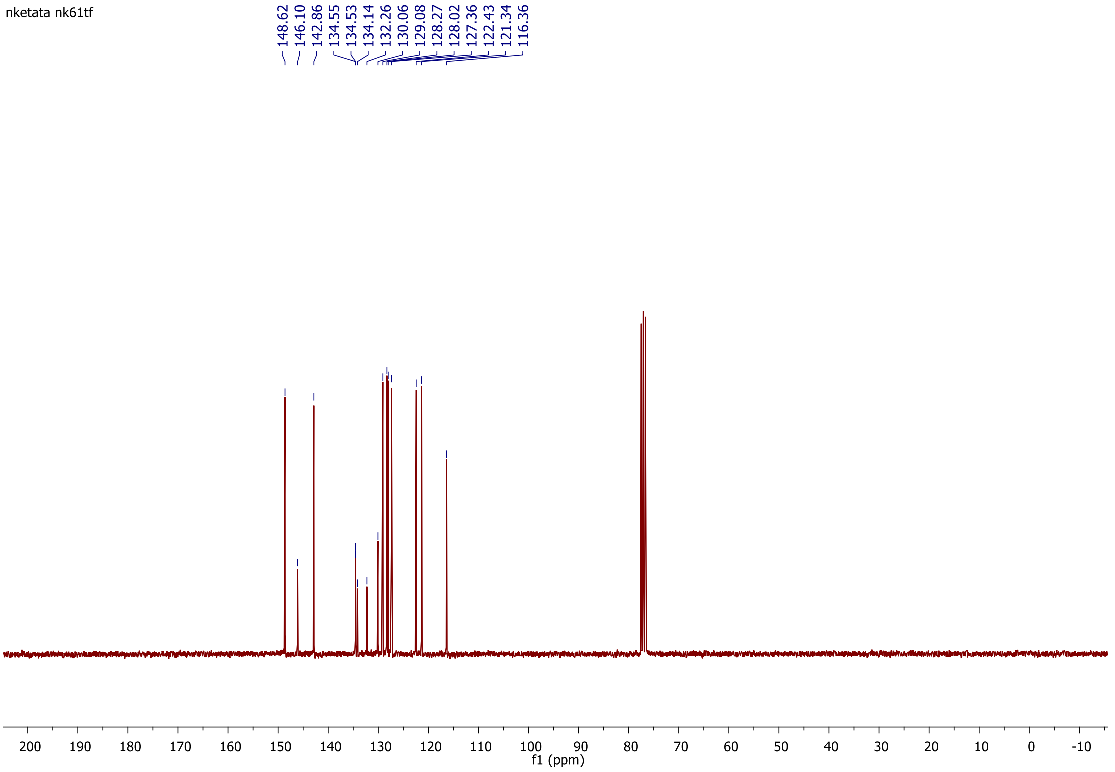

3-Methyl-9-phenylphenanthrene (28)

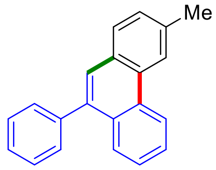

^{1}H NMR (400MHz, $CDCl_3$)

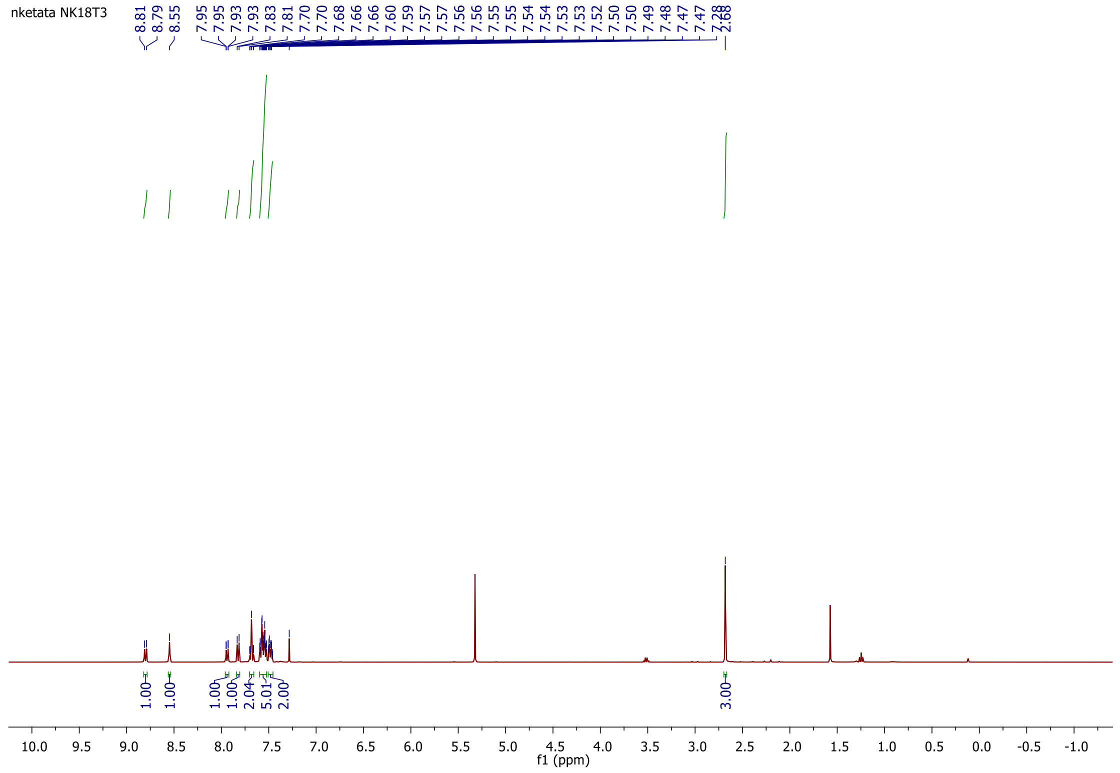

^{13}C NMR (100 MHz, $CDCl_3$)

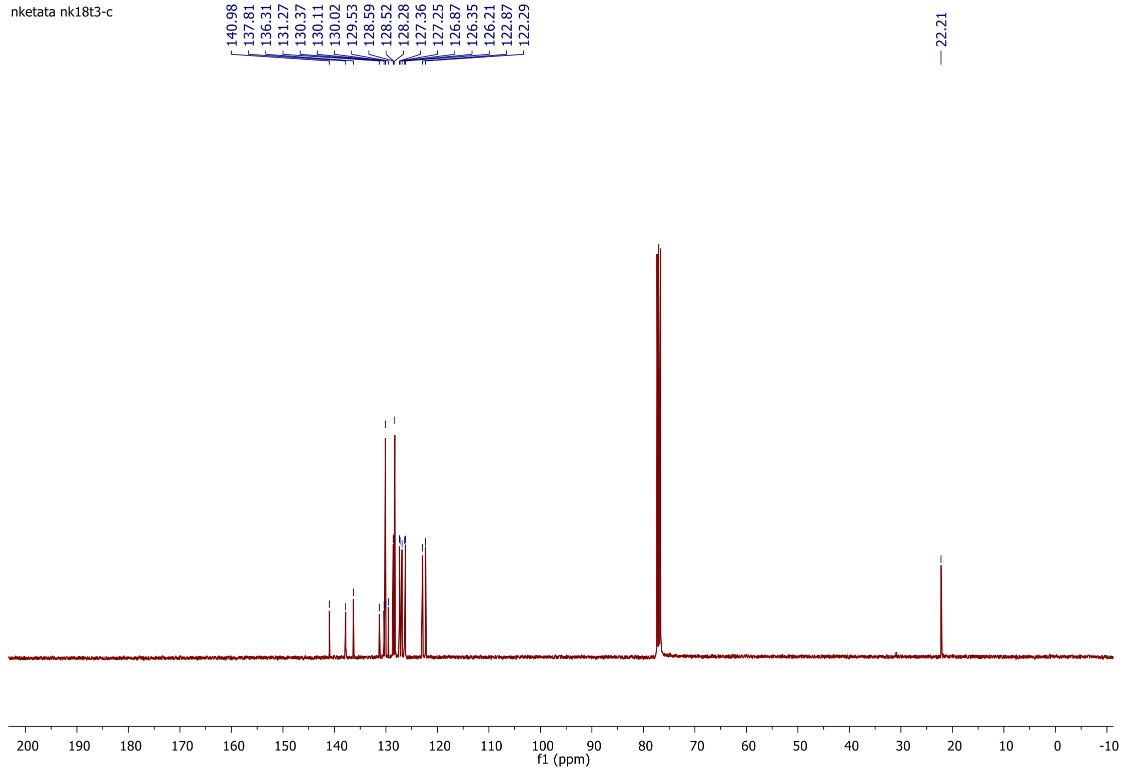

2-Methyl-9-phenylphenanthrene (29)

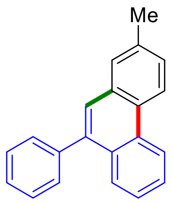

^{1}H NMR (400MHz, $CDCl_3$)

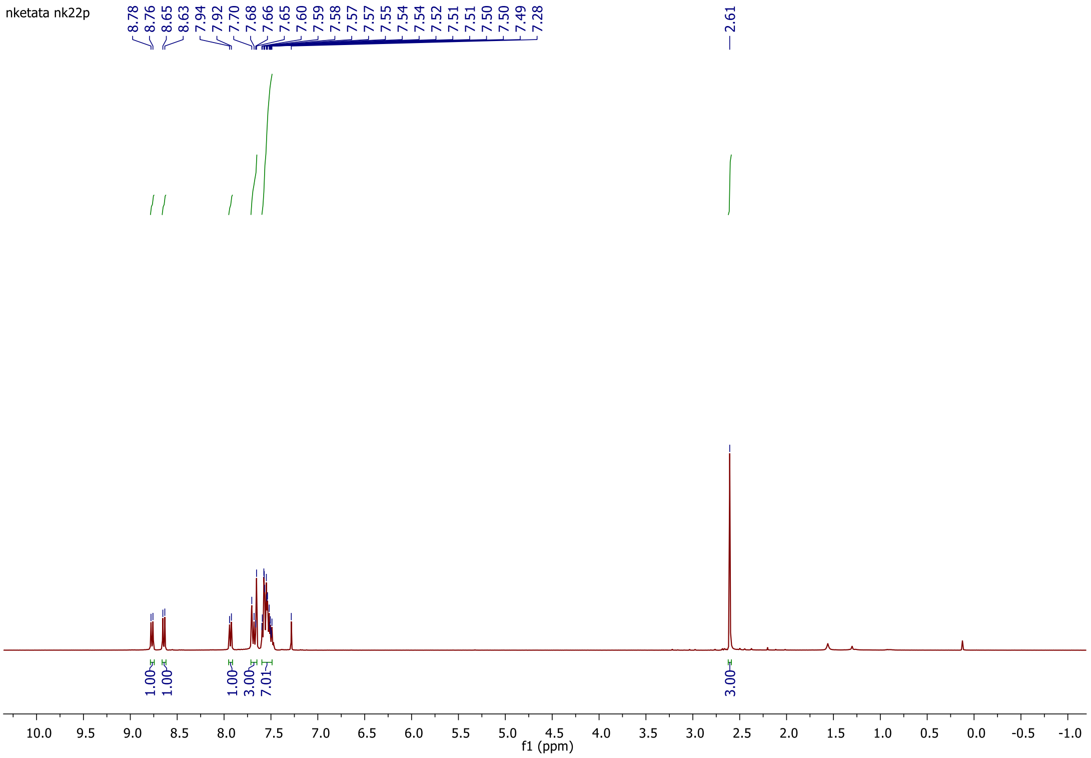

^{13}C NMR (100 MHz, $CDCl_3$)

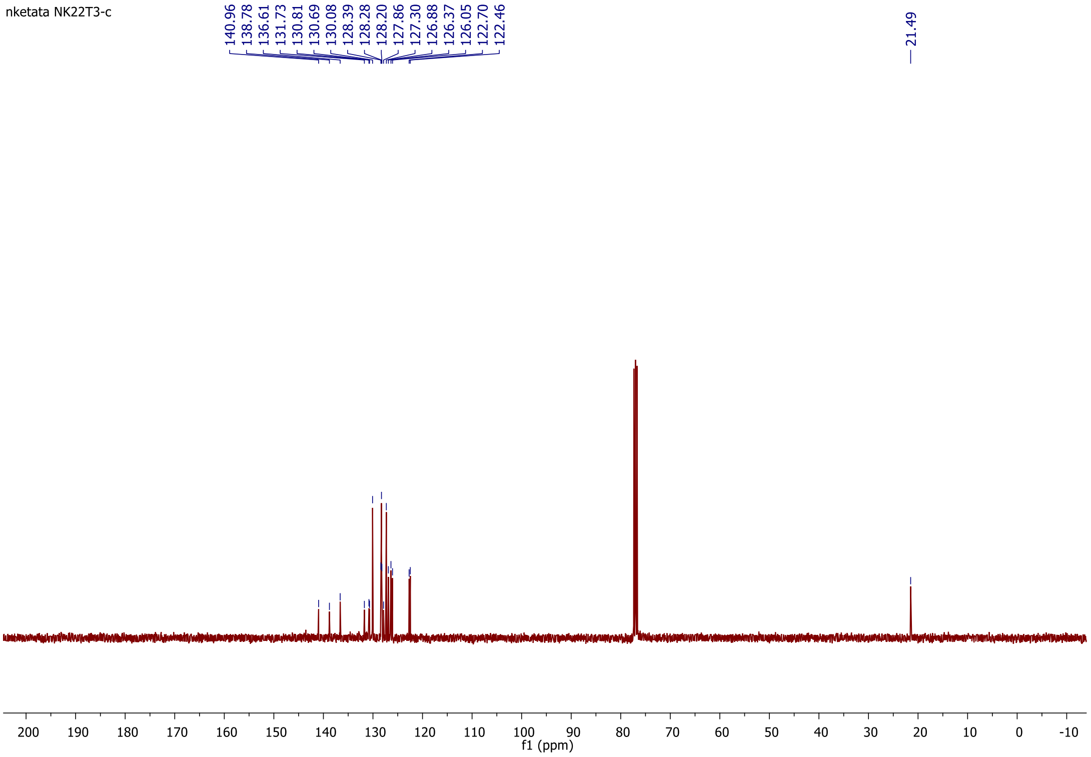

2,3-Dimethyl-9-phenylphenanthrene (30)

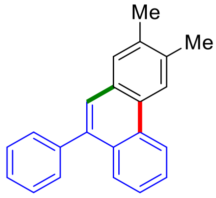

^{1}H NMR (400MHz, $CDCl_3$)

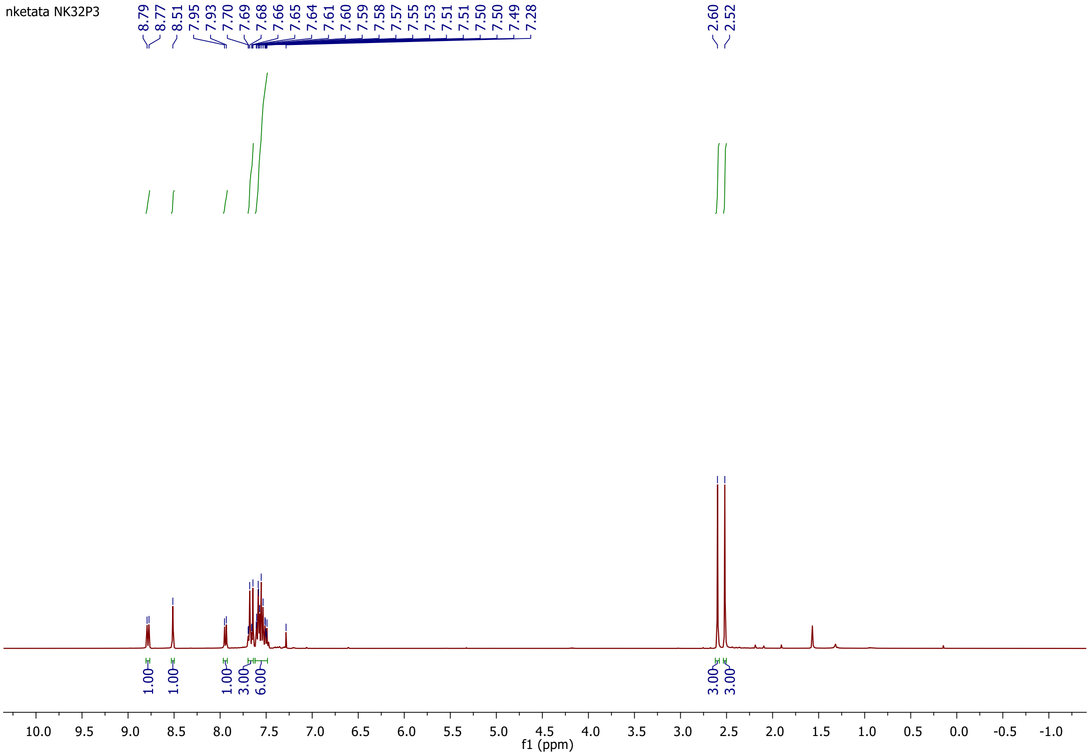

^{13}C NMR (100 MHz, $CDCl_3$)

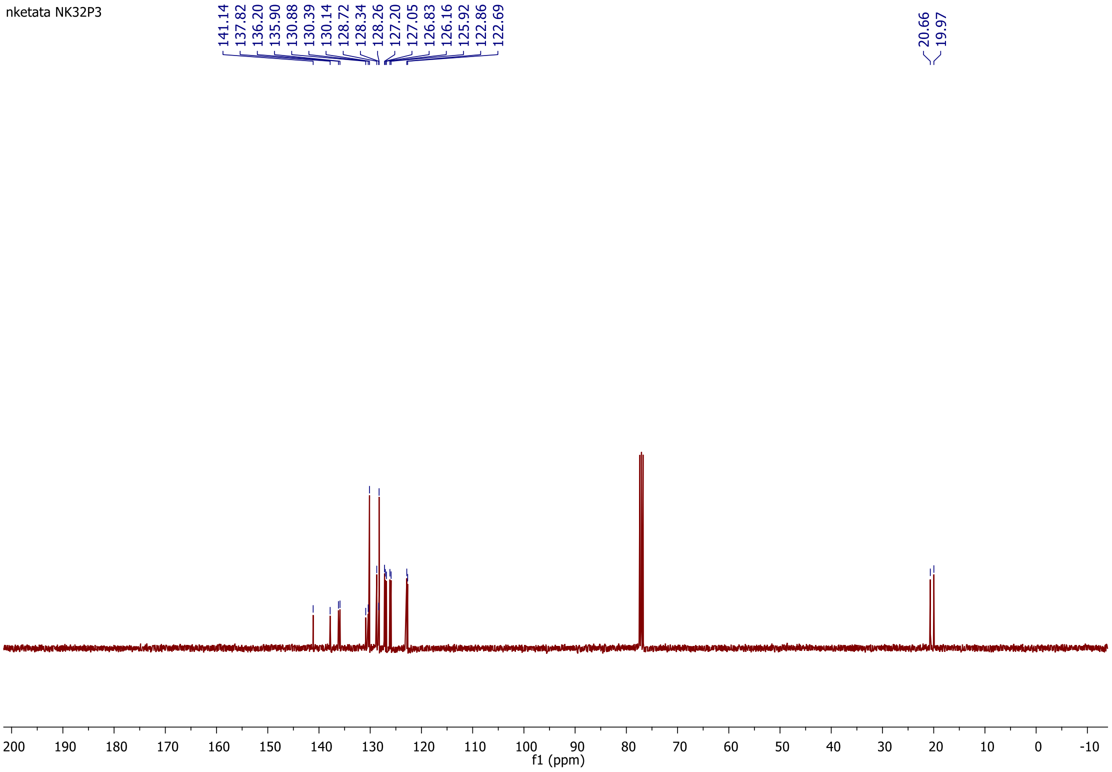

Supplement: File 2 — Copies of 1H, 19F, and 13C NMR spectra. [file Beilstein_J_Org_Chem-20-427-s002.pdf]
